# Global, regional, and national burden of low back pain, 1990–2020, its attributable risk factors, and projections to 2050: a systematic analysis of the Global Burden of Disease Study 2021

**DOI:** 10.1016/S2665-9913(23)00098-X

**Published:** 2023-05-22

**Authors:** GBD 2021 Low Back Pain Collaborators, Manuela L Ferreira, Katie de Luca, Lydia M Haile, Jaimie D Steinmetz, Garland T Culbreth, Marita Cross, Jacek A Kopec, Paulo H Ferreira, Fiona M Blyth, Rachelle Buchbinder, Jan Hartvigsen, Ai-Min Wu, Saeid Safiri, Anthony D Woolf, Gary S Collins, Kanyin Liane Ong, Stein Emil Vollset, Amanda E Smith, Jessica A Cruz, Kai Glenn Fukutaki, Semagn Mekonnen Abate, Mitra Abbasifard, Mohsen Abbasi-Kangevari, Zeinab Abbasi-Kangevari, Ahmed Abdelalim, Aidin Abedi, Hassan Abidi, Qorinah Estiningtyas Sakilah Adnani, Ali Ahmadi, Rufus Olusola Akinyemi, Abayneh Tadesse Alamer, Adugnaw Zeleke Alem, Yousef Alimohamadi, Mansour Abdullah Alshehri, Mohammed Mansour Alshehri, Hosam Alzahrani, Saeed Amini, Sohrab Amiri, Hubert Amu, Catalina Liliana Andrei, Tudorel Andrei, Benny Antony, Jalal Arabloo, Judie Arulappan, Ashokan Arumugam, Tahira Ashraf, Seyyed Shamsadin Athari, Nefsu Awoke, Sina Azadnajafabad, Till Winfried Bärnighausen, Lope H Barrero, Amadou Barrow, Akbar Barzegar, Lindsay M Bearne, Isabela M Bensenor, Alemshet Yirga Berhie, Bharti Bhandari Bhandari, Vijayalakshmi S Bhojaraja, Ali Bijani, Belay Boda Abule Bodicha, Srinivasa Rao Bolla, Javier Brazo-Sayavera, Andrew M Briggs, Chao Cao, Periklis Charalampous, Vijay Kumar Chattu, Flavia M Cicuttini, Benjamin Clarsen, Sarah Cuschieri, Omid Dadras, Xiaochen Dai, Lalit Dandona, Rakhi Dandona, Azizallah Dehghan, Takele Gezahegn G Demie, Edgar Denova-Gutiérrez, Syed Masudur Rahman Dewan, Samath Dhamminda Dharmaratne, Mandira Lamichhane Dhimal, Meghnath Dhimal, Daniel Diaz, Mojtaba Didehdar, Lankamo Ena Digesa, Mengistie Diress, Hoa Thi Do, Linh Phuong Doan, Michael Ekholuenetale, Muhammed Elhadi, Sharareh Eskandarieh, Shahriar Faghani, Jawad Fares, Ali Fatehizadeh, Getahun Fetensa, Irina Filip, Florian Fischer, Richard Charles Franklin, Balasankar Ganesan, Belete Negese Belete Gemeda, Motuma Erena Getachew, Ahmad Ghashghaee, Tiffany K Gill, Mahaveer Golechha, Pouya Goleij, Bhawna Gupta, Nima Hafezi-Nejad, Arvin Haj-Mirzaian, Pawan Kumar Hamal, Asif Hanif, Netanja I Harlianto, Hamidreza Hasani, Simon I Hay, Jeffrey J Hebert, Golnaz Heidari, Mohammad Heidari, Reza Heidari-Soureshjani, Mbuzeleni Mbuzeleni Hlongwa, Mohammad-Salar Hosseini, Alexander Kevin Hsiao, Ivo Iavicoli, Segun Emmanuel Ibitoye, Irena M Ilic, Milena D Ilic, Sheikh Mohammed Shariful Islam, Manthan Dilipkumar Janodia, Ravi Prakash Jha, Har Ashish Jindal, Jost B Jonas, Gebisa Guyasa Kabito, Himal Kandel, Rimple Jeet Kaur, Vikash Ranjan Keshri, Yousef Saleh Khader, Ejaz Ahmad Khan, Md Jobair Khan, Moien AB Khan, Hamid Reza Khayat Kashani, Jagdish Khubchandani, Yun Jin Kim, Adnan Kisa, Jitka Klugarová, Ali-Asghar Kolahi, Hamid Reza Koohestani, Ai Koyanagi, G Anil Kumar, Narinder Kumar, Tea Lallukka, Savita Lasrado, Wei-Chen Lee, Yo Han Lee, Ata Mahmoodpoor, Jeadran N Malagón-Rojas, Mohammad-Reza Malekpour, Reza Malekzadeh, Narges Malih, Man Mohan Mehndiratta, Entezar Mehrabi Nasab, Ritesh G Menezes, Alexios-Fotios A Mentis, Mohamed Kamal Mesregah, Ted R Miller, Mohammad Mirza-Aghazadeh-Attari, Maryam Mobarakabadi, Yousef Mohammad, Esmaeil Mohammadi, Shafiu Mohammed, Ali H Mokdad, Sara Momtazmanesh, Lorenzo Monasta, Mohammad Ali Moni, Ebrahim Mostafavi, Christopher J L Murray, Tapas Sadasivan Nair, Javad Nazari, Seyed Aria Nejadghaderi, Subas Neupane, Sandhya Neupane Kandel, Cuong Tat Nguyen, Ali Nowroozi, Hassan Okati-Aliabad, Emad Omer, Abderrahim Oulhaj, Mayowa O Owolabi, Songhomitra Panda-Jonas, Anamika Pandey, Eun-Kee Park, Shrikant Pawar, Paolo Pedersini, Jeevan Pereira, Mario F P Peres, Ionela-Roxana Petcu, Mohammadreza Pourahmadi, Amir Radfar, Shahram Rahimi-Dehgolan, Vafa Rahimi-Movaghar, Mosiur Rahman, Amir Masoud Rahmani, Nazanin Rajai, Chythra R Rao, Vahid Rashedi, Mohammad-Mahdi Rashidi, Zubair Ahmed Ratan, David Laith Rawaf, Salman Rawaf, Andre M N Renzaho, Negar Rezaei, Zahed Rezaei, Leonardo Roever, Guilherme de Andrade Ruela, Basema Saddik, Amirhossein Sahebkar, Sana Salehi, Francesco Sanmarchi, Sadaf G Sepanlou, Saeed Shahabi, Shayan Shahrokhi, Elaheh Shaker, MohammadBagher Shamsi, Mohammed Shannawaz, Saurab Sharma, Maryam Shaygan, Rahim Ali Sheikhi, Jeevan K Shetty, Rahman Shiri, Siddharudha Shivalli, Parnian Shobeiri, Migbar Mekonnen Sibhat, Ambrish Singh, Jasvinder A Singh, Helen Slater, Marco Solmi, Ranjani Somayaji, Ker-Kan Tan, Rekha Thapar, Seyed Abolfazl Tohidast, Sahel Valadan Tahbaz, Rohollah Valizadeh, Tommi Juhani Vasankari, Narayanaswamy Venketasubramanian, Vasily Vlassov, Bay Vo, Yuan-Pang Wang, Taweewat Wiangkham, Lalit Yadav, Ali Yadollahpour, Seyed Hossein Yahyazadeh Jabbari, Lin Yang, Fereshteh Yazdanpanah, Naohiro Yonemoto, Mustafa Z Younis, Iman Zare, Armin Zarrintan, Mohammad Zoladl, Theo Vos, Lyn M March

## Abstract

**Background:**

Low back pain is highly prevalent and the main cause of years lived with disability (YLDs). We present the most up-to-date global, regional, and national data on prevalence and YLDs for low back pain from the Global Burden of Diseases, Injuries, and Risk Factors Study 2021.

**Methods:**

Population-based studies from 1980 to 2019 identified in a systematic review, international surveys, US medical claims data, and dataset contributions by collaborators were used to estimate the prevalence and YLDs for low back pain from 1990 to 2020, for 204 countries and territories. Low back pain was defined as pain between the 12th ribs and the gluteal folds that lasted a day or more; input data using alternative definitions were adjusted in a network meta-regression analysis. Nested Bayesian meta-regression models were used to estimate prevalence and YLDs by age, sex, year, and location. Prevalence was projected to 2050 by running a regression on prevalence rates using Socio-demographic Index as a predictor, then multiplying them by projected population estimates.

**Findings:**

In 2020, low back pain affected 619 million (95% uncertainty interval 554–694) people globally, with a projection of 843 million (759–933) prevalent cases by 2050. In 2020, the global age-standardised rate of YLDs was 832 per 100 000 (578–1070). Between 1990 and 2020, age-standardised rates of prevalence and YLDs decreased by 10·4% (10·9–10·0) and 10·5% (11·1–10·0), respectively. A total of 38·8% (28·7–47·0) of YLDs were attributed to occupational factors, smoking, and high BMI.

**Interpretation:**

Low back pain remains the leading cause of YLDs globally, and in 2020, there were more than half a billion prevalent cases of low back pain worldwide. While age-standardised rates have decreased modestly over the past three decades, it is projected that globally in 2050, more than 800 million people will have low back pain. Challenges persist in obtaining primary country-level data on low back pain, and there is an urgent need for more high-quality, primary, country-level data on both prevalence and severity distributions to improve accuracy and monitor change.

**Funding:**

Bill and Melinda Gates Foundation.

## Introduction

The Global Burden of Diseases, Injuries, and Risk Factors Study (GBD) systematically quantifies health loss due to diseases and injuries by age, sex, year, and geographical location, and allows for the comparison of burden across disparate diseases.^1^ Previous GBD low back pain estimates^2^, ^3^, ^4^ confirmed that low back pain is the leading cause of disability in most countries. It is expected that both the total disability burden and disease-related costs will further increase in the coming decades.^5^

In response, global efforts have been made to provide clearer directions for change in policy and practice and to support the use of evidence-based management and prevention.^5^, ^6^, ^7^, ^8^ In 2018, *The Lancet* published a three-part Series on the definition, best-evidence-based treatment, and future research directions for low back pain. The Series highlighted the roles of advice and education that support self-management, physical, and psychological interventions, especially as first-line treatments for low back pain.^5^, ^7^, ^8^ However, there is still inappropriately high usage of imaging, prescribed bed rest, opioids, spinal injections, and other invasive procedures of questionable efficacy worldwide.^8^ Paradoxically, the use of treatments of no or little efficacy can delay recovery and potentially increase the risk of long-term back-related disability, and consequently increase the burden of this condition globally.

In the current report we present global, regional, and national-level estimates of prevalence and years lived with disability (YLDs) of low back pain in the general population. Estimates are reported in terms of numbers (count) and age-standardised rates, by age and sex, for 204 countries and territories, from 1990 to 2020. We also highlight the relative contribution of occupational factors, smoking, and high BMI to the prevalence and burden of low back pain and present projections of cases for 2030–50.

**Research in context**

**Evidence before this study**

The Global Burden of Diseases, Injuries, and Risk Factors Study (GBD) is a source of global, regional, and country-level estimates of disease burden over time. Input data are identified through systematic review in addition to US medical claims data. In 2017, it was estimated that over 551 million people were affected by low back pain, which was ranked the highest contributor to disability burden worldwide. To date, there is no published projection of the global prevalence of low back pain.

**Added value of this study**

The current analysis includes estimates from 1990 to 2020 with updated bias adjustments and prevalence projections through to 2050. In 2020, it was estimated that 619 million (95% uncertainty interval 554 to 694) people reported having low back pain globally. Between 1990 and 2020, there was a decrease in age-standardised rates of prevalence (–10·4%; –10·9 to –10·0) and years lived with disability (–10·5%; –11·1 to –10·0). Modifiable GBD risk factors, including occupational ergonomic factors, smoking, and high BMI, explained 38·8% (28·7 to 47·0) of years lived with disability. Prevalence projections for low back pain suggest that in 2050, there will be 843 million (759 to 933) individuals worldwide with low back pain, a 36·4% (29·9 to 43·2) increase from 2020.

**Implications of all the available evidence**

Low back pain continues to be the greatest cause of disability burden worldwide, and two-fifths of this burden has been attributed to modifiable risk factors. The decade 2020–30 has been designated the “United Nations Decade of Healthy Ageing”, and this initiative provides a strong platform to strengthen national, regional, and global health initiatives to decrease the burden of low back pain through public awareness campaigns and recommendations to keep active.

## Methods

### Overview

This manuscript was produced as part of the GBD Collaborator Network and in accordance with the GBD Protocol.^9^ GBD 2021 estimated low back pain prevalence and YLDs, by age and sex, for 204 countries and territories, from 1990 to 2020. The GBD study adheres to the Guidelines for Accurate and Transparent Health Estimates Reporting (GATHER) statement.^10^ Detailed methodology for the GBD study is reported elsewhere.^1^

### Case definition

The case definition for low back pain was pain in the posterior aspect of the body from the lower margin of the 12th ribs to the lower gluteal folds, with or without pain referred into one or both lower limbs, and lasting for at least 1 day.^3^

### Input data

Data were identified via systematic review of the literature of the electronic databases Ovid MEDLINE, Embase, and CINAHL, opportunistic searches, searches of government and international organisation websites, published reports, demographic and health surveys, and contributions of datasets by GBD collaborators. A systematic review of literature between 1980 and 2017 was updated on Oct 31, 2017, using the search terms “back pain”, “lumbar pain”, “back ache”, “backache”, and “lumbago” in combination with the terms “prevalence”, “incidence”, “cross-sectional”, and “epidemiology”, and on Oct 31, 2019, by searching PubMed using the terms “back pain”, “prevalence”, and “incidence”. Systematic reviews are not updated for all causes of disease in each GBD cycle, but rather updated on a rotational basis. There were no restrictions on age, sex, or language (appendix p 2). In the 2017 review, a total of eight new data sources were included, yielding a total of 19 studies. In 2019, 35 new sources were included, yielding a total of 455 citations (please see the GBD 2019 Data Input Sources Tool). In 2020, 36 additional sources of data on non-fatal low back pain were added (appendix pp 7–11). Moreover, surveys such as the World Health and Community Oriented Program for Control Of Rheumatic Diseases (COPCORD) surveys were included. In addition to literature data, US medical claims data from 2000 and 2010–17 were included based on ICD-9 and ICD-10 coding. The reference ICD-10 codes used to identify cases of low back pain were M54.3 (sciatica), M54.4 (lumbago with sciatica), and M54.5 (low back pain). The ICD-9 code was 724 (low back pain).

Each data source was given a unique identifier and included in the Global Health Data Exchange. Members of the core Institute of Health Metrics and Evaluation (IHME) research team for this topic area had full access to the underlying data used to generate estimates presented in this paper. All other authors had access to, and reviewed, estimates as part of the GBD and research evaluation process, which includes additional stages of internal IHME and external formal collaborator review. The global distribution of data sources of low back pain is presented in the appendix (p 29).

### Data processing and disease modelling

Before fitting models, data reported for wide age ranges and male and female sexes combined were split by age and sex. Sex-splitting and age data adjustments are described in the appendix (p 3). Data from studies that did not report sex-specific information were split based on a pooling of within-study sex ratios in a meta-regression tool, MR-BRT (meta-regression—Bayesian, regularised, trimmed; details described elsewhere^1^). The female-to-male ratio was 1·19 (95% uncertainty interval [UI] 1·03–1·40). Data that were reported in broad age groups (>25 years) were split into 5-year age groups by applying the age pattern estimated for low back pain in GBD 2019.

Data from sources that reported low back pain using alternative definitions (including studies that reported recall periods of 1 week to 1 month, recall periods between 1 month and 1 year, US claims data, and activity-limiting low back pain) were adjusted to the reference case definition. Bias adjustments were performed using MR-BRT. Adjustment factors were obtained by matching data with different case definitions by age, sex, year, and location and estimating the logit difference between the prevalence of the different case definitions in an MR-BRT network analysis, which leveraged matching pairs of data for two or more alternative case definitions (adjustment factors shown in appendix p 33). After adjustment, data with an age-standardised median absolute deviation of 1·5 or more above the mean prevalence by sex and location were considered outliers and excluded to ensure data that were implausibly high were not included in the analysis. The threshold of 1·5 or more above the mean prevalence was selected, given that outliers usually lie beyond 3 standard deviations from the mean.

A Bayesian meta-regression tool (DisMod-MR 2.1^1^) was used to generate estimates of prevalence by age, sex, location, and year. It was assumed there was no incident or prevalent low back pain before the age of 5 years. UIs were calculated by taking the 2·5th and 97·5th percentiles of the distribution of 1000 model runs after convergence.

Following this, estimates for low back pain were split by severity and presence of leg pain, based on proportions derived from Medical Expenditure Panel Surveys and US medical claims data (appendix p 36). Six different levels of severity were used for low back pain, two of which corresponded to health states with and without leg pain; each health state is linked to a disability weight (appendix p 35). The disability weight of each health state was multiplied by the corresponding age-sex-location-year-specific prevalence to calculate YLDs, which were adjusted based on co-occurrence of different diseases (details described elsewhere^1^). Global, regional (seven super-regions and 21 regions), and national prevalence and burden of disability rates are presented. GBD methodology does not attribute any increased mortality to low back pain in the current modelling. While low back pain might be part of the causal pathway for deaths (eg, opioid overdose), each death in GBD methodology can only be counted once and would be attributed to other causes. Therefore, our modelling results in identical estimates for disability-adjusted life-years and YLDs; thus, the burden of disability is reported as YLDs.

### Risk estimation

Occupational ergonomic factors, high BMI, and smoking were the risk factors included in GBD 2021 for which there was probable evidence of risk–outcome pairs (ie, more than one study type, at least two cohorts, no substantial and unexplained heterogeneity, low risk of confounding and selection bias, biologically acceptable dose–response gradients^11^). High BMI was defined as being greater than its theoretical minimum risk exposure level (ie, the level associated with the lowest risk as 20–25 kg/m^2^).^11^ Occupational ergonomic exposures were used as a proxy for lifting, forceful movements, awkward postures, and vibration, as specific data for these factors were not available for each country.^12^ Low back pain relative risks were derived from published population-representative data sources. Identified risk factors were encompassed in a summary exposure value (a normalised summary measure of all risk factors linked to a condition) that was used as a covariate to fit a DisMod-MR 2.1 model for the prevalence of low back pain. The summary exposure value compares the distribution of excess risk-exposure level to a population at maximum risk.

### Estimate projections

Forecast global and regional cases of low back pain to the year 2050 were computed by forecasting prevalence and population estimates.^13^ For low back pain, age-location-sex-specific GBD 2019 prevalence estimates from 1990 to 2020 were logit transformed and used in the following regression model:

$$E\left[\mathrm{logit}\left(Y_{l,a,s,y}\right)\right]=\beta_{1}\text{SDI}+\alpha_{l,a,s}$$

The term on the left side of the equation is the forecasted logit(prevalence), β_1_ is the fixed coefficient on Socio-demographic Index (SDI; a composite indicator of a country's lag-distributed income per capita, average years of schooling for those older than 15 years, and the total fertility rate of women aged 25 years or younger) over time, and α_l,a,s_ is the location-age-sex-specific random intercept. To obtain forecasted cases, forecasted rates were multiplied by forecasted population counts.^13^ Forecasted prevalence rates were intercept-shifted to GBD 2021 prevalence by subtracting forecasted estimation year 2020 prevalence rates from GBD 2021 estimation year 2020 prevalence rates and using this difference to shift all forecasted values through to the year 2050. A Das Gupta decomposition analysis was performed to determine the relative contributions to the change in case number between 2020 and 2050 of population growth, population ageing, and changes in prevalence unrelated to demographics.^14^ Validation testing was conducted using estimates from 1990 to 2010 to project prevalence from 2010 to 2019 by age, sex, location, and year. The projections were then compared with the GBD prevalence results for this period by calculating the summary root mean squared error (RMSE) and bias. Bias was calculated as the median value of all predicted minus observed values by age, sex, location, and year. In all the four tests the model RMSE was less than 0*·*01 and bias was less than 0*·*0001.

### Role of the funding source

The funder of the study had no role in the study design, data collection, data analysis, data interpretation, or writing of the report.

## Results

In 2020, the number of prevalent cases of low back pain globally was estimated at 619 million (95% UI 554–694; table 1), a substantial increase (60·4%; 57·1–64·2) from the 1990 aggregates for all ages and male and female sexes combined. The global age-standardised rate of prevalence of low back pain in 2020 was 7460 per 100 000 (6690–8370), representing a decrease of 10·4% (10·9–10·0) from 1990 (age-standardised rate 8330; 7470–9360). Globally, in 1990 low back pain accounted for 43·4 million (30·5–57·9) YLDs, for all ages and male and female sexes combined, representing 7·7% (6·4–8·7) of all-cause YLDs. In 2020, there were 69·0 million (47·9–88·9) low back pain YLDs, and although a slight decrease from 1990 in the percentage of all-cause YLDs worldwide (8·1%; 6·7–9·5), low back pain was still the main contributor to YLDs globally. Of the 21 GBD regions, the highest age-standardised rate of prevalence per 100 000 individuals for low back pain was found in central Europe (12 800; 11 500–14 400), followed by eastern Europe (11 200; 10 100–12 500) and Australasia (11 100; 9710–12 600), with east Asia presenting the lowest age-standardised rates of prevalence (5430; 4870–6110; table 1).

**Table 1:** Prevalence, YLDs, age-standardised rates of prevalence and YLDs per 100 000 population in 2020, and percentage change between 1990 and 2020 for low back pain globally, by GBD regions and super-regions

		**Number of prevalent cases**	**Age-standardised prevalence rate per 100 000**	**Percentage change in age-standardised prevalence rate from 1990 to 2020**	**Number of YLDs**	**Age-standardised rate of YLDs per 100 000**	**Percentage change in age-standardised rate of YLDs per 100 000 from 1990 to 2020**
Global		619 000 000(554 000 000 to 694 000 000)	7460(6690 to 8370)	–10·4%(–10·9 to –10·0)	69 000 000(47 900 000 to 88 900 000)	832·0(578·0 to 1070·0)	–10·5%(–11·1 to –10·0)
Central Europe, eastern Europe, and central Asia		60 300 000(53 800 000 to 67 100 000)	11 200(10 100 to 12 600)	–4·7%(–5·3 to –4·2)	6 690 000(4 690 000 to 8 730 000)	1250·0(873·0 to 1620·0)	–4·5%(–5·2 to –3·9)
	Central Asia	8 050 000(7 050 000 to 9 120 000)	9110(8080 to 10 200)	–1·1%(–2·7 to 0·5)	910 000(633 000 to 1 200 000)	1020·0(710·0 to 1320·0)	–1·1%(–3·2 to 0·8)
	Central Europe	20 300 000(18 200 000 to 22 600 000)	12 800(11 500 to 14 400)	–2·5%(–3·4 to –1·6)	2 260 000(1 590 000 to 2 970 000)	1440·0(1000·0 to 1870·0)	–2·1%(–3·1 to –1·1)
	Eastern Europe	31 900 000(28 400 000 to 35 600 000)	11 200(10 100 to 12 500)	–3·9%(–4·7 to –2·9)	3 520 000(2 480 000 to 4 600 000)	1240·0(865·0 to 1600·0)	–3·8%(–4·8 to –2·6)
High-income		144 000 000(131 000 000 to 156 000 000)	9880(9000 to 10 900)	–4·2%(–5·9 to –2·5)	16 000 000(11 100 000 to 20 700 000)	1100·0(768·0 to 1410·0)	–4·7%(–6·3 to –3·1)
	Australasia	4 130 000(3 650 000 to 4 650 000)	11 100(9710 to 12 600)	–5·8%(–9·1 to –2·2)	460 000(320 000 to 590 000)	1240·0(861·0 to 1580·0)	–5·6%(–9·5 to –1·7)
	High-income Asia Pacific	26 500 000(23 700 000 to 29 300 000)	9690(8590 to 10 900)	–7·7%(–8·8 to –6·5)	2 970 000(2 050 000 to 3 930 000)	1100·0(762·0 to 1420·0)	–7·5%(–8·6 to –6·1)
	High-income North America	48 600 000(45 200 000 to 51 600 000)	10 500(9900 to 11 200)	–5·8%(–11·3 to –0·7)	5 320 000(3 800 000 to 6 790 000)	1160·0(816·0 to 1470·0)	–7·0%(–12·5 to –2·3)
	Southern Latin America	7 080 000(6 290 000 to 7 950 000)	9280(8240 to 10 500)	1·7%(–1·1 to 5·3)	791 000(550 000 to 1 010 000)	1040·0(723·0 to 1320·0)	1·3%(–1·4 to 5·1)
	Western Europe	57 900 000(51 500 000 to 64 000 000)	9510(8480 to 10 700)	–2·2%(–4·3 to –0·5)	6 410 000(4 450 000 to 8 420 000)	1070·0(737·0 to 1360·0)	–2·3%(–4·3 to –0·6)
Latin America and Caribbean		48 800 000(43 100 000 to 55 100 000)	7860(6960 to 8850)	1·9%(1·3 to 2·8)	5 430 000(3 760 000 to 6 970 000)	874·0(605·0 to 1120·0)	1·7%(0·8 to 2·7)
	Andean Latin America	3 610 000(3 240 000 to 4 070 000)	5750(5180 to 6460)	0·6%(–1·9 to 3·3)	405 000(280 000 to 511 000)	644·0(446·0 to 813·0)	0·2%(–2·6 to 3·2)
	Caribbean	3 030 000(2 700 000 to 3 360 000)	5950(5300 to 6600)	–1·0%(–3·0 to 1·1)	338 000(237 000 to 433 000)	664·0 (465·0 to 845·0)	–1·5%(–3·5 to 0·3)
	Central Latin America	19 300 000(17 100 000 to 21 900 000)	7480(6640 to 8450)	1·2%(–0·2 to 2·5)	2 160 000(1 490 000 to 2 770 000)	835·0 576·0 to 1070·0)	1·2%(–0·2 to 2·9)
	Tropical Latin America	22 800 000(20 200 000 to 25 800 000)	9190(8130 to 10 400)	3·2%(2·0 to 4·4)	2 530 000(1 750 000 to 3 260 000)	1020·0(704·0 to 1300·0)	2·9%(1·5 to 4·3)
North Africa and Middle East		50 500 000(44 600 000 to 57 500 000)	8720(7770 to 9780)	–2·0%(–3·1 to –0·8)	5 640 000(3 900 000 to 7 300 000)	967·0 670·0 to 1240·0)	–2·6%(–3·9 to –1·3)
South Asia		117 000 000(103 000 000 to 134 000 000)	6950(6170 to 7910)	–10·4%(–11·5 to –9·4)	13 000 000(9 050 000 to 16 700 000)	765·0(534·0 to 988·0)	–10·0%(–11·2 to –9·2)
Southeast Asia, east Asia, and Oceania		146 000 000(129 000 000 to 164 000 000)	5560(4970 to 6260)	–14·5%(–15·4 to –13·4)	16 500 000(11 400 000 to 21 600 000)	627·0(433·0 to 809·0)	–14·3%(–15·4 to –13·3)
	East Asia	104 000 000(92 600 000 to 117 000 000)	5430(4870 to 6110)	–18·5%(–19·8 to –17·0)	11 800 000(8 180 000 to 15 500 000)	614·0(423·0 to 794·0)	–18·3%(–19·8 to –17·0)
	Oceania	653 000(565 000 to 750 000)	6340(5550 to 7140)	–0·8%(–4·0 to 2·7)	73 800(51 400 to 97 100)	707·0(493·0 to 916·0)	–0·8%(–3·9 to 3·0)
	Southeast Asia	40 700 000(35 800 000 to 46 600 000)	5880(5230 to 6650)	–1·5%(–2·4 to –0·2)	4 610 000(3 190 000 to 5 990 000)	661·0(458·0 to 850·0)	–1·2%(–2·3 to 0·4)
Sub-Saharan Africa		52 100 000(45 700 000 to 59 000 000)	7180(6410 to 8020)	–3·0%(–3·4 to –2·5)	5 840 000(4 040 000 to 7 540 000)	796·0(554·0 to 1030·0)	–2·6%(–3·2 to –2·0)
	Central sub-Saharan Africa	6 490 000(5 720 000 to 7 400 000)	7480(6700 to 8400)	–3·4%(–6·2 to –1·0)	723 000(503 000 to 937 000)	824·0(577·0 to 1060·0)	–2·9%(–6·1 to –0·7)
	Eastern sub-Saharan Africa	20 300 000(17 800 000 to 22 900 000)	7600(6800 to 8460)	–3·5%(–4·3 to –2·7)	2 270 000(1 580 000 to 2 940 000)	843·0(587·0 to 1090·0)	–2·9%(–3·9 to –1·9)
	Southern sub-Saharan Africa	4 520 000(3 990 000 to 5 140 000)	6510(5800 to 7310)	–4·8%(–5·8 to –3·8)	500 000(346 000 to 648 000)	714·0(494·0 to 929·0)	–5·7%(–7·0 to –4·1)
	Western sub-Saharan Africa	20 900 000(18 200 000 to 23 700 000)	6890(6130 to 7700)	–2·5%(–3·2 to –1·9)	2 340 000(1 620 000 to 3 030 000)	766·0(532·0 to 991·0)	–2·1%(–2·8 to –1·3)

Data in parentheses are 95% uncertainty intervals. Region and super-region numbers do not sum to the global prevalence due to rounding. YLDs=years lived with disability.

**Figure 1:**
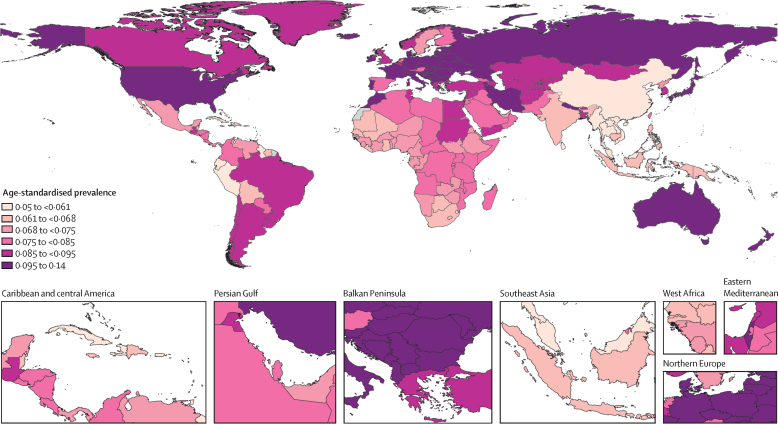
Age-standardised prevalence of low back pain by country for male and female sexes combined and all ages in 2020

Age-standardised rates of low back pain prevalence for 204 countries and territories in 2020 are found in figure 1 and in the appendix (pp 37–52). The nations with the highest age-standardised rates of prevalence per 100 000 were Hungary (14 000; 95% UI 12 600 to 15 500) followed by Czechia (13 100; 11 600 to 14 700), whereas the ones with the lowest age-standardised rates of prevalence were Maldives (5050; 4460 to 5730) and Myanmar (5090; 4530 to 5780). China had the largest percentage decrease in age-standardised rates of prevalence (–19·4%; –20·7 to –18·0) and YLDs (–19·3%; –20·8 to –17·8) per 100 000 between 1990 and 2020 (appendix pp 37–52). The largest increase in age-standardised rate of prevalence (19·4%; 12·1 to 27·2) and YLDs (20·0%; 12·5 to 28·4) was seen in Sweden (appendix pp 37–52).

**Figure 2:**
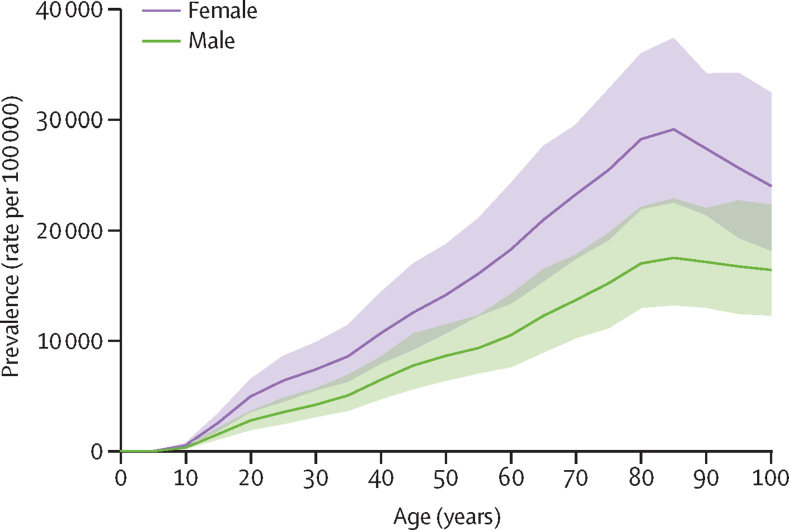
Global prevalence of low back pain by age and sex in 2020 Shaded areas represent 95% uncertainty intervals.

Global prevalence rates were higher among females compared with males (figure 2) across all age groups, although more marked differences were observed at older age groups (ie, >75 years of age). The global age-standardised rate of prevalence per 100 000 was also higher in females (9330; 95% UI 8370–10 500) compared with males (5520; 4930–6190). Prevalence and YLDs increased with age, with peak prevalence rates observed at approximately 85 years of age (figure 2). Globally, the age group 80–84 years had the highest YLD rate per 100 000 (2440; 1470–3490).

Prevalence and YLD counts and age-standardised rates per 100 000 population in 2020, and corresponding percentage changes between 1990 and 2020, for male and female sexes combined, by GBD super-regions (central Europe, eastern Europe, and central Asia; high-income; Latin America and Caribbean; north Africa and Middle East; south Asia; southeast Asia, east Asia, and Oceania; and sub-Saharan Africa), are presented in table 1. The highest age-standardised rate of low back pain prevalence per 100 000 was observed in the central Europe, eastern Europe, and central Asia super-region (11 200; 95% UI 10 100 to 12 600), with the lowest age-standardised rate of prevalence per 100 000 observed in the southeast Asia, east Asia, and Oceania super-region (5560; 4970 to 6260). Similarly, the highest age-standardised rate of YLDs per 100 000 was observed in the central Europe, eastern Europe, and central Asia super-region (1250; 873 to 1620), whereas the lowest age-standardised rate was observed in the southeast Asia, east Asia, and Oceania super-region (627; 433 to 809; table 1). A small decrease in the age-standardised rates of prevalence between 1990 and 2020 was observed for all super-regions, except for the Latin America and Caribbean super-region, which had a small percentage increase of 1·9% (1·3 to 2·8). Countries of the southeast Asia, east Asia, and Oceania super-region presented the largest decrease (–14·5%; –15·4 to –13·4). A similar pattern was seen for YLDs (table 1).

In 2020, 38·8% (95% UI 28·7–47·0) of global YLDs due to low back pain were attributable to exposure to three modifiable GBD risk factors. Globally, and across all ages and male and female sexes combined, 22·0% (20·4–23·4) of YLDs were attributable to occupational ergonomic factors, 12·5% (3·1–21·5) to smoking, and 11·5% (1·4–20·9) to high BMI. These represent a total of 194 YLDs (137–261) per 100 000 population attributed to occupational risks; 110 (29–192) to smoking, and 102 (11–195) to high BMI. The risk of low back pain attributed to smoking was highest among middle-aged (ie, 50–69 years of age) males (28·8%; 7·5–48·9) and lowest among females aged 15–49 years (5·7%; 1·3–10·3), whereas the risk attributed to occupational ergonomic factors was highest among younger (ie, 15–49 years of age) male adults (34·3%; 31·9–36·6) and lowest among females 70 years of age or older (4·9%; 3·8–6·0). The risk of low back pain attributed to high BMI was, however, highest among females aged 50–69 years (14·5%; 1·8–26·2) and lowest among younger (ie, 15–49 years) males (9·8%; 1·2–17·5).

**Figure 3:**
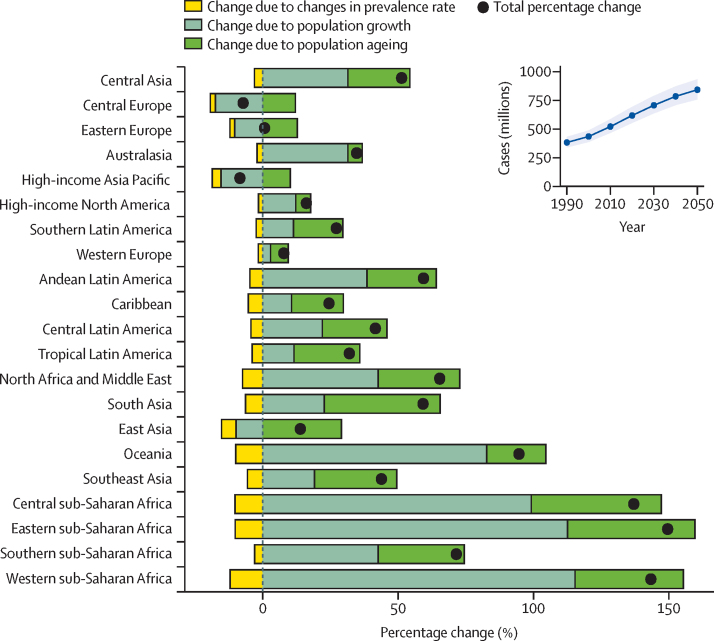
Decomposition of projected change in the number of prevalent low back pain cases between 2020 and 2050

Based on forecasted changes in population, in 2050 there will be 843 million (95% UI 759–933) individuals worldwide with low back pain (table 2) or an increase in total cases of 36·4% (29·9–43·2) globally (figure 3). The projected increase in number of cases globally was similar among males and females (appendix p 30). While most regions had a greater than 25% projected increase in cases between 2020 and 2050, central Europe, eastern Europe, and high-income Asia Pacific had a total projected decrease in cases over that period (table 2). A decomposition analysis by region and globally showed the greatest contribution of population growth, followed by population ageing, to the projected increase in number of cases by 2050 (figure 3). This was true for all regions except for east Asia, south Asia, tropical Latin America, southern Latin America, and the Caribbean; in these regions, the greatest contribution to the projected increase in number of cases appeared to come from population ageing.

**Table 2:** Age-standardised prevalence and cases of low back pain projections to 2030, 2040, and 2050, globally and by region, male and female sexes combined

	**Age-standardised prevalence (%)**			**Cases (millions)**		
	2030	2040	2050	2030	2040	2050
Global	7·30% (6·52–8·21)	7·17% (6·38–8·08)	7·08% (6·28–7·99)	707 (638–788)	785 (710–872)	843 (759–933)
Andean Latin America	5·68% (5·10–6·38)	5·60% (5·02–6·30)	5·53% (4·96–6·24)	4·37 (3·90–4·89)	5·11 (4·50–5·73)	5·74 (4·99–6·52)
Australasia	11·0% (9·65–12·5)	10·9% (9·58–12·4)	10·9% (9·50–12·4)	4·61 (4·11–5·16)	5·09 (4·51–5·70)	5·49 (4·83–6·21)
Caribbean	5·86% (5·20–6·52)	5·76% (5·10–6·41)	5·66% (4·99–6·32)	3·38 (3·04–3·71)	3·65 (3·29–4·00)	3·80 (3·39–4·23)
Central Asia	9·05% (8·01–10·1)	8·96% (7·92–10·1)	8·88% (7·84–9·98)	9·45 (8·40–10·6)	10·6 (9·45–11·9)	11·6 (10·3–13·0)
Central Europe	12·7% (11·4–14·3)	12·6% (11·3–14·2)	12·5% (11·2–14·2)	20·0 (17·9–22·3)	19·2 (17·4–21·3)	18·0 (16·3–20·0)
Central Latin America	7·38% (6·54–8·34)	7·27% (6·43–8·23)	7·17% (6·36–8·13)	22·4 (20·0–25·1)	24·9 (22·2–27·9)	26·5 (23·5–29·9)
Central sub-Saharan Africa	7·31% (6·51–8·23)	7·16% (6·36–8·08)	7·04% (6·24–7·96)	8·84 (7·80–9·82)	11·7 (10·2–12·9)	14·9 (13·0–16·7)
East Asia	5·33% (4·77–6·01)	5·25% (4·68–5·93)	5·16% (4·58–5·84)	116 (104–129)	120 (108–135)	116 (104–129)
Eastern Europe	11·1% (9·95–12·4)	11·0% (9·83–12·3)	10·9% (9·74–12·3)	32·4 (28·9–36·2)	32·1 (28·6–36·0)	31·3 (27·8–35·2)
Eastern sub-Saharan Africa	7·44% (6·63–8·29)	7·30% (6·48–8·14)	7·18% (6·36–8·02)	27·7 (24·6–30·7)	37·1 (32·9–41·4)	48·2 (42·2–54·3)
High-income Asia Pacific	9·57% (8·45–10·8)	9·46% (8·34–10·7)	9·38% (8·27–10·6)	26·6 (23·8–29·4)	25·7 (23·0–28·4)	24·0 (21·6–26·6)
High-income North America	10·4% (9·83–11·1)	10·4% (9·76–11·0)	10·3% (9·68–11·0)	53·2 (49·7–56·3)	55·8 (52·5–59·7)	57·0 (53·4–61·4)
North Africa and Middle East	8·55% (7·59–9·60)	8·38% (7·41–9·43)	8·25% (7·28–9·29)	61·6 (54·7–69·4)	72·4 (64·7–81·0)	81·8 (72·7–91·9)
Oceania	6·25% (5·46–7·04)	6·12% (5·33–6·91)	5·98% (5·19–6·76)	0·848 (0·734–0·954)	1·08 (0·934–1·21)	1·34 (1·14–1·53)
South Asia	6·82% (6·04–7·78)	6·70% (5·91–7·66)	6·61% (5·80–7·57)	138 (123–156)	159 (143–175)	176 (158–191)
Southeast Asia	5·78% (5·13–6·53)	5·68% (5·04–6·43)	5·59% (4·96–6·34)	49·1 (44·1–55·1)	56·6 (51·1–62·7)	61·9 (55·5–68·4)
Southern Latin America	9·21% (8·17–10·4)	9·13% (8·10–10·3)	9·06% (8·03–10·2)	7·80 (6·98–8·71)	8·34 (7·49–9·29)	8·63 (7·72–9·58)
Southern sub-Saharan Africa	6·45% (5·73–7·26)	6·40% (5·67–7·21)	6·35% (5·62–7·20)	5·48 (4·90–6·11)	6·50 (5·83–7·27)	7·49 (6·71–8·42)
Tropical Latin America	9·07% (8·01–10·2)	8·96% (7·89–10·1)	8·87% (7·79–10·0)	25·5 (22·6–28·5)	27·3 (24·4–30·5)	28·2 (25·3–31·3)
Western Europe	9·44% (8·41–10·6)	9·39% (8·34–10·6)	9·34% (8·28–10·5)	60·4 (53·3–66·8)	61·7 (54·8–68·2)	61·4 (54·4–67·7)
Western sub-Saharan Africa	6·73% (5·97–7·55)	6·59% (5·80–7·39)	6·45% (5·64–7·25)	28·6 (25·2–32·0)	38·3 (33·6–42·9)	50·1 (44·1–56·9)

Data in parentheses are 95% uncertainty intervals. Region numbers do not sum to the global prevalence due to rounding.

## Discussion

This report presents estimates of low back pain prevalence and burden at global, regional, and national levels. In 2020, there were more than half a billion prevalent cases of low back pain worldwide, representing 7·7% of all YLDs and thus the greatest contribution to the world's burden of disability. By 2050, a 36·4% increase in total number of cases of low back pain is expected globally, with the most substantial increases expected to be seen in Asia and Africa. Decomposition analyses show that most of the increase in prevalence will be driven by population growth, except in some regions where population ageing seems to be the main reason for the rise in the number of low back pain cases by 2050.

The high rate of low back pain prevalence observed in all regions globally could have some important social and economic consequences, especially considering the substantial cost of care for this condition. For instance, from 2012 to 2014, the direct aggregate costs for all individuals with a spine condition in the USA were US$315 billion,^15^ with a substantial proportion of costs attributed to surgical procedures. Moreover, prescription medications for spine conditions in Australia showed a marked rise in the past years, with opioids becoming the most commonly prescribed class of drugs for low back pain.^16^ Opioids are now well recognised to be responsible for important adverse health events, including high rates of addiction, accidental overdose, and death,^17^ resulting in additional costs to the individual and society due to medical care for opioid abuse and loss of productivity.^18^ While somewhat speculative, it is possible that improving access to effective non-pharmacological care for low back pain might reduce some of the impact of the opioid epidemic.

A further societal and economic impact of low back pain stems from its high prevalence and substantial burden in working-age people—a problem certainly not exclusive to high-income countries. An average of 100 days absent from work per person per year were due to low back pain in Brazil, with productivity losses equating to 79% of the US$2·2 billion cost of low back pain.^19^ In the USA, 15·4% of the workforce report an average of 10·5 lost workdays per year due to chronic low back pain. This is equivalent to approximately 264 million workdays lost.^20^ On top of absenteeism from work, low back pain might force workers to retire prematurely.^21^ People who retire early because of low back pain have substantially less total wealth and income-producing assets than those who remain in full-time employment.^22^ Therefore, more emphasis should be given to integrated and early return to work interventions. As suggested in the literature, these interventions should include those based around cognitive behavioural therapy principles, problem-solving skills, and ergonomics, with involvement at the supervisor level. These interventions should lead to fewer disability days, higher rates of and earlier return to work, and reductions in use of health care.^6^, ^23^, ^24^ Moreover, initiatives that focus on work environments involving manual labour, and those in low-income and middle-income countries, are urgently needed.

We have seen a modest decrease from 1990 in the age-standardised rate of prevalence and YLDs of low back pain. While our data cannot be used to ascertain the reason for the decrease, it is possible that it represents a shift in the incidence of low back pain perhaps due to changes in manual labour or increased recovery. It is important to remember, however, that low back pain remains the main contributor to disability worldwide, and global strategies to reduce the number of new episodes of low back pain and its associated disability are key. Nevertheless, there is scarce evidence supporting prevention strategies for low back pain, especially in specific populations such as older patients, or those targeting low-income and middle-income countries. Focused solutions include public health prevention strategies, particularly those that are affordable in and relevant to low-income and middle-income countries, and will first need widespread testing and implementation.

Challenges in addressing the burden of low back pain in both high-income countries and low-income and middle-income countries are different. Aligning health care to adhere to clinical guidelines to reduce excessive opioid use and unnecessary and costly surgical treatments is imperative. As treatment effectiveness data come nearly exclusively from high-income countries, the cultural suitability of guideline recommendations for low-income and middle-income countries is not known. The treatment of low back pain in high-income countries is likely to be influenced by access to health care, governing payment models that include health insurance, and health promotion campaigns.

Our results show that the prevalence of low back pain increases with age, with a peak rate observed at approximately 85 years of age. It is known from previous research that, compared with younger adults, older adults are more likely to be severely incapacitated by low back pain, with loss of mobility and independence, leading to greater care needs.^25^ In fact, one-fifth of older adults with low back pain report difficulties in caring for themselves at home or participating in family and social activities.^26^ Older people are also more likely to report poorer outcomes and slower recovery^27^ when compared with younger adults. As the population ages, the inclusion of targeted and specific management recommendations for the older population, which take into consideration age-relevant clinical outcomes (ie, institutionalisation, falls, mobility), preferences, and acceptability is essential in decreasing the burden of low back pain globally. An important and global initiative, the WHO Integrated Care for Older People (ICOPE) approach, grounded on the healthy ageing model, highlights the need to increase intrinsic musculoskeletal health capacity in older people, offering guidance for the assessment and management of the older patient in primary care.^28^

Estimates show that among those available in the GBD study, three modifiable factors play an important role in the global burden of low back pain. Nearly one-quarter of YLDs due to low back pain were attributed to occupational ergonomic factors, which can include prolonged sitting or standing, bending, or lifting. As previously discussed, low back pain forces more people out of the workplace than any other chronic health condition.^21^ Work exposures to lifting, bending, awkward postures, vibration, and tasks considered physically demanding are associated with an increased risk of developing low back pain; however, independent causal relationships have not been demonstrated.^29^, ^30^ Globally, 12·5% and 11·5% of YLDs due to low back pain were attributed to the lifestyle factors smoking and elevated BMI, respectively. Although both smoking^31^ and obesity^32^ have been shown to be associated with the occurrence of low back pain and the development of persistent low back pain, the specific causal mechanisms for these associations remain uncertain. Likewise, we lack evidence on the effectiveness of preventive strategies targeting these two risk factors.

Strengths of this systematic analysis include adjustment of data to increase comparability between disparate sources, and capability of DisMod-MR 2.1 to leverage information from data-rich locations to inform estimates where no data are available. The current analyses also include, for the first time, prevalence projections to 2050 and the relative contribution of GBD risk factors to the burden of low back pain.

Limitations include the heterogeneous nature of input data, which reported low back pain based on a wide range of case definitions and recall periods. Although regression methods produce adjustments to make data sources with disparate case definitions more comparable, these methods additionally introduce uncertainty and rely on generalisation from a limited number of studies that provide data on different case definitions. This issue was compounded by relatively few predictive covariates in our models, as the relationship between low back pain prevalence and other potential risk factors is not well quantified. Lastly, our projections have not accounted for the impact of COVID-19 on the prevalence and burden of low back pain, including worsened occupational ergonomic factors, decreased access to treatment, or higher mortality in older adults.

Challenges persist in obtaining primary country-level data on low back pain prevalence, especially in low-income countries. This is mainly due to data sparsity, difficulties in collecting representative samples, and countries having restricted data-sharing policies. Data sparsity can increase the influence of granular high-income data (such as US claims data), which can unduly impact global age and time patterns. This lack of primary data limits the ability to draw strong inference from any regional and country-level variations. The Surveillance Task Force of the Global Alliance for Musculoskeletal Health has identified collections of national musculoskeletal burden data from low-income and middle-income countries as a priority and have developed and field-tested a musculoskeletal survey questionnaire.^33^ While our estimates benefited from the inclusion of multi-country surveys such as the World Health and COPCORD surveys, the collection of additional and standardised primary data in these regions remains a high priority. Finally, we acknowledge that our estimates for many countries are based on modelled rather than observed data.^34^ Although we recognise it would be ideal to have primary-level data from every country, and using standardised methodology, this is unlikely to be achieved. By using statistical adjustments for key covariates and harmonisation of between-study heterogeneity by formally using a reference definition, our modelled data provide the most accurate estimates of global prevalence and burden of low back pain.

In conclusion, in 2020, there were more than half a billion prevalent cases of low back pain worldwide, and by 2050 this is projected to increase to more than 800 million prevalent cases. Age-standardised rates have decreased slightly over the past three decades, but case numbers continue to rise because of population growth and ageing, particularly in Asia and Africa. Low back pain remains the leading cause of years lived with disability globally. Prevalence and years lived with disability due to low back pain increase with age, peaking at 85 years, and compromise the prospect of healthy ageing. Our results highlight the urgent need for more and high-quality primary country-level data on both prevalence and severity distributions to improve accuracy and monitor change as health policy and high-value care are implemented.

## Data sharing

Our study follows the Guidelines for Accurate and Transparent Health Estimates Reporting (GATHER). The findings of this study are supported by data available in public online repositories, data publicly available upon request of the data provider, and data not publicly available due to restrictions by the data provider. Non-publicly available data were used under license for the current study but may be available from the authors upon reasonable request and with permission of the data provider. Data sources used in this analysis are listed in the appendix (pp 53–80).

## Declaration of interests
