# Supplementary material for: Global, regional, and national burden of low back pain, 1990–2020, its attributable risk factors, and projections to 2050: a systematic analysis of the Global Burden of Disease Study 2021
Source: Lancet Rheumatol. 2023 May 22;5(6):e316–29. doi: 10.1016/S2665-9913(23)00098-X (PMC10234592; doi:10.1016/S2665-9913(23)00098-X)
Supplement: Supplementary appendix [file mmc1.pdf]

THE LANCET
Rheumatology

Supplementary appendix

This appendix formed part of the original submission and has been peer reviewed. We post it as supplied by the authors.

Supplement to: GBD 2021 Low Back Pain Collaborators. Global, regional, and national burden of low back pain, 1990–2020, its attributable risk factors, and projections to 2050: a systematic analysis of the Global Burden of Disease Study 2021. *Lancet Rheumatol* 2023; **5:** e316–29.

Appendix

This appendix provides supplemental figures and more detailed results. Portions of this appendix have been reproduced or adapted from Vos et al.[1] References are provided for reproduced sections.

Section 1. Statement of GATHER Compliance

This study complies with the Guidelines for Accurate and Transparent Health Estimates Reporting (GATHER) recommendations.[10]

Section 2. Data seeking

We conducted an updated systematic review of population-representative surveys reporting on low back pain prevalence from October 2016 to October 2017 by searching PUBMED, Ovid Medline, EMBase, and CINAHL databases using the terms “back pain”, “lumbar”, “back ache”, “backache”, and “lumbago” individually and in combination with “prevalence”, “incidence”, “cross-sectional”, and “epidemiology”, and again from September 2017 to October 2019 by searching PUBMED using the terms “back pain,” “prevalence,” and “incidence.” Sources that were not population-representative, had a sample size under 150, or did not include primary data were excluded. Additional studies between 2017 and 2019 were added via opportunistic review.

Section 3. Sex-splitting, crosswalking, and other data adjustment methods

If studies contained both sex-specific and separate age-specific estimates, the male-to-female ratio was used to proportionally split age-specific data and produce age- and sex-specific prevalence data. For studies with only "both"-sex data, studies with both male and female data were used to produce male/female ratios which were log-transformed and used as input data into a regression analysis using the MR-BRT (meta-regression—Bayesian, regularised, trimmed) tool. Model results were used to proportionally split "both"-sex data. Data with age ranges spanning more than 25 years were split into five-year age bins using age patterns derived from GBD 2019 final models for low back pain.

Studies that defined low back pain with recall periods of one week to one month, recall periods between two months and one year, or as activity-limiting LBP were adjusted for bias utilising MR-BRT. Beta coefficients and exponentiated values for these covariates are shown in the table below.

Section 4. Modelling strategy

Prior settings in the DisMod model included setting excess mortality to zero, and it was assumed that there was no incidence or prevalence of low back pain before the age of 5 years. We made no substantive changes in the modelling strategy from GBD 2017. We included the SEV scalar for low back pain as a country covariate. This combines the exposure measures for risks estimated to impinge on LBP in GBD: occupational ergonomic exposure and increased BMI. We set bounds of 0.75 to 1.25, as the SEV is constructed in a way that if our risk estimates are accurate the value should be 1.

Section 5. Disability weights

Disability weights (DWs) represent the severity of the disease and range from 0 (perfect health) to 1 (equivalent to death). DWs were derived from disability weight surveys (described elsewhere[33]), which used pair-wise comparison methods in which respondents were asked to indicate which of two health states briefly described to them they considered to be "healthier". A total of four surveys were conducted in LMICs, four internet surveys conducted in multiple European countries, one telephone survey in the USA, and one open-access online survey conducted globally to provide pair-wise comparisons. DWs were then derived from these comparisons and given numerical values. More detailed explanation of the disability weights used can be found elsewhere.[3,33] Disability weights for low back pain are shown in Supplemental Table 3.

Section 6. Severity distribution

The severity distributions are derived from an analysis of the Medical Expenditure Panel Surveys (MEPS) in the USA. MEPS is an overlapping continuous panel survey of the United States non-institutionalised population whose primary purpose is to collect information on the use and cost of health care (http://www.meps.ahrq.gov/survey_comp/hc_data_collection.jsp). MEPS was initiated in 1996 but only began collecting health status data in the form of SF-12 responses in 2000. For GBD 2016 we used data from 2000 to 2014. Respondents self-administer the SF-12 twice per panel, at rounds 2 and 4, typically about a year apart. Only adults 18 years and older completed the SF-12. MEPS also usually collects information on diagnoses based on self-report of reasons for encounters with health services. In addition, diagnoses are derived through additional questions on “problems that bother you” or conditions that led to “disability days,” ie, days out of role due to illness. Professional coders translate the verbatim text into three-digit ICD-9 codes. The main reason for LBP being measured in MEPS relates to health-care contact.

We used USA claims data (2012) to derive the proportion of cases with low back pain who report leg pain. The proportions were different by age group as shown in Figure 1. The proportion in each severity level in each age group is calculated by multiplying the proportion in the severity level and the proportion with or without leg pain.

2020 Data Input Sources.

Badley EM, Millstone DB, Perruccio AV. Back Pain and Co-occurring Conditions: Findings From a Nationally Representative Sample. Spine. 2018; 43(16): E935-E941.

Galozzi P, Maghini I, Bakdounes L, Ferlito E, Lazzari V, Ermani M, Chia M, Gatti D, Masiero S, Punzi L. Prevalence of low back pain and its effect on health-related quality of life in 409 scholar adolescents from the Veneto region. Reumatismo. 2019 Oct 24;71(3):132-40.

Guevara-Pacheco SV, Feican-Alvarado A, Delgado-Pauta J, Lliguisaca-Segarra A, Pelaez-Ballestas I. Prevalence of Disability in Patients With Musculoskeletal Pain and Rheumatic Diseases in a Population From Cuenca, Ecuador. J Clin Rheumatol. 2017; 23(6): 324–9.

Akbar F, AlBesharah M, Al-Baghli J, Bulbul F, Mohammad D, Qadoura B, Al-Taiar A. Prevalence of low Back pain among adolescents in relation to the weight of school bags. BMC Musculoskelet Disord. 2019; 20(1): 37.

Ben Ayed H, Yaich S, Trigui M, Ben Hmida M, Ben Jemaa M, Ammar A, Jedidi J, Karray R, Feki H, Mejdoub Y, Kassis M, Damak J. Prevalence, Risk Factors and Outcomes of Neck, Shoulders and Low-Back Pain in Secondary-School Children. J Res Health Sci. 2019; 19(1): e00440.

Borgman S, Ericsson I, Clausson EK, Garmy P. The Relationship Between Reported Pain and Depressive Symptoms Among Adolescents. J Sch Nurs. 2020; 36(2): 87-93.

Gonçalves TR, Mediano MFF, Sichieri R, Cunha DB. Is Health-related Quality of Life Decreased in Adolescents With Back Pain?. Spine. 2018; 43(14): E822-E829.

Horii C, Asai Y, Iidaka T, Muraki S, Oka H, Tsutsui S, Hashizume H, Yamada H, Yoshida M, Kawaguchi H, Nakamura K, Akune T, Tanaka S, Yoshimura N. Differences in prevalence and associated factors between mild and severe vertebral fractures in Japanese men and women: the third survey of the ROAD study. J Bone Miner Metab. 2019; 37(5): 844-853.

Laslett LL, Menz HB, Otahal P, Pan F, Cicuttini FM, Jones G. Factors associated with prevalent and incident foot pain: data from the Tasmanian Older Adult Cohort Study. Maturitas. 2018; 118: 38-43.

Muntaner-Mas A, Palou P, Ortega FB, Vidal-Conti J. Sports participation and low back pain in schoolchildren. J Back Musculoskelet Rehabil. 2018; 31(5): 811-819.

Noormohammadpour P, Borghei A, Mirzaei S, Mansournia MA, Ghayour-Najafabadi M, Kordi M, Kordi R. The Risk Factors of Low Back Pain in Female High School Students. Spine. 2019; 44(6): E357-E365.

Oka GA, Ranade AS, Kulkarni AA. Back pain and school bag weight - a study on Indian children and review of literature. J Pediatr Orthop B. 2019; 28(4): 397-404.

Scarabottolo CC, Pinto RZ, Oliveira CB, Zanuto EF, Cardoso JR, Christofaro DGD. Back and neck pain prevalence and their association with physical inactivity domains in adolescents. Eur Spine J. 2017; 26(9): 2274-2280.

Schauer B, Grabe HJ, Ittermann T, Lerch MM, Weiss FU, Mönnikes H, Völzke H, Enck P, Schwille-Kiuntke J. Irritable bowel syndrome, mental health, and quality of life: Data from a population-based survey in Germany (SHIP-Trend-0). Neurogastroenterol Motil. 2019; 31(3): e13511.

Sundell CG, Bergström E, Larsén K. Low back pain and associated disability in Swedish adolescents. Scand J Med Sci Sports. 2019; 29(3): 393-399.

Ho KKN, Simic M, Cvancarova Småstuen M, de Barros Pinheiro M, Ferreira PH, Bakke Johnsen M, Heuch I, Grotle M, Zwart JA, Nilsen KB. The association between insomnia, c-reactive protein, and chronic low back pain: cross-sectional analysis of the HUNT study, Norway. Scand J Pain. 2019; 19(4): 765-77.

Keeratisiroj O, Siritaratiwat W. Prevalence of self-reported musculoskeletal pain symptoms among school-age adolescents: age and sex differences. Scand J Pain. 2018; 18(2): 273-80.

Stockil L, Thompson J, Briffa K, Smith A, Beales D, Straker L, O'Sullivan P, Jacques A. Urogenital symptoms: prevalence, bother, associations and impact in 22year-old women of the Raine Study. Int Urogynecol J. 2018; 29(12): 1807-1815.

Wang XD, Ma L, Wang DH, Yan JT. Relationships among the lumbar lordosis index, sacral horizontal angle, and chronic low back pain in the elderly aged 60-69 years: A cross-sectional study. J Back Musculoskelet Rehabil. 2020; 33(1): 29-33.

Bedene A, Lijfering WM, Niesters M, van Velzen M, Rosendaal FR, Bouvy ML, Dahan A, van Dorp ELA. Opioid Prescription Patterns and Risk Factors Associated With Opioid Use in the Netherlands. JAMA Netw Open. 2019; 2(8): e1910223.

El-Metwally A, Shaikh Q, Aldiab A, Al-Zahrani J, Al-Ghamdi S, Alrasheed AA, Househ M, Da'ar OB, Nooruddin S, Razzak HA, Aldossari KK. The prevalence of chronic pain and its associated factors among Saudi Al-Kharj population; a cross sectional study. BMC Musculoskelet Disord. 2019; 20(1): 177.

Endo T, Abe T, Akai K, Kijima T, Takeda M, Yamasaki M, Isomura M, Nabika T, Yano S. Height loss but not body composition is related to low back pain in community-dwelling elderlies: Shimane CoHRE study. BMC Musculoskelet Disord. 2019; 20(1): 207.

Fink HA, Litwack-Harrison S, Ensrud KE, Shen J, Schousboe JT, Cawthon PM, Cauley JA, Lane NE, Taylor BC, Barrett-Connor E, Kado DM, Cummings SR, Marshall LM, Osteoporotic Fractures in Men (MrOS) Study Group. Association of Incident, Clinically Undiagnosed Radiographic Vertebral Fractures With Follow-Up Back Pain Symptoms in Older Men: the Osteoporotic Fractures in Men (MrOS) Study. J Bone Miner Res. 2017; 32(11): 2263-2268.

Iizuka Y, Iizuka H, Mieda T, Tsunoda D, Sasaki T, Tajika T, Yamamoto A, Takagishi K. Prevalence of Chronic Nonspecific Low Back Pain and Its Associated Factors among Middle-Aged and Elderly People: An Analysis Based on Data from a Musculoskeletal Examination in Japan. Asian Spine J. 2017; 11(6): 989-97.

Ikeda T, Sugiyama K, Aida J, Tsuboya T, Watabiki N, Kondo K, Osaka K. Socioeconomic inequalities in low back pain among older people: the JAGES cross-sectional study. Int J Equity Health. 2019; 18(1): 15.

Joergensen AC, Hestbaek L, Andersen PK, Nybo Andersen AM. Epidemiology of spinal pain in children: a study within the Danish National Birth Cohort. Eur J Pediatr. 2019; 178(5): 695-706.

Kikuchi R, Hirano T, Watanabe K, Sano A, Sato T, Ito T, Endo N, Tanabe N. Gender differences in the prevalence of low back pain associated with sports activities in children and adolescents: a six-year annual survey of a birth cohort in Niigata City, Japan. BMC Musculoskelet Disord. 2019; 20(1): 327.

Kędra A, Kolwicz-Gańko A, Sitarski D, Kędra P, Czaprowski D. Prevalence of back pain and the knowledge of preventive measures in a cohort of 11619 Polish school-age children and youth-an epidemiological study. Medicine (Baltimore). 2019; 98(22): e15729.

Paranjape S, Ingole V. Prevalence of Back Pain in Secondary School Students in an Urban Population: Cross-sectional Study. Cureus. 2018; 10(7): e2983.

Puth MT, Klaschik M, Schmid M, Weckbecker K, Münster E. Prevalence and comorbidity of osteoporosis- a cross-sectional analysis on 10,660 adults aged 50years and older in Germany. BMC Musculoskelet Disord. 2018; 19(1): 144.

Sasaki T, Yoshimura N, Hashizume H, Yamada H, Oka H, Matsudaira K, Iwahashi H, Shinto K, Ishimoto Y, Nagata K, Teraguchi M, Kagotani R, Muraki S, Akune T, Tanaka S, Kawaguchi H, Nakamura K, Minamide A, Nakagawa Y, Yoshida M. MRI-defined paraspinal muscle morphology in Japanese population: The Wakayama Spine Study. PLoS One. 2017; 12(11): e0187765.

Takahashi A, Kitamura K, Watanabe Y, Kobayashi R, Saito T, Takachi R, Kabasawa K, Oshiki R, Tsugane S, Iki M, Sasaki A, Yamazaki O, Nakamura K. Epidemiological profiles of chronic low back and knee pain in middle-aged and elderly Japanese from the Murakami cohort. J Pain Res. 2018; 11: 3161-3169.

Saraiva BTC, Pinto RZ, Oliveira CB, Zanuto EF, Scarabottolo CC, Delfino LD, Suetake VYB, Gil FCS, Christofaro DGD. Continuity of physical activity practice from childhood to adolescence is associated with lower neck pain in both sexes and lower back pain in girls. J Back Musculoskelet Rehabil. 2020; 33(2): 269-275.

Bento TPF, Genebra CVDS, Maciel NM, Cornelio GP, Simeão SFAP, Vitta A. Low back pain and some associated factors: is there any difference between genders?. Braz J Phys Ther. 2020; 24(1): 79-87.

Heuch I, Heuch I, Hagen K, Sørgjerd EP, Åsvold BO, Zwart JA. Is chronic low back pain a risk factor for diabetes? The Nord-Trøndelag Health Study. BMJ Open Diabetes Res Care. 2018; 6(1): e000569.

Schwertner DS, Oliveira RANS, Koerich MHAL, Motta AF, Pimenta AL, Gioda FR. Prevalence of low back pain in young Brazilians and associated factors: Sex, physical activity, sedentary behavior, sleep and body mass index. J Back Musculoskelet Rehabil. 2020; 33(2): 233-244.

GBD 2021 Low Back Pain Collaborators and affiliations

Manuela L Ferreira, Katie de Luca, Lydia M Haile, Jaimie D Steinmetz, Garland T Culbreth, Marita Cross, Jacek A Kopec, Paulo H Ferreira, Fiona M Blyth, Rachelle Buchbinder, Jan Hartvigsen, Ai-Min Wu, Saeid Safiri, Anthony D Woolf, Gary S Collins, Kanyin Liane Ong, Stein Emil Vollset, Amanda E Smith, Jessica A Cruz, Kai Glenn Fukutaki, Semagn Mekonnen Abate, Mitra Abbasifard, Mohsen Abbasi-Kangevari, Zeinab Abbasi-Kangevari, Ahmed Abdelalim, Aidin Abedi, Hassan Abidi, Qorinah Estiningtyas Sakilah Adnani, Ali Ahmadi, Rufus Olusola Akinyemi, Abayneh Tadesse Alamer, Adugnaw Zeleke Alem, Yousef Alimohamadi, Mansour Abdullah Alshehri, Mohammed Mansour Alshehri, Hosam Alzahrani, Saeed Amini, Sohrab Amiri, Hubert Amu, Catalina Liliana Andrei, Tudorel Andrei, Benny Antony, Jalal Arabloo, Judie Arulappan, Ashokan Arumugam, Tahira Ashraf, Seyyed Shamsadin Athari, Nefsu Awoke, Sina Azadnajafabad, Till Winfried Bärnighausen, Lope H Barrero, Amadou Barrow, Akbar Barzegar, Lindsay M Bearne, Isabela M Bensenor, Alemshet Yirga Berhie, Bharti Bhandari Bhandari, Vijayalakshmi S Bhojaraja, Ali Bijani, Belay Boda Abule Bodicha, Srinivasa Rao Bolla, Javier Brazo-Sayavera, Andrew M Briggs, Chao Cao, Periklis Charalampous, Vijay Kumar Chattu, Flavia M Cicuttini, Benjamin Clarsen, Sarah Cuschieri, Omid Dadras, Xiaochen Dai, Lalit Dandona, Rakhi Dandona, Azizallah Dehghan, Takele Gezahegn G Demie, Edgar Denova-Gutiérrez, Syed Masudur Rahman Dewan, Samath Dhamminda Dharmaratne, Mandira Lamichhane Dhimal, Meghnath Dhimal, Daniel Diaz, Mojtaba Didehdar, Lankamo Ena Digesa, Mengistie Diress, Hoa Thi Do, Linh Phuong Doan, Michael Ekholuenetale, Muhammed Elhadi, Sharareh Eskandarieh, Shahriar Faghani, Jawad Fares, Ali Fatehizadeh, Getahun Fetensa, Irina Filip, Florian Fischer, Richard Charles Franklin, Balasankar Ganesan, Belete Negese Belete Gemeda, Motuma Erena Getachew, Ahmad Ghashghaee, Tiffany K Gill, Mahaveer Golechha, Pouya Goleij, Bhawna Gupta, Nima Hafezi-Nejad, Arvin Haj-Mirzaian, Pawan Kumar Hamal, Asif Hanif, Netanja I Harlianto, Hamidreza Hasani, Simon I Hay, Jeffrey J Hebert, Golnaz Heidari, Mohammad Heidari, Reza Heidari-Soureshjani, Mbuzeleni Mbuzeleni Hlongwa, Mohammad-Salar Hosseini, Alexander Kevin Hsiao, Ivo Iavicoli, Segun Emmanuel Ibitoye, Irena M Ilic, Milena D Ilic, Sheikh Mohammed Shariful Islam, Manthan Dilipkumar Janodia, Ravi Prakash Jha, Har Ashish Jindal, Jost B Jonas, Gebisa Guyasa Kabito, Himal Kandel, Rimple Jeet Kaur, Vikash Ranjan Keshri, Yousef Saleh Khader, Ejaz Ahmad Khan, Md Jobair Khan, Moien AB Khan, Hamid Reza Khayat Kashani, Jagdish Khubchandani, Yun Jin Kim, Adnan Kisa, Jitka Klugarová, Ali-Asghar Kolahi, Hamid Reza Koohestani, Ai

Koyanagi, G Anil Kumar, Narinder Kumar, Tea Lallukka, Savita Lasrado, Wei-Chen Lee, Yo Han Lee, Ata Mahmoodpoor, Jeadran N Malagón-Rojas, Mohammad-Reza Malekpour, Reza Malekzadeh, Narges Malih, Man Mohan Mehndiratta, Entezar Mehrabi Nasab, Ritesh G Menezes, Alexios-Fotios A Mentis, Mohamed Kamal Mesregah, Ted R Miller, Mohammad Mirza-Aghazadeh-Attari, Maryam Mobarakabadi, Yousef Mohammad, Esmaeil Mohammadi, Shafiu Mohammed, Ali H Mokdad, Sara Momtazmanesh, Lorenzo Monasta, Mohammad Ali Moni, Ebrahim Mostafavi, Christopher J L Murray, Tapas Sadasivan Nair, Javad Nazari, Seyed Aria Nejadghaderi, Subas Neupane, Sandhya Neupane Kandel, Cuong Tat Nguyen, Ali Nowroozi, Hassan Okati-Aliabad, Emad Omer, Abderrahim Oulhaj, Mayowa O Owolabi, Songhomitra Panda-Jonas, Anamika Pandey, Eun-Kee Park, Shrikant Pawar, Paolo Pedersini, Jeevan Pereira, Mario F P Peres, Ionela-Roxana Petcu, Mohammadreza Pourahmadi, Amir Radfar, Shahram Rahimi-Dehgolan, Vafa Rahimi-Movaghar, Mosiur Rahman, Amir Masoud Rahmani, Nazanin Rajai, Chythra R Rao, Vahid Rashedi, Mohammad-Mahdi Rashidi, Zubair Ahmed Ratan, David Laith Rawaf, Salman Rawaf, Andre M N Renzaho, Negar Rezaei, Zahed Rezaei, Leonardo Roever, Guilherme de Andrade Ruela, Basema Saddik, Amirhossein Sahebkar, Sana Salehi, Francesco Sanmarchi, Sadaf G Sepanlou, Saeed Shahabi, Shayan Shahrokhi, Elaheh Shaker, MohammadBagher Shamsi, Mohammed Shannawaz, Saurab Sharma, Maryam Shaygan, Rahim Ali Sheikhi, Jeevan K Shetty, Rahman Shiri, Siddharudha Shivalli, Parnian Shobeiri, Migbar Mekonnen Sibhat, Ambrish Singh, Jasvinder A Singh, Helen Slater, Marco Solmi, Ranjani Somayaji, Ker-Kan Tan, Rekha Thapar, Seyed Abolfazl Tohidast, Sahel Valadan Tahbaz, Rohollah Valizadeh, Tommi Juhani Vasankari, Narayanaswamy Venketasubramanian, Vasily Vlassov, Bay Vo, Yuan-Pang Wang, Taweewat Wiangkham, Lalit Yadav, Ali Yadollahpour, Seyed Hossein Yahyazadeh Jabbari, Lin Yang, Fereshteh Yazdanpanah, Naohiro Yonemoto, Mustafa Z Younis, Iman Zare, Armin Zarrintan, Mohammad Zoladl, Theo Vos*, and Lyn M March*.

*Jointly supervised the work

Affiliations

Institute of Bone and Joint Research (Prof M L Ferreira PhD, L M March PhD), Faculty of Medicine and Health (M Cross PhD, Prof P H Ferreira PhD), School of Public Health (Prof F M Blyth PhD), Sydney Medical School (S Islam PhD), Save Sight Institute (H Kandel PhD), University of Sydney, Sydney, NSW, Australia; Discipline of Chiropractic (K de Luca PhD),

CQ University, Brisbane, QLD, Australia; Institute for Health Metrics and Evaluation (L M Haile MPH, J D Steinmetz PhD, G T Culbreth PhD, K L Ong PhD, Prof S Vollset DrPH, A E Smith MPA, J A Cruz BSc, X Dai PhD, Prof L Dandona MD, Prof R Dandona PhD, Prof S D Dharmaratne MD, Prof S I Hay FMedSci, A H Mokdad PhD, Prof C J L Murray DPhil, Prof T Vos PhD), Department of Health Metrics Sciences, School of Medicine (Prof S Vollset DrPH, X Dai PhD, Prof R Dandona PhD, Prof S D Dharmaratne MD, Prof S I Hay FMedSci, A H Mokdad PhD, Prof C J L Murray DPhil, Prof T Vos PhD), Department of Medicine (R Somayaji MD), University of Washington, Seattle, WA, USA; School of Population and Public Health (J A Kopec PhD), University of British Columbia, Vancouver, BC, Canada; Arthritis Research Canada, Richmond, BC, Canada (J A Kopec PhD); Department of Epidemiology and Preventive Medicine (Prof R Buchbinder PhD), School of Public Health and Preventive Medicine (Prof F M Cicuttini PhD), Monash University, Melbourne, VIC, Australia; Monash Department of Clinical Epidemiology at Cabrini Hospital (Prof R Buchbinder PhD), Cabrini Institute, Melbourne, VIC, Australia; Department of Sports Science and Clinical Biomechanics (Prof J Hartvigsen PhD), University of Southern Denmark, Odense, Denmark; Research Department (Prof J Hartvigsen PhD), Nordic Institute of Chiropractic and Clinical Biomechanics, Odense, Denmark; Department of Orthopaedics (Prof A Wu MD), Wenzhou Medical University, Wenzhou, China; Aging Research Institute (S Safiri PhD), Department of Community Medicine (S Safiri PhD), Student Research Committee (M Hosseini MD), Anesthesiology and Critical Care (Prof A Mahmoodpoor MD), Department of Radiology (M Mirza-Aghazadeh-Attari MD, A Zarrintan MD), Department of Pediatric Allergy and Immunology (F Yazdanpanah MD), Tabriz University of Medical Sciences, Tabriz, Iran; Bone and Joint Research Group (Prof A D Woolf MBBS), Royal Cornwall Hospital, Truro, UK; Nuffield Department of Orthopaedics, Rheumatology, and Musculoskeletal Sciences (Prof G S Collins PhD), University of Oxford, Oxford, UK; Public Health (K G Fukutaki MPH), Independent Consultant, Seattle, WA, USA; Anesthesiology Department (S M Abate MSc), Dilla University, Addis Ababa, Ethiopia; Department of Internal Medicine (M Abbasifard MD), Clinical Research Development Unit (M Abbasifard MD), Rafsanjan University of Medical Sciences, Rafsanjan, Iran; Non-communicable Diseases Research Center (M Abbasi-Kangevari MD, Z Abbasi-Kangevari BSc, S Azadnajafabad MD, M Malekpour MD, S Momtazmanesh MD, M Rashidi MD, N Rezaei PhD), Multiple Sclerosis Research Center (S Eskandarieh PhD), Interdisciplinary Neuroscience Research Program (S Faghani MD), Faculty of Medicine (N Hafezi-Nejad MD, E Mohammadi MD, E Shaker MD, P Shobeiri MD), Digestive Diseases Research Institute

(Prof R Malekzadeh MD, S G Sepanlou MD), Tehran Heart Center (E Mehrabi Nasab MD), School of Medicine (S Momtazmanesh MD, A Nowroozi BMedSc), Physical Medicine and Rehabilitation (S Rahimi-Dehgolan MD), Sina Trauma and Surgery Research Center (Prof V Rahimi-Movaghar MD), Endocrinology and Metabolism Research Institute (N Rezaei PhD), Department of Psychiatry (S Shahrokhi MD), Department of Pediatric Allergy and Immunology (F Yazdanpanah MD), Tehran University of Medical Sciences, Tehran, Iran (R Heidari-Soureshjani MSc, E Mohammadi MD); Social Determinants of Health Research Center (Z Abbasi-Kangevari BSc, A Kolahi MD, M Rashidi MD), Department of Epidemiology (A Ahmadi PhD), Department of Pharmacology (A Haj-Mirzaian MD), Obesity Research Center (A Haj-Mirzaian MD), Department of Neurosurgery (H Khayat Kashani MD), School of Medicine (S Nejadghaderi MD), Shahid Beheshti University of Medical Sciences, Tehran, Iran; Department of Neurology (Prof A Abdelalim MD), Cairo University, Cairo, Egypt; Department of Neurosurgery (A Abedi MD), Keck School of Medicine (A Abedi MD), Mark and Mary Stevens Neuroimaging and Informatics Institute (S Salehi MD), University of Southern California, Los Angeles, CA, USA; Laboratory Technology Sciences Department (H Abidi PhD), Department of Nursing (M Zoladl PhD), Yasuj University of Medical Sciences, Yasuj, Iran; Faculty of Medicine (Q E S Adnani PhD), Universitas Padjadjaran (Padjadjaran University), Bandung, Indonesia; Department of Epidemiology and Biostatistics (A Ahmadi PhD), Community-Oriented Nursing Midwifery Research Center (M Heidari PhD), Department of Health in Disasters and Emergencies (R Sheikhi BHlthSci), Shahrekord University of Medical Sciences, Shahrekord, Iran; Institute for Advanced Medical Research and Training (R O Akinyemi PhD), Department of Epidemiology and Medical Statistics (M Ekholuenetale MSc), Faculty of Public Health (M Ekholuenetale MSc), Department of Health Promotion and Education (S E Ibitoye MPH), Department of Medicine (Prof M O Owolabi DrM), University of Ibadan, Ibadan, Nigeria; Institute of Neuroscience (R O Akinyemi PhD), Newcastle University, Newcastle upon Tyne, UK; Department of Physiotherapy (A T Alamer MSc), Mekelle University, Mekelle, Ethiopia; Department of Epidemiology and Biostatistics (A Z Alem MPH), Department of Human Physiology (M Diress MSc), Environmental and Occupational Health and Safety Department (G G Kabito MPH), University of Gondar, Gondar, Ethiopia; Health Research Center (Y Alimohamadi PhD), Quran and Hadith Research Center (S Amiri PhD), Baqiyatallah University of Medical Sciences, Tehran, Iran; Physiotherapy Department (M A Alshehri PhD), Umm Al-Qura University, Mecca, Saudi Arabia; Pharmaceutical Care (M Alshehri PharmD), Ministry of National Guard-Health Affairs, Riyadh, Saudi Arabia; Taif

University, Taif, Saudi Arabia (H Alzahrani PhD); Department of Health Services Management (S Amini PhD), Khomein University of Medical Sciences, Khomein, Iran; Department of Population and Behavioural Sciences (H Amu PhD), University of Health and Allied Sciences, Ho, Ghana; Cardiology Department (C Andrei PhD), Carol Davila University of Medicine and Pharmacy, Bucharest, Romania; Department of Statistics and Econometrics (Prof T Andrei PhD, I Petcu PhD), Bucharest University of Economic Studies, Bucharest, Romania; Menzies Institute for Medical Research (B Antony PhD, A Singh Mtech), University of Tasmania, Hobart, TAS, Australia; Health Management and Economics Research Center (J Arabloo PhD), Department of Physiotherapy (M Pourahmadi PhD), Iran University of Medical Sciences, Tehran, Iran; Department of Maternal and Child Health (J Arulappan DSc), Sultan Qaboos University, Muscat, Oman; Department of Physiotherapy (A Arumugam PhD), Sharjah Institute for Medical Research (B Saddik PhD), University of Sharjah, Sharjah, United Arab Emirates; Community Medicine and Rehabilitation - Physiotherapy Section (A Arumugam PhD), Umeå University, Umea, Sweden; University Institute of Radiological Sciences and Medical Imaging Technology (T Ashraf MS), University Institute of Public Health (A Hanif PhD), The University of Lahore, Lahore, Pakistan; Department of Immunology (S Athari PhD), Zanjan University of Medical Sciences, Zanjan, Iran; Department of Nursing (N Awoke MSc), Wolaita Sodo University, Wolaita Sodo, Ethiopia; Heidelberg Institute of Global Health (HIGH) (Prof T W Bärnighausen MD), Heidelberg University, Heidelberg, Germany; TH Chan School of Public Health (Prof T W Bärnighausen MD), Department of Internal Medicine (N Rajai MD), Harvard University, Boston, MA, USA; Department of Industrial Engineering (Prof L H Barrero DSc), Pontifical Javeriana University, Bogota, Colombia; Department of Public & Environmental Health (A Barrow MPH), University of The Gambia, Brikama, The Gambia; Epidemiology and Disease Control Unit (A Barrow MPH), Ministry of Health, Kotu, The Gambia; Department of Occupational Health Engineering (A Barzegar PhD), School of Public Health (Z Rezaei PhD), Department of Sports Medicine and Rehabilitation (M Shamsi PhD), Kermanshah University of Medical Sciences, Kermanshah, Iran; Population Health Research Institute (Prof L M Bearne PhD), University of London, London, UK; Centre for Engagement and Dissemination (Prof L M Bearne PhD), National Institute for Health Research, Twickenham, UK; Department of Internal Medicine (I M Bensenor PhD), Department of Psychiatry (Prof M F P Peres MD, Y Wang PhD), University of São Paulo, São Paulo, Brazil; School of Health Science (A Y Berhie MSc), Bahir Dar University, Bahir Dar, Ethiopia; Physiology Department (B B Bhandari MD), Government Institute of Medical

Sciences, Greater Noida, India; Department of Anatomy (V S Bhojaraja MD), Department of Biochemistry (J K Shetty MD), Royal College of Surgeons in Ireland Medical University of Bahrain, Busaiteen, Bahrain; Social Determinants of Health Research Center (A Bijani PhD), Babol University of Medical Sciences, Babol, Iran; Biomedical Sciences Department (B B A Bodicha MSc), Department of Comprehensive Nursing (L E Digesa MSc), Arba Minch University, Arba Minch, Ethiopia; Department of Biomedical Sciences (S Bolla PhD), Nazarbayev University, Nur-Sultan City, Kazakhstan; Centro Regional Universitario Noreste (J Brazo-Sayavera PhD), University of the Republic of Uruguay, Rivera, Uruguay; School of Physiotherapy and Exercise Science (Prof A M Briggs PhD, Prof H Slater PhD), School of Public Health (T R Miller PhD), Curtin University, Perth, WA, Australia; Program in Physical Therapy (C Cao MPH), Washington University in St Louis, St Louis, MO, USA; Department of Public Health (P Charalampous MSc), Erasmus University Medical Center, Rotterdam, Netherlands; Department of Community Medicine (V Chattu MD), Datta Meghe Institute of Medical Sciences, Sawangi, India; Saveetha Medical College and Hospitals (V Chattu MD), Saveetha University, Chennai, India; Department of Disease Burden (B Clarsen PhD), Norwegian Institute of Public Health, Bergen, Norway; Department of Sports Medicine (B Clarsen PhD), Norwegian School of Sport Sciences, Oslo, Norway; Anatomy Department (S Cuschieri PhD), University of Malta, Msida, Malta; Section Global Health and Rehabilitation (O Dadras DrPH), Western Norway University of Applied Sciences, Bergen, Norway; Department of Global Public Health and Primary Care (O Dadras DrPH), University of Bergen, Bergen, Norway; Department of Research (A Pandey PhD), Public Health Foundation of India, Gurugram, India (Prof L Dandona MD, Prof R Dandona PhD, G Kumar PhD); Indian Council of Medical Research, New Delhi, India (Prof L Dandona MD); Department of Epidemiology and Community Medicine (A Dehghan PhD), Non-Communicable Diseases Research Center (NCDRC), Fasa, Iran; Public Health Department (T G Demie MPH), St Paul's Hospital Millennium Medical College, Addis Ababa, Ethiopia; Emergency Operating Center (EOC) (T G Demie MPH), Ethiopian Public Health Institute, Addis Ababa, Ethiopia; Center for Nutrition and Health Research (E Denova-Gutiérrez DSc), National Institute of Public Health, Cuernavaca, Mexico; Department of Pharmacy (S Dewan PhD), University of Asia Pacific, Dhaka, Bangladesh; Pharmacology Department (S Dewan PhD), Center for Life Sciences Research Bangladesh, Dhaka, Bangladesh; Department of Community Medicine (Prof S D Dharmaratne MD), University of Peradeniya, Peradeniya, Sri Lanka; Policy Research Institute, Kathmandu, Nepal (M L Dhimal PhD); Global Institute for Interdisciplinary Studies, Kathmandu, Nepal (M L Dhimal PhD); Health Research Section

(M Dhimal PhD), Journal of Nepal Health Research Council (P K Hamal MD), Nepal Health Research Council, Kathmandu, Nepal; Center of Complexity Sciences (Prof D Diaz PhD), National Autonomous University of Mexico, Mexico City, Mexico; Faculty of Veterinary Medicine and Zootechnics (Prof D Diaz PhD), Autonomous University of Sinaloa, Culiacán Rosales, Mexico; Department of Parasitology and Mycology (M Didehdar PhD), Department of Pediatrics (J Nazari MD), Arak University of Medical Sciences, Arak, Iran; Institute of Health Economics and Technology, Hanoi, Viet Nam (H T Do MD); Institute for Global Health Innovations (L P Doan MSc), Faculty of Medicine (L P Doan MSc), Duy Tan University, Da Nang, Viet Nam; Faculty of Medicine (M Elhadi MD), University of Tripoli, Tripoli, Libya; Department of Neurological Surgery (J Fares MD), Northwestern University, Chicago, IL, USA; Department of Environmental Health Engineering (A Fatehizadeh PhD), Isfahan University of Medical Sciences, Isfahan, Iran; Department of Nursing (G Fetensa MSc), Department of Public Health (M E Getachew MPH), Wollega University, Nekemte, Ethiopia; Psychiatry Department (I Filip MD), Kaiser Permanente, Fontana, CA, USA; School of Health Sciences (I Filip MD), AT Still University, Mesa, AZ, USA; Institute of Public Health (F Fischer PhD), Charité Universitätsmedizin Berlin (Charité Medical University Berlin), Berlin, Germany; School of Public Health, Medical, and Veterinary Sciences (R C Franklin PhD), James Cook University, Douglas, QLD, Australia; School of Global Health (B Ganesan PhD), Institute of Health & Management, Melbourne, VIC, Australia; Department of Occupational Therapy (B Ganesan PhD), Mahatma Gandhi Occupational Therapy College, Jaipur, India; Department of Nursing (B N B Gemeda MSc), Debre Berhan University, Debre Birhan, Ethiopia; Department of Public Health (M E Getachew MPH), Jimma University, Jimma, Ethiopia; School of Public Health (A Ghashghaee BSc), Qazvin University of Medical Sciences, Qazvin, Iran; Adelaide Medical School (T K Gill PhD), Faculty of Health and Medical Sciences (J Klugarová PhD), University of Adelaide, Adelaide, SA, Australia; Health Systems and Policy Research (M Golechha PhD), Indian Institute of Public Health, Gandhinagar, India; Department of Genetics (P Goleij MSc), Sana Institute of Higher Education, Sari, Iran; Department of Public Health (B Gupta PhD), Torrens University Australia, Melbourne, VIC, Australia; Department of Radiology and Radiological Science (N Hafezi-Nejad MD), Johns Hopkins University, Baltimore, MD, USA; Department of Anaesthesiology and Intensive care (P K Hamal MD), National Academy of Medical Sciences, Kathmandu, Nepal; Faculty of Medicine (N I Harlianto BSc), Utrecht University, Utrecht, Netherlands; Department of Radiology (N I Harlianto BSc), University Medical Center Utrecht, Utrecht, Netherlands;

Department of Ophthalmology (H Hasani MD), Iran University of Medical Sciences, Karaj, Iran; Faculty of Kinesiology (Prof J J Hebert PhD), University of New Brunswick, Fredericton, NB, Canada; School of Psychology and Exercise Science (Prof J J Hebert PhD), Murdoch University, Murdoch, WA, Australia; Independent Consultant, Santa Clara, CA, USA (G Heidari MD); School of Nursing and Public Health Medicine (M M Hlongwa PhD), University of KwaZulu-Natal, Durban, South Africa; Outpatient Rehabilitation (A K Hsiao DPT), Southcoast Health: Tobey Hospital, Wareham, MA, USA; Department of Public Health (Prof I Iavicoli PhD), University of Naples Federico II, Naples, Italy; Faculty of Medicine (I M Ilic PhD), University of Belgrade, Belgrade, Serbia; Department of Epidemiology (Prof M D Ilic PhD), University of Kragujevac, Kragujevac, Serbia; Institute for Physical Activity and Nutrition (S Islam PhD), Deakin University, Burwood, VIC, Australia; Manipal College of Pharmaceutical Sciences (Prof M D Janodia PhD), Department of Community Medicine (C R Rao MD), Manipal Academy of Higher Education, Manipal, India; Department of Community Medicine (R P Jha MSc), Dr Baba Saheb Ambedkar Medical College & Hospital, Delhi, India; Department of Community Medicine (R P Jha MSc), Banaras Hindu University, Varanasi, India; National Health System Resource Centre (H Jindal MD), Ministry of Health & Family Welfare, New Delhi, India; Institute of Molecular and Clinical Ophthalmology Basel, Basel, Switzerland (Prof J B Jonas MD); Department of Ophthalmology (Prof J B Jonas MD), Heidelberg University, Mannheim, Germany; Sydney Eye Hospital (H Kandel PhD), South Eastern Sydney Local Health District, Sydney, NSW, Australia; Department of Pharmacology (R J Kaur PhD), All India Institute of Medical Sciences, Jodhpur, India; Injury Division (V R Keshri MD), Res, earch and Development Division (L Yadav PhD)The George Institute for Global Health, India, New Delhi, India; The George Institute for Global Health, Faculty of Medicine and Health (V R Keshri MD, S Sharma PhD), University of New South Wales, Sydney, NSW, Australia; Department of Public Health (Prof Y S Khader PhD), Jordan University of Science and Technology, Irbid, Jordan; Department of Epidemiology and Biostatistics (E A Khan MPH), Health Services Academy, Islamabad, Pakistan; Department of Rehabilitation Sciences (M Khan MPH), Hong Kong Polytechnic University, Hong Kong, China; Family Medicine Department (M A Khan MSc), United Arab Emirates University, Al Ain, United Arab Emirates; Primary Care Department (M A Khan MSc), NHS North West London, London, UK; Department of Public Health (Prof J Khubchandani PhD), New Mexico State University, Las Cruces, NM, USA; School of Traditional Chinese Medicine (Y Kim PhD), Xiamen University Malaysia, Sepang, Malaysia; School of Health Sciences (Prof A Kisa PhD),

Kristiania University College, Oslo, Norway; Department of International Health and Sustainable Development (Prof A Kisa PhD), Tulane University, New Orleans, LA, USA; Czech National Centre for Evidence-Based Healthcare and Knowledge Translation (J Klugarová PhD), Masaryk University, Brno, Czech Republic; Social Determinants of Health Research Center (H Koohestani PhD), Saveh University of Medical Sciences, Saveh, Iran; Biomedical Research Networking Center for Mental Health Network (CIBERSAM) (A Koyanagi MD), San Juan de Dios Sanitary Park, Sant Boi de Llobregat, Spain; Catalan Institution for Research and Advanced Studies (ICREA), Barcelona, Spain (A Koyanagi MD); Department of Orthopaedics (Prof N Kumar MS), Medanta Hospital, Lucknow, India; Department of Public Health (Prof T Lallukka PhD), University of Helsinki, Helsinki, Finland; Department of Otorhinolaryngology (S Lasrado MS), Father Muller Medical College, Mangalore, India; The Office of Health Policy & Legislative Affairs (W Lee PhD), University of Texas, Galveston, TX, USA; Department of Preventive Medicine, College of Medicine (Prof Y Lee PhD), Korea University, Seoul, South Korea; Department of Public Health Research (J N Malagón-Rojas MSc), National Institute of Health, Bogota, Colombia; Faculty of Medicine (J N Malagón-Rojas MSc), El Bosque University, Bogota, Colombia; Non-communicable Disease Research Center (Prof R Malekzadeh MD, S G Sepanlou MD), Health Policy Research Center (S Shahabi PhD), Community Based Psychiatric Care Research Center (M Shaygan PhD), Shiraz University of Medical Sciences, Shiraz, Iran; Research Group on Global Health and Human Development (N Malih MD), University of the Balearic Islands (UIB), Palma, Spain; Neurology Department (Prof M Mehndiratta MD), Janakpuri Super Specialty Hospital Society, New Delhi, India; Department of Neurology (Prof M Mehndiratta MD), Govind Ballabh Institute of Medical Education and Research, New Delhi, India; Forensic Medicine Division (Prof R G Menezes MD), Imam Abdulrahman Bin Faisal University, Dammam, Saudi Arabia; International Dx Department (A A Mentis MD), BGI Genomics, Copenhagen, Denmark; Faculty of Medicine (M K Mesregah MD), Menoufia University, Shebin El-Kom, Egypt; Pacific Institute for Research & Evaluation, Calverton, MD, USA (T R Miller PhD); Social Determinants of Health Center (M Mirza-Aghazadeh-Attari MD), Urmia University of Medical Sciences, Urmia, Iran (R Valizadeh PhD); Non-communicable Disease Research Center (M Mobarakabadi MD), Hamadan University of Medical Sciences, Tehran, Iran; Internal Medicine Department (Y Mohammad MD), King Saud University, Riyadh, Saudi Arabia; Health Systems and Policy Research Unit (S Mohammed PhD), Ahmadu Bello University, Zaria, Nigeria; Department of Health Care Management (S Mohammed PhD), Technical University of Berlin, Berlin, Germany; Clinical

Epidemiology and Public Health Research Unit (L Monasta DSc), Burlo Garofolo Institute for Maternal and Child Health, Trieste, Italy; School of Health & Rehabilitation Sciences (M Moni PhD), The University of Queensland, Brisbane, QLD, Australia; Department of Medicine (E Mostafavi PhD), Stanford Cardiovascular Institute (E Mostafavi PhD), Stanford University, Palo Alto, CA, USA; Health Workforce Department (T S Nair MD), World Health Organisation, Geneva, Switzerland; Department of Epidemiology (S Nejadghaderi MD, E Shaker MD), Department of International Studies (P Shobeiri MD), Non-Communicable Diseases Research Center (NCDRC), Tehran, Iran; Department of Health Sciences (S Neupane PhD), University of Tampere, Tampere, Finland; Estia Health Blakehurst (S Neupane Kandel BSN), Estia Health, Sydney, NSW, Australia; Institute for Global Health Innovations (C T Nguyen MPH), Duy Tan University, Hanoi, Viet Nam; Health Promotion Research Center (H Okati-Aliabad PhD), Zahedan University of Medical Sciences, Zahedan, Iran; Mass Communication Department (E Omer PhD), Ajman University, Dubai, United Arab Emirates; Department of Epidemiology and Population Health (A Oulhaj PhD), Khalifa University, Abu Dhabi, United Arab Emirates; Department of Medicine (Prof M O Owolabi DrM), University College Hospital, Ibadan, Ibadan, Nigeria; Privatpraxis, Heidelberg, Germany (S Panda-Jonas MD); Department of Medical Humanities and Social Medicine (Prof E Park PhD), Kosin University, Busan, South Korea; Department of Genetics (S Pawar PhD), Yale University, New Haven, CT, USA; Clinical Research Department (P Pedersini MSc), IRCCS Fondazione Don Carlo Gnocchi, Milan, Italy; Department of Orthopedics (J Pereira MS), Yenepoya Medical College, Mangalore, India; International Institute for Educational Planning (IIEP) (Prof M F P Peres MD), Albert Einstein Hospital, São Paulo, Brazil; College of Medicine (A Radfar MD), University of Central Florida, Orlando, FL, USA; Department of Population Science and Human Resource Development (M Rahman DrPH), University of Rajshahi, Rajshahi, Bangladesh; Future Technology Research Center (A Rahmani PhD), National Yunlin University of Science and Technology, Yunlin, Taiwan; University of Social Welfare and Rehabilitation Sciences, Tehran, Iran (V Rashedi PhD); Department of Biomedical Engineering (Z Ratan MSc), Khulna University of Engineering and Technology, Khulna, Bangladesh; School of Health and Society (Z Ratan MSc), University of Wollongong, Wollongong, NSW, Australia; WHO Collaborating Centre for Public Health Education and Training (D L Rawaf MRCS), Department of Primary Care and Public Health (Prof S Rawaf MD), Imperial College London, London, UK; Inovus Medical, St Helens, UK (D L Rawaf MRCS); Academic Public Health England (Prof S Rawaf MD), Public Health England, London, UK; School of

Medicine (Prof A M N Renzaho PhD), Translational Health Research Institute (Prof A M N Renzaho PhD), Western Sydney University, Campbelltown, NSW, Australia; Department of Clinical Research (L Roever PhD), Federal University of Uberlândia, Uberlândia, Brazil; Advanced Campus Governador Valadares (G d Ruela MSc), Juiz de For a Federal University, Governador Valadares, Brazil; Applied Biomedical Research Center (A Sahebkar PhD), Biotechnology Research Center (A Sahebkar PhD), Mashhad University of Medical Sciences, Mashhad, Iran; Department of Biomedical and Neuromotor Sciences (F Sanmarchi MD), University of Bologna, Bologna, Italy; Department of Neuroimmunulogy (S Shahrokhi MD), Universal Scientific Research Network (USERN), Tehran, Iran; Amity Institute of Public Health (M Shannawaz PhD), Amity University, Noida, India; Department of Physiotherapy (S Sharma PhD), Kathmandu University, Dhulikhel, Nepal; Finnish Institute of Occupational Health, Helsinki, Finland (R Shiri PhD); Medical Statistics Department (S Shivalli MD), London School of Hygiene & Tropical Medicine, London, UK; Department of Pediatrics and Child Health Nursing (M M Sibhat MSc), Dilla University, Dilla, Ethiopia; School of Medicine (Prof J A Singh MD), University of Alabama at Birmingham, Birmingham, AL, USA; Medicine Service (Prof J A Singh MD), US Department of Veterans Affairs (VA), Birmingham, AL, USA; Department of Neuroscience (M Solmi MD), University of Ottawa, Ottawa, ON, Canada; Department of Medicine (R Somayaji MD), Department of Oncology (L Yang PhD), University of Calgary, Calgary, AB, Canada; Department of Surgery (K Tan PhD), Yong Loo Lin School of Medicine (Prof N Venketasubramanian MBBS), National University of Singapore, Singapore, Singapore; Department of Community Medicine (R Thapar MD), Manipal Academy of Higher Education, Mangalore, India; Neuromuscular Rehabilitation Research Center (S Tohidast PhD), Semnan University of Medical Sciences, Semnan, Iran; Clinical Cancer Research Center (S Valadan Tahbaz PhD, S Yahyazadeh Jabbari MD), Milad General Hospital, Tehran, Iran; Department of Microbiology (S Valadan Tahbaz PhD), Islamic Azad University, Tehran, Iran; UKK Institute, Tampere, Finland (Prof T J Vasankari MD); Faculty of Medicine and Health Technology (Prof T J Vasankari MD), Tampere University, Tampere, Finland; Raffles Neuroscience Centre (Prof N Venketasubramanian MBBS), Raffles Hospital, Singapore, Singapore; Department of Health Care Administration and Economics (Prof V Vlassov MD), National Research University Higher School of Economics, Moscow, Russia; Faculty of Information Technology (B Vo PhD), HUTECH University, Ho Chi Minh City, Viet Nam; Department of Physical Therapy (T Wiangkham PhD), Naresuan University, Phitsanulok, Thailand; Caring Futures Institute (L Yadav PhD),

Flinders University, Adelaide, SA, Australia; Psychology Department (A Yadollahpour PhD), University of Sheffield, Sheffield, UK; Cancer Epidemiology and Prevention Research (L Yang PhD), Alberta Health Services, Calgary, AB, Canada; Department of Neuropsychopharmacology (N Yonemoto PhD), National Center of Neurology and Psychiatry, Kodaira, Japan; Department of Public Health (N Yonemoto PhD), Juntendo University, Tokyo, Japan; Department of Health Policy and Management (Prof M Z Younis PhD), Jackson State University, Jackson, MS, USA; School of Business & Economics (Prof M Z Younis PhD), Universiti Putra Malaysia (University of Putra Malaysia), Kuala Lumpur, Malaysia; Research and Development Department (I Zare BSc), Sina Medical Biochemistry Technologies, Shiraz, Iran; Department of Rheumatology (L M March PhD), Royal North Shore Hospital, St Leonards, NSW, Australia

Authors' contributions

Providing data or critical feedback on data sources

Mitra Abbasifard, Ahmed Abdelalim, Aidin Abedi, Hassan Abidi, Qorinah Estiningtyas Sakilah Adnani, Ali Ahmadi, Rufus Olusola Akinyemi, Abayneh Tadesse Alamer, Mansour Abdullah Alshehri, Mohammed Mansour Alshehri, Hubert Amu, Jalal Arabloo, Judie Arulappan, Tahira Ashraf, Seyyed Shamsadin Athari, Till Winfried Bärnighausen, Lope H Barrero, Amadou Barrow, Belay Boda Abule Bodicha, Srinivasa Rao Bolla, Chao Cao, Vijay Kumar Chattu, Marita Cross, Garland T Culbreth, Xiaochen Dai, Lalit Dandona, Rakhi Dandona, Katie de Luca, Samath Dhamminda Dharmaratne, Mandira Lamichhane Dhimal, Meghnath Dhimal, Hoa Thi Do, Linh Phuong Doan, Michael Ekholuenetale, Sharareh Eskandarieh, Jawad Fares, Ali Fatehizadeh, Irina Filip, Richard Charles Franklin, Balasankar Ganesan, Ahmad Ghashghaee, Mahaveer Golechha, Pouya Goleij, Bhawna Gupta, Nima Hafezi-Nejad, Arvin Haj-Mirzaian, Pawan Kumar Hamal, Jan Hartvigsen, Reza Heidari-Soureshjani, Mbuzeleni Mbuzeleni Hlongwa, Segun Emmanuel Ibitoye, Sheikh Mohammed Shariful Islam, Jost B Jonas, Gebisa Guyasa Kabito, Himal Kandel, Vikash Ranjan Keshri, Yousef Saleh Khader, Moien AB Khan, Jagdish Khubchandani, Yun Jin Kim, Adnan Kisa, Ali-Asghar Kolahi, G Anil Kumar, Savita Lasrado, Yo Han Lee, Ata Mahmoodpoor, Jeadran N Malagón-Rojas, Man Mohan Mehndiratta, Entezar Mehrabi Nasab, Ritesh G Menezes, Mohamed Kamal Mesregah, Ted R Miller, Mohammad Mirza-Aghazadeh-Attari, Shafiu Mohammed, Ali H Mokdad, Sara Momtazmanesh, Lorenzo Monasta, Mohammad Ali Moni, Ebrahim Mostafavi, Christopher J L Murray, Javad Nazari, Sandhya Neupane Kandel, Cuong Tat Nguyen, Emad Omer, Mayowa O Owolabi, Songhomitra Panda-Jonas, Anamika Pandey, Shrikant Pawar, Paolo Pedersini, Jeevan Pereira, Mario F P Peres, Amir Radfar, Shahram Rahimi-Dehgolan, Vafa Rahimi-Movaghar, Amir Masoud Rahmani, Chythra R Rao, Salman Rawaf, Andre M N Renzaho, Leonardo Roever, Basema Saddik, Mohammed Shannawaz, Rahman Shiri, Parnian Shobeiri, Ambrish Singh, Jasvinder A Singh, Jaimie D Steinmetz, Ker-Kan Tan, Sahel Valadan Tahbaz, Tommi Juhani Vasankari, Narayanaswamy Venketasubramanian, Vasily Vlassov, Bay Vo, Taweewat Wiangkham, Ai-Min Wu, Ali Yadollahpour, Seyed Hossein Yahyazadeh Jabbari, Naohiro Yonemoto, Mustafa Z Younis, Iman Zare, and Mohammad Zoladl.

Developing methods or computational machinery

Qorinah Estiningtyas Sakilah Adnani, Ali Ahmadi, Abayneh Tadesse Alamer, Hubert Amu, Garland T Culbreth, Xiaochen Dai, Ali Fatehizadeh, Kai Glenn Fukutaki, Mahaveer Golechha, Adnan Kisa, Ali H Mokdad, Christopher J L Murray, Emad Omer, Amir Masoud Rahmani, Zahed Rezaei, Jaimie D Steinmetz, Bay Vo, and Stein Emil Vollset.

Providing critical feedback on methods or results

Semagn Mekonnen Abate, Mitra Abbasifard, Ahmed Abdelalim, Aidin Abedi, Hassan Abidi, Qorinah Estiningtyas Sakilah Adnani, Ali Ahmadi, Rufus Olusola Akinyemi, Abayneh Tadesse Alamer, Adugnaw Zeleke Alem, Yousef Alimohamadi, Mansour Abdullah Alshehri, Mohammed Mansour Alshehri, Hosam Alzahrani, Saeed Amini, Sohrab Amiri, Hubert Amu, Catalina Liliana Andrei, Tudorel Andrei, Benny Antony, Jalal Arabloo, Judie Arulappan, Ashokan Arumugam, Tahira Ashraf, Seyyed Shamsadin Athari, Nefsu Awoke, Sina Azadnajafabad, Till Winfried Bärnighausen, Lope H Barrero, Amadou Barrow, Lindsay M Bearne, Alemshet Yirga Berhie, Vijayalakshmi S Bhojaraja, Ali Bijani, Belay Boda Abule Bodicha, Javier Brazo-Sayavera, Andrew M Briggs, Rachelle Buchbinder, Chao Cao, Periklis Charalampous, Vijay Kumar Chattu, Flavia M Cicuttini, Marita Cross, Garland T Culbreth, Sarah Cuschieri, Omid Dadras, Xiaochen Dai, Lalit Dandona, Rakhi Dandona, Katie de Luca, Azizallah Dehghan, Mandira Lamichhane Dhimal, Meghnath Dhimal, Daniel Diaz, Mojtaba Didehdar, Lankamo Ena Digesa, Mengistie Diress, Hoa Thi Do, Linh Phuong Doan, Michael Ekholuenetale, Muhammed Elhadi, Sharareh Eskandarieh, Shahriar Faghani, Jawad Fares, Ali Fatehizadeh, Paulo H Ferreira, Getahun Fetensa, Irina Filip, Florian Fischer, Richard Charles Franklin, Balasankar Ganesan, Belete Negese Belete Gemeda, Motuma Erena Getachew, Ahmad Ghashghaee, Tiffany K Gill, Mahaveer Golechha, Bhawna Gupta, Nima Hafezi-Nejad, Arvin Haj-Mirzaian, Pawan Kumar Hamal, Asif Hanif, Netanja I Harlianto, Jan Hartvigsen, Jeffrey J Hebert, Golnaz Heidari, Mohammad Heidari, Reza Heidari-Soureshjani, Mbuzeleni Mbuzeleni Hlongwa, Mohammad-Salar Hosseini, Alexander Kevin Hsiao, Segun Emmanuel Ibitoye, Irena M Ilic, Milena D Ilic, Sheikh Mohammed Shariful Islam, Manthan Dilipkumar Janodia, Ravi Prakash Jha, Har Ashish Jindal, Jost B Jonas, Gebisa Guyasa Kabito, Himal Kandel, Rimple Jeet Kaur, Vikash Ranjan Keshri, Yousef Saleh Khader, Ejaz Ahmad Khan, Md Jobair Khan, Moien AB Khan, Hamid Reza Khayat Kashani, Jagdish Khubchandani, Yun Jin Kim, Adnan Kisa, Jitka Klugarová, Hamid

Reza Koohestani, Jacek A Kopec, Ai Koyanagi, G Anil Kumar, Narinder Kumar, Tea Lallukka, Savita Lasrado, Wei-Chen Lee, Yo Han Lee, Ata Mahmoodpoor, Mohammad-Reza Malekpour, Reza Malekzadeh, Narges Malih, Entezar Mehrabi Nasab, Ritesh G Menezes, Alexios-Fotios A Mentis, Mohamed Kamal Mesregah, Ted R Miller, Mohammad Mirza-Aghazadeh-Attari, Maryam Mobarakabadi, Yousef Mohammad, Esmaeil Mohammadi, Shafiu Mohammed, Ali H Mokdad, Sara Momtazmanesh, Mohammad Ali Moni, Ebrahim Mostafavi, Christopher J L Murray, Tapas Sadasivan Nair, Javad Nazari, Seyed Aria Nejadghaderi, Subas Neupane, Sandhya Neupane Kandel, Cuong Tat Nguyen, Ali Nowroozi, Hassan Okati-Aliabad, Emad Omer, Abderrahim Oulhaj, Mayowa O Owolabi, Songhomitra Panda-Jonas, Anamika Pandey, Eun-Kee Park, Shrikant Pawar, Paolo Pedersini, Jeevan Pereira, Mario F P Peres, Ionela-Roxana Petcu, Amir Radfar, Shahram Rahimi-Dehgolan, Vafa Rahimi-Movaghar, Mosiur Rahman, Amir Masoud Rahmani, Nazanin Rajai, Chythra R Rao, Vahid Rashedi, Mohammad-Mahdi Rashidi, Zubair Ahmed Ratan, David Laith Rawaf, Salman Rawaf, Andre M N Renzaho, Negar Rezaei, Zahed Rezaei, Leonardo Roever, Guilherme de Andrade, Guilherme de Ruela, Basema Saddik, Saeid Safiri, Sana Salehi, Francesco Sanmarchi, Sadaf G Sepanlou, Saeed Shahabi, Shayan Shahrokhi, Elaheh Shaker, Mohammed Shannawaz, Saurab Sharma, Rahim Ali Sheikhi, Jeevan K Shetty, Rahman Shiri, Siddharudha Shivalli, Parnian Shobeiri, Migbar Mekonnen Sibhat, Ambrish Singh, Jasvinder A Singh, Amanda E Smith, Marco Solmi, Ranjani Somayaji, Jaimie D Steinmetz, Ker-Kan Tan, Rekha Thapar, Seyed Abolfazl Tohidast, Sahel Valadan Tahbaz, Rohollah Valizadeh, Narayanaswamy Venketasubramanian, Vasily Vlassov, Bay Vo, Taweewat Wiangkham, Lalit Yadav, Ali Yadollahpour, Seyed Hossein Yahyazadeh Jabbari, Lin Yang, Fereshteh Yazdanpanah, Naohiro Yonemoto, Mustafa Z Younis, Armin Zarrintan, and Mohammad Zoladl.

Drafting the work or revising is critically for important intellectual content

Mohsen Abbasi-Kangevari, Zeinab Abbasi-Kangevari, Ahmed Abdelalim, Aidin Abedi, Hassan Abidi, Qorinah Estiningtyas Sakilah Adnani, Ali Ahmadi, Rufus Olusola Akinyemi, Abayneh Tadesse Alamer, Adugnaw Zeleke Alem, Mansour Abdullah Alshehri, Hosam Alzahrani, Saeed Amini, Sohrab Amiri, Hubert Amu, Catalina Liliana Andrei, Tudorel Andrei, Benny Antony, Jalal Arabloo, Judie Arulappan, Ashokan Arumugam, Seyyed Shamsadin Athari, Sina Azadnajafabad, Till Winfried Bärnighausen, Lope H Barrero, Amadou Barrow, Akbar Barzegar, Lindsay M Bearne, Isabela M Bensenor, Bharti Bhandari

Bhandari, Vijayalakshmi S Bhojaraja, Belay Boda Abule Bodicha, Javier Brazo-Sayavera, Rachelle Buchbinder, Chao Cao, Vijay Kumar Chattu, Flavia M Cicuttini, Benjamin Clarsen, Marita Cross, Garland T Culbreth, Katie de Luca, Takele Gezahegn G Demie, Edgar Denova-Gutiérrez, Syed Masudur Rahman Dewan, Samath Dhamminda Dharmaratne, Mandira Lamichhane Dhimal, Meghnath Dhimal, Daniel Diaz, Mojtaba Didehdar, Linh Phuong Doan, Muhammed Elhadi, Sharareh Eskandarieh, Shahriar Faghani, Jawad Fares, Ali Fatehizadeh, Getahun Fetensa, Paulo H Ferreira, Irina Filip, Florian Fischer, Balasankar Ganesan, Motuma Erena Getachew, Tiffany K Gill, Bhawna Gupta, Nima Hafezi-Nejad, Arvin Haj-Mirzaian, Pawan Kumar Hamal, Netanja I Harlianto, Jan Hartvigsen, Hamidreza Hasani, Simon I Hay, Jeffrey J Hebert, Golnaz Heidari, Mohammad-Salar Hosseini, Alexander Kevin Hsiao, Ivo Iavicoli, Segun Emmanuel Ibitoye, Irena M Ilic, Milena D Ilic, Sheikh Mohammed Shariful Islam, Ravi Prakash Jha, Har Ashish Jindal, Jost B Jonas, Gebisa Guyasa Kabito, Himal Kandel, Rimple Jeet Kaur, Vikash Ranjan Keshri, Ejaz Ahmad Khan, Md Jobair Khan, Moien AB Khan, Jagdish Khubchandani, Yun Jin Kim, Adnan Kisa, Jacek A Kopec, Ai Koyanagi, Narinder Kumar, Tea Lallukka, Savita Lasrado, Ata Mahmoodpoor, Mohammad-Reza Malekpour, Reza Malekzadeh, Narges Malih, Entezar Mehrabi Nasab, Ritesh G Menezes, Alexios-Fotios A Mentis, Mohamed Kamal Mesregah, Ted R Miller, Yousef Mohammad, Esmaeil Mohammadi, Shafiu Mohammed, Ali H Mokdad, Sara Momtazmanesh, Lorenzo Monasta, Mohammad Ali Moni, Ebrahim Mostafavi, Christopher J L Murray, Seyed Aria Nejadghaderi, Subas Neupane, Sandhya Neupane Kandel, Cuong Tat Nguyen, Ali Nowroozi, Mayowa O Owolabi, Songhomitra Panda-Jonas, Shrikant Pawar, Paolo Pedersini, Mario F P Peres, Ionela-Roxana Petcu, Mohammadreza Pourahmadi, Amir Radfar, Vafa Rahimi-Movaghar, Nazanin Rajai, Chythra R Rao, Vahid Rashedi, Zubair Ahmed Ratan, David Laith Rawaf, Salman Rawaf, Andre M N Renzaho, Negar Rezaei, Zahed Rezaei, Leonardo Roever, Guilherme de Andrade, Guilherme de Ruela, Basema Saddik, Saeid Safiri, Amirhossein Sahebkar, Francesco Sanmarchi, Sadaf G Sepanlou, Saeed Shahabi, Elaheh Shaker, MohammadBagher Shamsi, Mohammed Shannawaz, Saurab Sharma, Maryam Shaygan, Jeevan K Shetty, Siddharudha Shivalli, Parnian Shobeiri, Ambrish Singh, Jasvinder A Singh, Helen Slater, Marco Solmi, Ranjani Somayaji, Jaimie D Steinmetz, Ker-Kan Tan, Seyed Abolfazl Tohidast, Sahel Valadan Tahbaz, Tommi Juhani Vasankari, Narayanaswamy Venketasubramanian, Vasily Vlassov, Stein Emil Vollset, Yuan-Pang Wang, Taweewat Wiangkham, Ai-Min Wu, Lalit Yadav, Seyed Hossein Yahyazadeh Jabbari, Lin Yang, Naohiro Yonemoto, Iman Zare, Armin Zarrintan, and Mohammad Zoladl.

Managing the estimation or publications process

Ali Ahmadi, Abayneh Tadesse Alamer, Mohammed Mansour Alshehri, Saeed Amini, Judie Arulappan, Katie de Luca, Mojtaba Didehdar, Ali Fatehizadeh, Pawan Kumar Hamal, Simon I Hay, Ali H Mokdad, Christopher J L Murray, Javad Nazari, Amanda E Smith, Jaimie D Steinmetz, Stein Emil Vollset, Ai-Min Wu.

Supplemental Figure 1: Distribution of data sources of low back pain

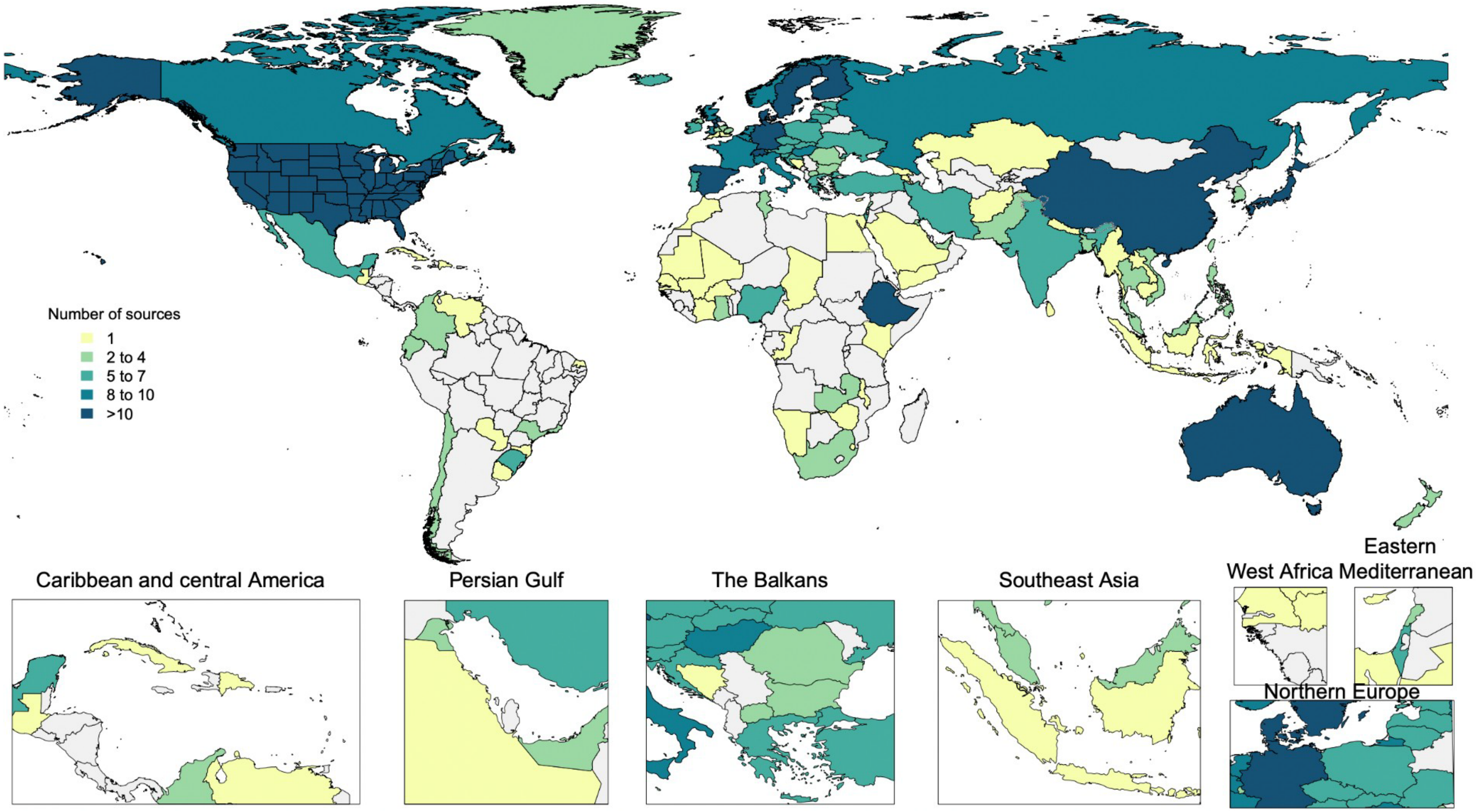

Note: numbers for US states are based on national claims data rather than >10 sources for all states

Supplemental Figure 2: Global projected number of cases of low back pain from 1990 to 2050 by sex

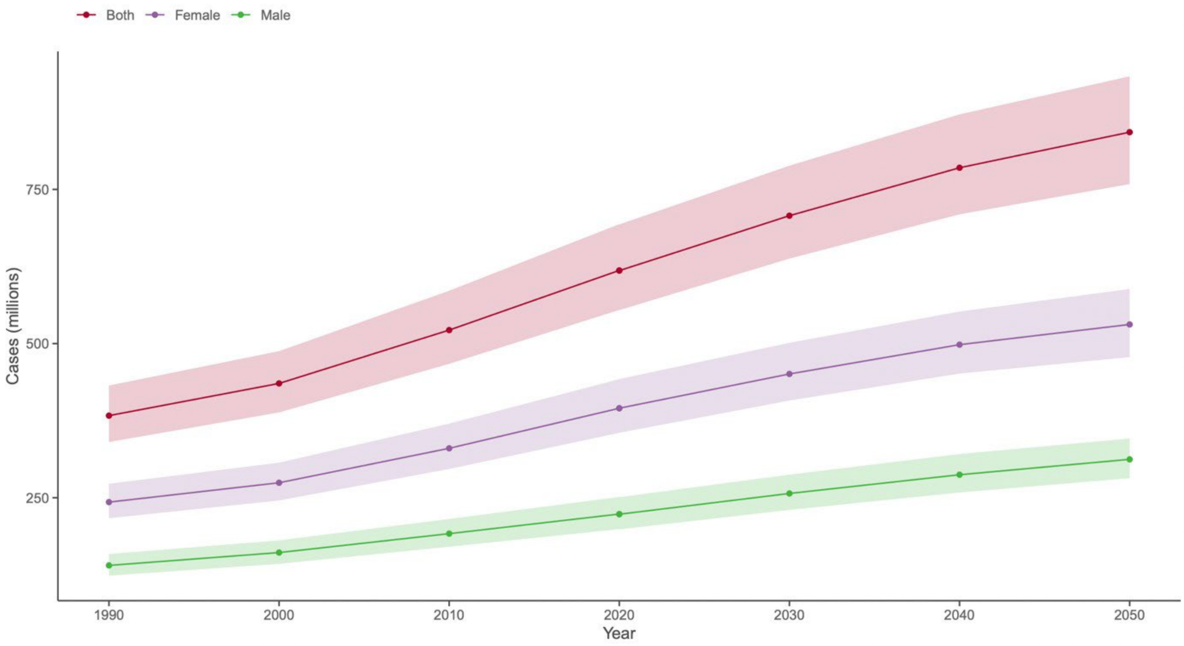

Supplemental Figure 3: Projected number of cases of low back pain from 1990 to 2050 for GBD regions

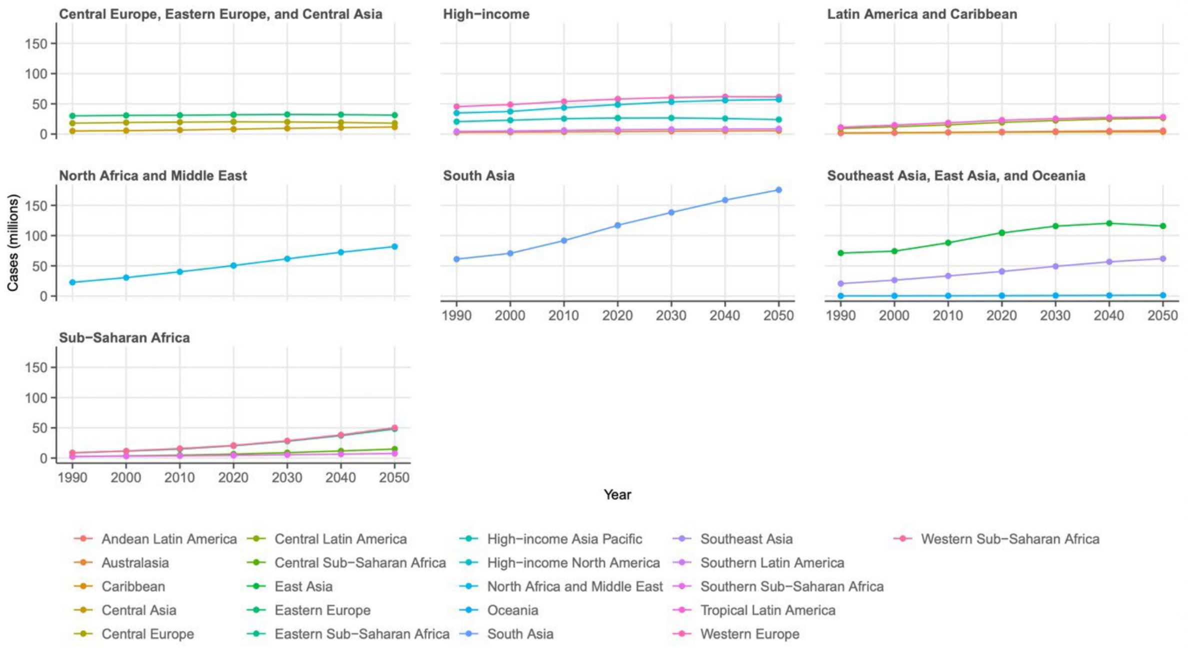

Supplemental Figure 4: Global projections to 2050 of low back pain age-standardised prevalence

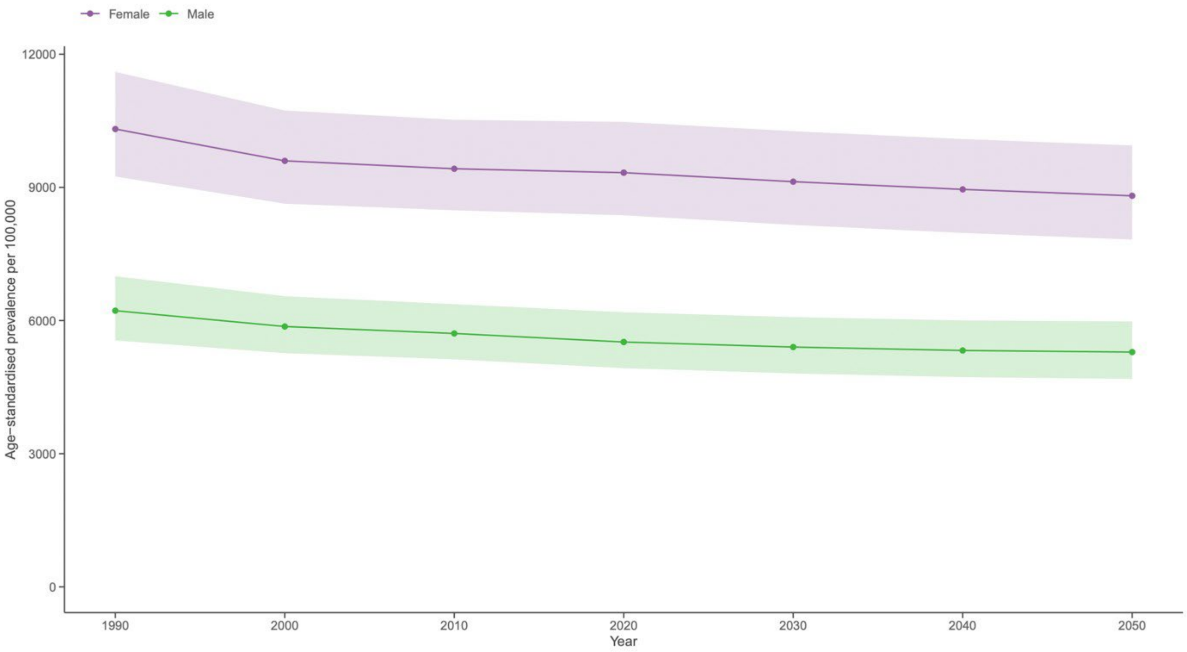

Supplemental Table 1: MR-BRT crosswalk adjustment factors for low back pain

Data input	Reference or alternative case definition	Gamma	Beta coefficient, logit (95% CI)
Point prevalence	Ref	0.59	-
Recall periods of 1 week to 1 month			0.03<br>(-0.03 to 0.09)
Recall periods between 2 months and 1 year	Alt		0.73<br>(0.68 to 0.78)
Activity-limiting low back pain	Alt		–1.65<br>(–1.66 to –1.63)
USA claims data – 2000	Alt		–1.28<br>(–1.59 to –0.97)
USA claims data – 2010–2012, 2014–2017	Alt		–0.66<br>(–0.81 to –0.51)

If the coefficient is negative, then the alternative is adjusted up to the reference. If the log/logit beta coefficient is positive, then the alternative is adjusted down to the reference.

**MR-BRT crosswalk adjustments can be interpreted as the factor the alternative case definition is adjusted to reflect what it would have been had it been measured using the reference case definition.*

Supplemental Table 2: Covariates used in low back pain DisMod-MR 2.1 model

Covariate	Type	Parameter	Exponentiated beta (95% uncertainty interval)
Log-transformed age-standardised SEV scalar: back pain	Country-level	Prevalence	2.12 (2.12–2.14)

Supplemental Table 3: Severity levels for low back pain in GBD 2021 and associated disability weights (DW)

Severity level	Lay description	DW (95% confidence intervals)
Low back pain, mild	This person has mild back pain, which causes some difficulty dressing, standing, and lifting things.	0.020 (0.011–0.035)
Low back pain, moderate	This person has moderate back pain, which causes difficulty dressing, sitting, standing, walking, and lifting things.	0.054 (0.035–0.079)
Low back pain, severe without leg pain	This person has severe back pain, which causes difficulty dressing, sitting, standing, walking, and lifting things. The person sleeps poorly and feels worried.	0.272 (0.182–0.373)
Low back pain, severe with leg pain	This person has severe back and leg pain, which causes difficulty dressing, sitting, standing, walking, and lifting things. The person sleeps poorly and feels worried.	0.325 0.219–0.446)
Low back pain, most severe without leg pain	This person has constant back pain, which causes difficulty dressing, sitting, standing, walking, and lifting things. The person sleeps poorly, is worried, and has lost some enjoyment in life.	0.372 (0.250–0.506)
Low back pain, most severe with leg pain	This person has constant back and leg pain, which causes difficulty dressing, sitting, standing, walking, and lifting things. The person sleeps poorly, is worried, and has lost some enjoyment in life.	0.384 (0.256–0.518)

Supplemental Table 4. Proportion of low back pain with leg pain

Age (years)	Proportion with leg pain
5–9	9.4% (9.1–9.8)
10–14	10.9% (10.7–11.1)
15–19	15.9% (15.8–16.1)
20–24	23.2% (23.0–23.4)
25–29	28.8% (28.6–28.9)
30–34	31.4% (31.3–31.6)
35–39	33.1% (32.9–33.2)
40–44	34.3% (34.2–34.4)
45–49	35.5% (35.4–35.6)
50–54	36.4% (36.3–36.5)
55–59	37.1% (37.0–37.2)
60–64	37.4% (37.3–37.5)
65–69	37.1% (36.9–37.3)
70–74	36.5% (36.4–36.7)
75–79	35.0% (34.8–35.2)
80–84	32.1% (31.9–32.4)
85–89	28.3% (28.0–28.5)
90–94	23.7% (23.2–24.2)
95–100	19.2% (18.2–20.2)

Supplemental Table 5: Prevalence, years lived with disability (YLDs), age-standardised rates of prevalence, and YLDs per 100,000 population in 2020, and percentage change between 1990 and 2020 for low back pain globally, by country

Location	Number of prevalent cases (95% UI)	Age-standardised prevalence rate per 100 000 (95% UI)	% change in age-standardised prevalence rate from 1990 to 2020 (95% UI)	Number of YLDs (95% UI)	Age-standardised rate of YLDs per 100 000 (95% UI)	% change in age-standardised rate of YLDs per 100 000 from 1990 to 2020 (95% UI)
Armenia	365 000 (321 000–409 000)	9 860 (8 720–11 000)	0·2 (-2·8–3·0)	41 000 (28 700–53 700)	1110·0 (770·0–1450·0)	0·4 (-3·2–3·5)
Azerbaijan	997 000 (891 000–1 140 000)	9 150 (8 300–10 300)	1·1 (-2·8–6·0)	113 000 (79 000–153 000)	1030·0 (718·0–1350·0)	1·0 (-3·2–5·3)
Georgia	406 000 (364 000–454 000)	8 430 (7 430–9 490)	-1·6 (-5·6–4·3)	45 100 (31 800–59 800)	944·0 (655·0–1230·0)	-2·1 (-6·2–3·8)
Kazakhstan	1 760 000 (1 530 000–1 980 000)	9 400 (8 270–10 500)	-0·3 (-4·6–3·7)	198 000 (138 000–260 000)	1050·0 (728·0–1360·0)	-0·2 (-4·9–4·1)
Kyrgyzstan	523 000 (459 000–589 000)	9 120 (8 110–10 200)	-3·4 (-7·4–-0·0)	59 100 (40 900–78 000)	1020·0 (711·0–1340·0)	-3·1 (-8·0–0·3)
Mongolia	278 000 (241 000–318 000)	8 960 (7 830–10 000)	-2·7 (-6·1–0·5)	31 500 (22 200–41 600)	1000·0 (698·0–1310·0)	-2·6 (-6·2–0·9)
Tajikistan	642 000 (571 000–734 000)	8 500 (7 560–9 520)	-3·7 (-6·6–-0·2)	73 000 (51 200–95 300)	955·0 (671·0–1220·0)	-3·7 (-6·6–-0·4)
Turkmenistan	414 000 (366 000–468 000)	8 810 (7 850–9 840)	-3·4 (-7·0–-0·4)	46 900 (32 200–62 900)	991·0 (679·0–1300·0)	-3·1 (-7·0–0·4)
Uzbekistan	2 670 000 (2 320 000–3 020 000)	9 120 (8 060–10 200)	-1·1 (-4·8–2·2)	302 000 (210 000–401 000)	1020·0 (710·0–1330·0)	-1·2 (-5·3–2·5)

Albania	449 000 (401 000–502 000)	13 000 (11 600–14 600)	1·3 (-2·0–4·8)	50 300 (35 600–66 300)	1460·0 (1030·0–1900·0)	1·5 (-1·8–5·3)
Bosnia and Herzegovina	562 000 (496 000–629 000)	12 400 (11 200–13 900)	3·6 (-0·1–7·8)	62 500 (44 400–83 600)	1390·0 (980·0–1830·0)	3·5 (-0·2–8·0)
Bulgaria	1 230 000 (1 080 000–1 370 000)	12 300 (11 000–13 800)	-1·7 (-5·1–3·0)	137 000 (95 800–178 000)	1380·0 (960·0–1810·0)	-1·6 (-5·2–2·9)
Croatia	730 000 (644 000–811 000)	12 000 (10 600–13 400)	-1·7 (-5·5–2·4)	81 000 (56 500–107 000)	1350·0 (946·0–1760·0)	-1·7 (-6·2–2·6)
Czechia	1 960 000 (1 730 000–2 170 000)	13 100 (11 600–14 700)	-3·5 (-8·1–0·0)	217 000 (152 000–286 000)	1460·0 (1020·0–1910·0)	-3·2 (-8·2–0·3)
Hungary	1 930 000 (1 720 000–2 120 000)	14 000 (12 600–15 500)	-1·6 (-4·7–1·6)	214 000 (149 000–286 000)	1570·0 (1090·0–2070·0)	-1·0 (-4·4–2·7)
Montenegro	101 000 (91 300–114 000)	12 700 (11 400–14 300)	1·4 (-2·3–5·4)	11 300 (7 990–14 900)	1420·0 (995·0–1870·0)	1·1 (-2·6–5·5)
North Macedonia	326 000 (285 000–364 000)	11 700 (10 400–13 200)	0·2 (-2·7–3·3)	36 400 (25 700–47 700)	1310·0 (924·0–1710·0)	-0·0 (-3·7–3·3)
Poland	6 790 000 (6 120 000–7 540 000)	12 800 (11 400–14 300)	-3·0 (-4·0–-2·1)	755 000 (529 000–985 000)	1430·0 (994·0–1860·0)	-2·3 (-3·4–-1·3)
Romania	3 420 000 (3 040 000–3 810 000)	12 800 (11 300–14 400)	-5·3 (-8·7–-2·1)	381 000 (270 000–501 000)	1430·0 (1010·0–1890·0)	-4·8 (-8·8–-1·4)
Serbia	1 510 000 (1 340 000–1 670 000)	12 800 (11 500–14 400)	0·5 (-3·7–3·8)	168 000 (118 000–223 000)	1440·0 (1010·0–1900·0)	0·5 (-3·5–3·7)
Slovakia	933 000 (833 000–1 040 000)	12 700 (11 500–14 300)	-4·1 (-7·5–-0·4)	104 000 (74 000–137 000)	1420·0 (1000·0–1900·0)	-3·8 (-8·1–0·6)
Slovenia	350 000 (311 000–388 000)	11 600 (10 300–13 100)	-1·7 (-5·6–2·2)	38 700 (27 700–51 600)	1300·0 (913·0–1720·0)	-1·2 (-5·5–3·5)
Belarus	1 410 000 (1 240 000–1 580 000)	10 900 (9 670–12 200)	-0·5 (-3·9–2·8)	157 000 (111 000–209 000)	1210·0 (852·0–1590·0)	-0·6 (-4·3–3·2)

Estonia	203 000 (178 000–229 000)	10 600 (9 370–11 800)	-1·6 (-5·2–2·2)	22 400 (15 700–29 300)	1180·0 (837·0–1530·0)	-1·1 (-5·1–3·5)
Latvia	299 000 (264 000–332 000)	10 500 (9 310–11 900)	-1·7 (-5·3–1·7)	32 900 (23 100–43 200)	1180·0 (822·0–1530·0)	-1·3 (-5·5–2·3)
Lithuania	442 000 (389 000–491 000)	10 700 (9 590–12 100)	-2·1 (-6·3–1·9)	48 500 (33 800–63 800)	1190·0 (830·0–1550·0)	-2·2 (-6·1–2·6)
Republic of Moldova	527 000 (468 000–589 000)	10 600 (9 530–11 900)	-2·1 (-5·0–1·3)	58 600 (41 100–76 200)	1190·0 (817·0–1540·0)	-1·8 (-5·7–1·7)
Russian Federation	21 500 000 (19 200 000–24 000 000)	10 900 (9 840–12 100)	-3·7 (-4·3–-3·0)	2 370 000 (1 660 000–3 090 000)	1210·0 (840·0–1550·0)	-3·6 (-4·4–-2·7)
Ukraine	7 550 000 (6 700 000–8 380 000)	12 300 (10 900–13 800)	-4·0 (-6·4–-1·1)	834 000 (589 000–1 100 000)	1370·0 (960·0–1800·0)	-3·9 (-6·5–-0·5)
Australia	3 410 000 (3 020 000–3 850 000)	10 900 (9 640–12 500)	-6·3 (-10·3–-1·6)	380 000 (264 000–486 000)	1230·0 (851·0–1560·0)	-6·2 (-10·8–-1·2)
New Zealand	717 000 (630 000–799 000)	11 600 (10 300–13 100)	-3·5 (-7·2–0·3)	79 900 (55 800–104 000)	1300·0 (906·0–1660·0)	-2·9 (-6·4–0·9)
Brunei Darussalam	37 800 (33 400–43 000)	8 400 (7 520–9 450)	-2·1 (-5·3–1·5)	4 320 (2 990–5 650)	948·0 (659·0–1220·0)	-2·2 (-5·7–1·5)
Japan	19 500 000 (17 400 000–21 600 000)	10 200 (9 050–11 600)	-6·6 (-7·7–-5·7)	2 180 000 (1 510 000–2 880 000)	1170·0 (805·0–1510·0)	-6·4 (-7·4–-5·3)
Republic of Korea	6 400 000 (5 700 000–7 170 000)	8 750 (7 740–9 730)	-4·6 (-9·0–-0·9)	723 000 (500 000–951 000)	990·0 (689·0–1280·0)	-4·4 (-8·8–0·1)
Singapore	533 000 (477 000–608 000)	7 210 (6 470–8 180)	-5·2 (-9·4–-2·2)	60 900 (42 600–80 700)	822·0 (575·0–1070·0)	-5·1 (-9·1–-1·4)
Canada	4 200 000 (3 730 000–4 690 000)	8 680 (7 710–9 810)	-5·5 (-10·0–-1·3)	468 000 (322 000–609 000)	975·0 (680·0–1240·0)	-6·0 (-10·4–-1·5)
Greenland	5 570 (4 920–6 370)	8 510 (7 600–9 730)	-3·1 (-7·0–1·2)	624 (441–818)	950·0 (665·0–1220·0)	-3·0 (-7·3–1·2)

United States of America	44 600 000 (41 600 000–47 400 000)	10 700 (10 100–11 400)	-5·8 (-11·9–-0·1)	4 880 000 (3 510 000–6 210 000)	1180·0 (837·0–1500·0)	-7·0 (-13·1–-1·8)
Argentina	4 640 000 (4 150 000–5 220 000)	9 230 (8 240–10 400)	1·1 (-2·9–6·0)	520 000 (363 000–661 000)	1030·0 (725·0–1320·0)	0·6 (-3·3–5·9)
Chile	2 040 000 (1 810 000–2 280 000)	9 360 (8 270–10 500)	1·3 (-2·1–5·5)	228 000 (160 000–294 000)	1050·0 (737·0–1340·0)	1·2 (-2·9–6·2)
Uruguay	395 000 (351 000–441 000)	9 440 (8 390–10 600)	9·6 (5·1–14·8)	44 000 (30 200–57 500)	1060·0 (728·0–1370·0)	9·2 (5·1–14·4)
Andorra	10 600 (9 330–11 900)	9 100 (8 000–10 300)	-2·0 (-5·1–1·5)	1 180 (829–1 560)	1020·0 (713·0–1310·0)	-2·4 (-5·9–1·3)
Austria	1 010 000 (902 000–1 120 000)	8 010 (7 150–8 990)	-6·0 (-9·8–-3·1)	112 000 (78 100–148 000)	898·0 (630·0–1150·0)	-5·9 (-10·3–-2·2)
Belgium	1 440 000 (1 270 000–1 620 000)	9 290 (8 310–10 500)	-1·9 (-5·8–1·5)	159 000 (111 000–208 000)	1040·0 (717·0–1340·0)	-2·4 (-6·7–1·6)
Cyprus	159 000 (142 000–177 000)	9 410 (8 360–10 600)	-0·2 (-3·9–4·6)	17 800 (12 400–23 100)	1060·0 (738·0–1370·0)	-0·1 (-3·8–5·0)
Denmark	853 000 (735 000–974 000)	10 800 (9 300–12 800)	-15·2 (-23·7–-6·6)	95 100 (65 200–126 000)	1210·0 (830·0–1590·0)	-15·0 (-23·7–-6·3)
Finland	656 000 (582 000–728 000)	8 270 (7 470–9 210)	-4·2 (-8·0–0·6)	72 500 (50 700–95 100)	927·0 (644·0–1180·0)	-4·2 (-8·0–0·5)
France	8 500 000 (7 610 000–9 420 000)	9 470 (8 420–10 700)	0·3 (-4·6–4·1)	939 000 (662 000–1 240 000)	1060·0 (731·0–1380·0)	0·2 (-4·2–4·3)
Germany	13 400 000 (11 800 000–14 900 000)	11 000 (9 710–12 300)	-4·3 (-8·3–-0·2)	1 490 000 (1 030 000–1 940 000)	1230·0 (846·0–1570·0)	-4·5 (-8·0–-0·2)
Greece	1 330 000 (1 180 000–1 480 000)	8 920 (7 970–10 000)	-0·1 (-3·1–3·5)	148 000 (104 000–193 000)	1000·0 (706·0–1300·0)	0·0 (-3·4–3·8)
Iceland	41 200 (36 900–45 800)	9 520 (8 460–10 700)	-5·3 (-9·2–-2·4)	4 620 (3 180–6 020)	1070·0 (739·0–1380·0)	-5·2 (-8·9–-1·3)

Ireland	587 000 (524 000–649 000)	9 680 (8 700–10 700)	-0·1 (-4·4–4·6)	65 700 (45 500–87 000)	1090·0 (749·0–1420·0)	-0·2 (-4·8–4·1)
Israel	985 000 (881 000–1 100 000)	9 730 (8 670–11 000)	-3·4 (-7·1–-0·2)	110 000 (75 600–144 000)	1100·0 (749·0–1440·0)	-3·5 (-7·1–0·9)
Italy	8 490 000 (7 530 000–9 430 000)	9 500 (8 430–10 600)	0·4 (-0·6–1·9)	940 000 (652 000–1 250 000)	1070·0 (742·0–1370·0)	0·9 (-0·4–2·3)
Luxembourg	76 600 (68 600–86 200)	9 400 (8 460–10 700)	-2·8 (-6·7–0·1)	8 570 (5 870–11 300)	1060·0 (724·0–1380·0)	-2·6 (-7·3–1·0)
Malta	62 600 (55 000–68 700)	9 960 (8 910–11 100)	0·2 (-3·0–4·6)	6 990 (4 880–9 190)	1120·0 (770·0–1450·0)	0·1 (-3·0–5·0)
Monaco	5 240 (4 530–5 870)	9 110 (8 180–10 300)	-2·8 (-6·8–0·5)	582 (404–775)	1020·0 (716·0–1340·0)	-3·1 (-7·8–0·3)
Netherlands	1 950 000 (1 750 000–2 190 000)	8 210 (7 260–9 090)	-2·4 (-6·1–1·2)	218 000 (152 000–288 000)	923·0 (645·0–1190·0)	-2·8 (-6·9–0·9)
Norway	625 000 (560 000–691 000)	8 800 (7 820–9 840)	-4·8 (-6·0–-3·3)	69 700 (48 200–91 200)	989·0 (687·0–1270·0)	-4·3 (-5·7–-2·8)
Portugal	1 570 000 (1 410 000–1 730 000)	10 100 (8 930–11 200)	-1·2 (-5·0–1·8)	174 000 (122 000–230 000)	1130·0 (780·0–1450·0)	-0·9 (-4·5–2·2)
San Marino	4 110 (3 640–4 590)	9 150 (8 080–10 300)	-2·6 (-5·4–1·2)	457 (317–605)	1030·0 (706·0–1330·0)	-3·1 (-6·0–1·0)
Spain	5 320 000 (4 720 000–6 010 000)	8 150 (7 300–9 310)	-4·7 (-10·5–2·3)	590 000 (408 000–788 000)	913·0 (634·0–1180·0)	-5·0 (-11·2–2·5)
Sweden	1 010 000 (887 000–1 170 000)	7 070 (6 310–8 090)	19·4 (12·1–27·2)	112 000 (78 300–148 000)	794·0 (551·0–1030·0)	20·0 (12·5–28·4)
Switzerland	1 220 000 (1 090 000–1 360 000)	10 100 (8 960–11 300)	-10·4 (-16·0–-3·9)	136 000 (94 200–178 000)	1130·0 (783·0–1470·0)	-10·5 (-16·3–-4·4)
United Kingdom	8 460 000 (7 560 000–9 370 000)	9 440 (8 380–10 600)	1·1 (-0·6–2·7)	939 000 (648 000–1 230 000)	1060·0 (731·0–1360·0)	0·6 (-0·9–2·3)

Bolivia (Plurinational State of)	661 000 (584 000–737 000)	6 120 (5 440–6 770)	0·9 (-2·1–4·7)	74 000 (51 400–93 500)	682·0 (473·0–865·0)	0·5 (-2·6–4·5)
Ecuador	939 000 (844 000–1 060 000)	5 480 (4 950–6 150)	-5·0 (-9·9–-0·1)	105 000 (73 100–135 000)	612·0 (425·0–786·0)	-5·7 (-10·7–-1·1)
Peru	2 010 000 (1 780 000–2 280 000)	5 770 (5 150–6 540)	3·0 (-0·0–6·5)	226 000 (156 000–285 000)	648·0 (448·0–819·0)	2·8 (-1·0–6·8)
Antigua and Barbuda	6 090 (5 390–6 840)	5 900 (5 250–6 590)	-0·2 (-3·9–3·5)	682 (473–895)	659·0 (454·0–857·0)	-0·9 (-4·5–3·2)
Bahamas	24 900 (21 800–28 200)	5 910 (5 230–6 640)	-0·5 (-3·8–3·9)	2 800 (1 950–3 570)	662·0 (459·0–837·0)	-1·1 (-4·9–3·1)
Barbados	23 500 (20 900–26 600)	5 960 (5 270–6 810)	-1·0 (-5·1–3·5)	2 630 (1 820–3 420)	669·0 (463·0–853·0)	-1·4 (-5·7–4·0)
Belize	22 900 (20 100–26 100)	6 060 (5 400–6 830)	0·7 (-2·1–4·5)	2 570 (1 800–3 350)	678·0 (475·0–877·0)	0·0 (-3·0–4·6)
Bermuda	5 630 (5 010–6 300)	6 060 (5 370–6 840)	-1·5 (-4·9–2·8)	629 (435–827)	683·0 (467·0–879·0)	-1·7 (-5·7–3·5)
Cuba	871 000 (784 000–941 000)	5 710 (5 220–6 170)	-5·3 (-10·7–-1·1)	97 200 (68 200–126 000)	641·0 (451·0–818·0)	-5·6 (-11·1–-0·8)
Dominica	4 790 (4 260–5 380)	5 980 (5 300–6 690)	-3·3 (-7·3–1·1)	534 (371–696)	669·0 (464·0–869·0)	-3·9 (-7·4–0·4)
Dominican Republic	650 000 (567 000–728 000)	6 120 (5 320–6 810)	3·2 (-0·1–8·0)	72 800 (50 800–94 100)	683·0 (477·0–883·0)	2·4 (-1·4–7·4)
Grenada	6 770 (6 050–7 810)	5 950 (5 320–6 760)	-1·5 (-5·0–1·9)	757 (518–975)	664·0 (456·0–850·0)	-2·0 (-5·5–1·4)
Guyana	44 300 (38 800–50 500)	5 930 (5 240–6 740)	-0·9 (-4·9–3·2)	4 910 (3 360–6 300)	654·0 (449·0–835·0)	-1·3 (-5·5–3·4)

Haiti	620 000 (549 000–706 000)	5 980 (5 280–6 740)	-0·7 (-4·6–2·7)	69 000 (48 500–89 200)	660·0 (466·0–844·0)	-1·0 (-4·9–2·6)
Jamaica	187 000 (168 000–213 000)	6 150 (5 490–6 960)	-0·0 (-3·5–3·2)	21 100 (14 500–26 900)	691·0 (478·0–882·0)	-0·5 (-5·0–3·2)
Puerto Rico	292 000 (259 000–326 000)	5 930 (5 260–6 680)	0·1 (-4·3–3·3)	32 200 (22 300–41 800)	663·0 (459·0–857·0)	-0·6 (-5·5–3·7)
Saint Kitts and Nevis	4 060 (3 560–4 670)	5 850 (5 200–6 650)	-2·7 (-5·5–0·6)	455 (317–602)	653·0 (456·0–845·0)	-2·8 (-6·1–0·4)
Saint Lucia	12 700 (11 300–14 400)	6 060 (5 380–6 870)	-4·4 (-8·3–-0·6)	1 410 (970–1 840)	675·0 (463·0–872·0)	-4·6 (-8·6–-0·6)
Saint Vincent and the Grenadines	7 520 (6 650–8 580)	5 830 (5 170–6 640)	-1·9 (-5·5–2·1)	839 (581–1 100)	650·0 (450·0–838·0)	-2·5 (-6·1–2·5)
Suriname	37 400 (33 200–42 800)	6 080 (5 420–6 890)	2·5 (-2·0–6·6)	4 170 (2 910–5 420)	676·0 (473·0–871·0)	1·7 (-2·7–6·3)
Trinidad and Tobago	101 000 (88 900–113 000)	6 000 (5 270–6 770)	-0·8 (-4·9–2·6)	11 300 (7 860–14 600)	669·0 (466·0–865·0)	-1·2 (-5·8–2·8)
United States Virgin Islands	8 330 (7 260–9 350)	6 040 (5 340–6 790)	0·7 (-3·7–4·6)	921 (648–1 220)	673·0 (474·0–879·0)	-0·3 (-4·7–4·6)
Colombia	4 290 000 (3 850 000–4 800 000)	8 180 (7 330–9 150)	1·5 (-2·9–6·6)	483 000 (331 000–612 000)	920·0 (630·0–1160·0)	1·6 (-3·1–8·5)
Costa Rica	392 000 (350 000–445 000)	7 500 (6 720–8 460)	-2·2 (-6·4–1·8)	43 800 (30 600–57 100)	838·0 (587·0–1090·0)	-2·8 (-6·9–1·5)
El Salvador	481 000 (429 000–543 000)	7 700 (6 860–8 670)	2·9 (-3·0–6·7)	53 700 (37 300–69 800)	861·0 (600·0–1120·0)	2·8 (-3·6–7·0)
Guatemala	1 330 000 (1 170 000–1 500 000)	8 520 (7 540–9 580)	-3·2 (-7·5–1·6)	148 000 (103 000–192 000)	944·0 (665·0–1230·0)	-3·5 (-7·4–1·6)
Honduras	656 000 (580 000–751 000)	7 680 (6 830–8 740)	2·1 (-1·7–6·6)	73 500 (51 300–95 800)	855·0 (595·0–1120·0)	1·4 (-3·6–5·7)

Mexico	9 310 000 (8 250 000–10 600 000)	7 180 (6 380–8 150)	2·4 (1·0–3·6)	1 040 000 (722 000–1 340 000)	799·0 (554·0–1020·0)	2·6 (1·1–4·1)
Nicaragua	461 000 (405 000–528 000)	7 700 (6 800–8 740)	-0·6 (-4·7–4·1)	51 600 (36 500–67 000)	858·0 (606·0–1110·0)	-1·0 (-5·4–4·5)
Panama	320 000 (284 000–361 000)	7 420 (6 600–8 380)	1·1 (-3·1–5·2)	35 800 (25 100–46 500)	831·0 (583·0–1080·0)	0·5 (-3·6–5·4)
Venezuela (Bolivarian Republic of)	2 050 000 (1 820 000–2 310 000)	6 850 (6 090–7 680)	-3·9 (-7·9–-0·1)	229 000 (158 000–301 000)	767·0 (527·0–1000·0)	-4·2 (-8·4–0·2)
Brazil	22 300 000 (19 700 000–25 200 000)	9 230 (8 170–10 400)	3·2 (2·0–4·4)	2 470 000 (1 710 000–3 180 000)	1020·0 (707·0–1310·0)	3·0 (1·6–4·3)
Paraguay	511 000 (449 000–573 000)	7 620 (6 750–8 530)	3·9 (-1·2–8·5)	57 100 (39 800–73 500)	850·0 (590·0–1090·0)	3·2 (-1·4–7·8)
Afghanistan	2 240 000 (1 950 000–2 550 000)	8 520 (7 610–9 640)	0·8 (-2·4–4·0)	248 000 (171 000–330 000)	931·0 (654·0–1200·0)	-0·1 (-3·9–3·3)
Algeria	3 440 000 (3 050 000–3 900 000)	8 330 (7 420–9 360)	-1·1 (-6·0–3·1)	385 000 (273 000–505 000)	927·0 (650·0–1200·0)	-1·4 (-6·8–3·4)
Bahrain	129 000 (112 000–148 000)	8 010 (7 160–9 020)	-1·1 (-4·5–2·8)	14 600 (10 300–19 400)	887·0 (605·0–1130·0)	-1·8 (-5·6–2·2)
Egypt	7 610 000 (6 760 000–8 610 000)	8 630 (7 700–9 680)	2·4 (-2·2–6·4)	853 000 (597 000–1 100 000)	960·0 (666·0–1230·0)	1·9 (-2·8–6·4)
Iran (Islamic Republic of)	8 380 000 (7 450 000–9 530 000)	9 520 (8 470–10 700)	-5·3 (-6·2–-4·4)	934 000 (647 000–1 220 000)	1050·0 (729·0–1350·0)	-5·7 (-6·7–-4·7)
Iraq	3 040 000 (2 700 000–3 500 000)	8 370 (7 450–9 470)	-1·1 (-5·4–2·7)	337 000 (233 000–433 000)	919·0 (634·0–1180·0)	-2·1 (-5·7–2·0)
Jordan	894 000 (780 000–1 020 000)	8 430 (7 460–9 440)	-1·1 (-5·3–2·4)	100 000 (70 500–130 000)	936·0 (658·0–1210·0)	-1·7 (-6·4–2·0)

Kuwait	406 000 (353 000–472 000)	8 480 (7 620–9 560)	4·9 (0·2–8·7)	45 900 (31 900–61 400)	942·0 (652·0–1220·0)	4·0 (-0·6–8·6)
Lebanon	456 000 (406 000–521 000)	8 470 (7 520–9 630)	1·2 (-3·3–5·0)	50 400 (34 700–65 400)	935·0 (645·0–1210·0)	0·6 (-4·5–4·3)
Libya	583 000 (513 000–677 000)	8 330 (7 350–9 460)	0·4 (-3·1–4·4)	65 000 (44 800–83 500)	921·0 (629·0–1170·0)	-0·8 (-4·7–3·8)
Morocco	3 470 000 (3 080 000–3 960 000)	9 580 (8 610–10 800)	-1·6 (-6·1–3·6)	386 000 (269 000–498 000)	1060·0 (736·0–1350·0)	-2·6 (-7·7–2·3)
Oman	332 000 (290 000–383 000)	7 820 (6 960–8 740)	-2·8 (-6·1–1·0)	37 900 (26 300–50 100)	872·0 (610·0–1110·0)	-2·7 (-6·8–1·2)
Palestine	333 000 (293 000–378 000)	8 390 (7 440–9 400)	-3·1 (-6·2–1·0)	37 100 (25 300–48 400)	927·0 (637·0–1190·0)	-3·8 (-7·2–0·1)
Qatar	228 000 (201 000–266 000)	7 730 (6 870–8 750)	-0·2 (-3·4–3·1)	26 000 (17 900–35 000)	857·0 (593·0–1100·0)	-0·9 (-4·3–2·9)
Saudi Arabia	2 950 000 (2 570 000–3 390 000)	8 110 (7 270–9 140)	2·4 (-0·7–6·2)	332 000 (235 000–440 000)	897·0 (635·0–1150·0)	2·0 (-1·1–5·9)
Sudan	2 740 000 (2 420 000–3 110 000)	8 510 (7 480–9 580)	-0·4 (-3·8–3·7)	306 000 (210 000–398 000)	944·0 (650·0–1210·0)	-0·7 (-4·4–3·3)
Syrian Arab Republic	1 220 000 (1 060 000–1 380 000)	8 610 (7 650–9 690)	-0·8 (-4·0–2·5)	136 000 (92 800–176 000)	955·0 (654·0–1220·0)	-1·5 (-5·1–2·7)
Tunisia	1 090 000 (959 000–1 230 000)	8 550 (7 550–9 620)	2·1 (-2·4–6·0)	122 000 (85 400–159 000)	949·0 (661·0–1230·0)	1·3 (-3·4–5·4)
Türkiye	8 130 000 (7 220 000–9 220 000)	8 840 (7 880–9 950)	-2·5 (-6·9–3·2)	910 000 (620 000–1 180 000)	987·0 (673·0–1270·0)	-3·0 (-7·2–3·4)
United Arab Emirates	742 000 (620 000–886 000)	7 260 (6 450–8 120)	2·9 (-1·8–7·5)	84 800 (61 800–115 000)	809·0 (573·0–1050·0)	2·9 (-2·3–8·0)
Yemen	2 040 000 (1 800 000–2 310 000)	8 500 (7 600–9 420)	-3·9 (-7·5–-0·6)	226 000 (155 000–292 000)	937·0 (644·0–1210·0)	-4·0 (-7·8–0·2)

Bangladesh	13 900 000<br>(12 200 000–15 700 000)	9 000<br>(7 940–10 100)	-7·6<br>(-13·0–-2·7)	1 550 000<br>(1 080 000–2 030 000)	998·0 (700·0–<br>1310·0)	-7·4<br>(-12·8–-2·6)
Bhutan	61 200<br>(53 900–70 400)	8 660<br>(7 620–9 880)	-2·4<br>(-6·6–1·0)	6 880<br>(4 720–8 970)	966·0 (669·0–<br>1270·0)	-2·2<br>(-6·6–2·1)
India	87 500 000<br>(77 000 000–99 600 000)	6 540<br>(5 820–7 400)	-13·3<br>(-14·2–-12·3)	9 690 000<br>(6 750 000–12 400 000)	719·0 (502·0–<br>927·0)	-12·8<br>(-13·9–-11·8)
Nepal	2 860 000<br>(2 520 000–3 240 000)	10 500<br>(9 230–11 800)	-9·0<br>(-14·9–-3·2)	318 000<br>(218 000–416 000)	1160·0 (797·0–<br>1520·0)	-8·5<br>(-14·2–-3·3)
Pakistan	12 900 000<br>(11 300 000–15 200 000)	7 810<br>(6 830–9 060)	10·2 (6·8–<br>13·5)	1 440 000<br>(1 000 000–1 890 000)	860·0 (600·0–<br>1110·0)	9·9 (6·7–<br>13·5)
China	99 300 000<br>(87 900 000–112 000 000)	5 360<br>(4 790–6 040)	-19·4<br>(-20·7–-18·0)	11 200 000<br>(7 790 000–14 700 000)	605·0 (417·0–<br>783·0)	-19·3<br>(-20·8–-17·8)
Democratic People's Republic of Korea	2 150 000<br>(1 890 000–2 440 000)	6 790<br>(5 980–7 630)	-6·0<br>(-10·0–-2·4)	244 000<br>(171 000–321 000)	769·0 (533·0–<br>1010·0)	-5·9<br>(-9·3–-1·9)
Taiwan (Province of China)	2 820 000<br>(2 670 000–2 870 000)	8 240<br>(7 800–8 390)	17·5 (9·1–<br>23·7)	318 000<br>(232 000–414 000)	935·0 (677·0–<br>1210·0)	17·5 (8·8–<br>23·5)
American Samoa	3 400<br>(2 990–3 870)	6 510<br>(5 760–7 370)	-0·7<br>(-5·0–3·4)	378<br>(260–492)	721·0 (496·0–<br>932·0)	-1·9<br>(-5·5–2·5)
Cook Islands	1 440<br>(1 270–1 610)	6 610<br>(5 810–7 450)	2·7<br>(-1·5–6·1)	160<br>(113–209)	737·0 (517·0–<br>948·0)	1·9<br>(-2·1–5·9)
Fiji	53 900<br>(47 500–60 700)	6 250<br>(5 580–6 940)	-3·5<br>(-7·0–0·2)	6 050<br>(4 280–7 920)	696·0 (491·0–<br>904·0)	-4·0<br>(-7·6–0·9)
Guam	11 500<br>(10 300–13 000)	6 280<br>(5 600–7 080)	-1·4<br>(-5·1–2·0)	1 300<br>(903–1 700)	707·0 (494·0–<br>915·0)	-1·9<br>(-5·9–2·0)
Kiribati	6 330<br>(5 600–7 220)	6 770<br>(6 030–7 610)	3·4<br>(-0·9–7·5)	712<br>(492–928)	752·0 (521·0–<br>960·0)	3·6<br>(-0·8–8·4)

Marshall Islands	2 890 (2 530–3 310)	6 050 (5 360–6 820)	-3·3 (-6·7–0·1)	326 (228–427)	672·0 (466·0–865·0)	-3·8 (-7·6–-0·7)
Micronesia (Federated States of)	5 840 (5 070–6 610)	6 630 (5 820–7 450)	0·8 (-3·4–5·3)	658 (454–869)	738·0 (512·0–959·0)	0·4 (-3·9–5·3)
Nauru	492 (431–564)	6 680 (5 920–7 430)	2·2 (-1·1–5·4)	55.7 (38.1–74.4)	743·0 (505·0–960·0)	1·4 (-2·0–5·3)
Niue	125 (111–141)	6 400 (5 730–7 190)	-1·3 (-4·2–2·0)	13.8 (9.61–18.1)	713·0 (494·0–915·0)	-1·9 (-5·0–2·4)
Northern Mariana Islands	3 370 (2 910–3 840)	6 360 (5 610–7 120)	-0·0 (-3·9–4·1)	381 (268–503)	713·0 (497·0–928·0)	-0·5 (-4·1–4·4)
Palau	1 380 (1 200–1 590)	6 240 (5 540–7 040)	-2·3 (-5·9–1·9)	155 (110–202)	694·0 (492·0–886·0)	-3·0 (-6·8–1·5)
Papua New Guinea	467 000 (403 000–539 000)	6 280 (5 480–7 130)	-0·6 (-5·0–4·0)	52 800 (36 800–70 000)	701·0 (488·0–912·0)	-0·4 (-5·0–4·8)
Samoa	11 500 (10 200–13 100)	6 530 (5 840–7 380)	-5·4 (-10·1–-1·9)	1 290 (904–1 690)	728·0 (510·0–952·0)	-5·9 (-10·9–-1·8)
Solomon Islands	31 600 (27 600–36 500)	6 690 (5 920–7 590)	7·5 (3·0–12·4)	3 580 (2 520–4 690)	747·0 (523·0–964·0)	7·1 (2·5–12·9)
Tokelau	88 (77.7–99.9)	6 340 (5 610–7 240)	-2·9 (-6·2–1·5)	9.83 (6.83–12.9)	708·0 (487·0–921·0)	-3·2 (-7·3–1·0)
Tonga	5 750 (5 010–6 490)	6 600 (5 780–7 410)	-2·6 (-6·7–1·8)	645 (447–826)	738·0 (514·0–951·0)	-3·2 (-6·8–1·2)
Tuvalu	724 (633–819)	6 450 (5 660–7 250)	-3·2 (-6·5–1·1)	81.4 (56.8–105)	721·0 (505·0–926·0)	-3·3 (-7·5–1·0)
Vanuatu	16 000 (14 000–18 000)	6 940 (6 160–7 750)	1·2 (-3·5–4·7)	1 800 (1 240–2 340)	778·0 (536·0–998·0)	1·1 (-3·8–4·9)

Cambodia	925 000 (815 000–1 050 000)	6 230 (5 500–7 020)	-7·4 (-10·6–-3·4)	104 000 (71 200–138 000)	696·0 (475·0–914·0)	-6·9 (-11·0–-2·3)
Indonesia	16 300 000 (14 300 000–18 700 000)	6 270 (5 600–7 060)	-2·6 (-4·2–-1·4)	1 850 000 (1 290 000–2 410 000)	705·0 (487·0–905·0)	-2·3 (-3·8–-0·9)
Lao People's Democratic Republic	351 000 (308 000–401 000)	5 800 (5 170–6 550)	-5·2 (-8·6–-2·4)	39 900 (27 100–52 700)	653·0 (443·0–852·0)	-4·6 (-8·9–-0·6)
Malaysia	1 740 000 (1 540 000–1 980 000)	5 550 (4 940–6 290)	-2·4 (-6·5–1·6)	197 000 (137 000–261 000)	622·0 (432·0–824·0)	-2·5 (-7·1–2·7)
Maldives	24 900 (21 900–29 100)	5 050 (4 460–5 730)	-4·9 (-8·4–-1·3)	2 850 (2 010–3 750)	568·0 (391·0–729·0)	-4·7 (-8·8–-0·6)
Mauritius	94 000 (82 200–106 000)	5 800 (5 130–6 600)	-5·5 (-9·5–-0·9)	10 400 (7 280–13 700)	643·0 (447·0–833·0)	-6·0 (-10·3–-1·0)
Myanmar	2 700 000 (2 400 000–3 100 000)	5 090 (4 530–5 780)	0·7 (-3·4–5·1)	305 000 (217 000–399 000)	569·0 (405·0–738·0)	1·1 (-3·9–5·7)
Philippines	5 940 000 (5 210 000–6 750 000)	6 080 (5 410–6 820)	-2·4 (-3·2–-1·6)	670 000 (464 000–867 000)	680·0 (472·0–879·0)	-2·3 (-3·1–-1·5)
Seychelles	6 240 (5 510–7 030)	5 360 (4 770–5 970)	-5·5 (-9·3–-2·1)	703 (496–916)	600·0 (423·0–772·0)	-6·0 (-9·2–-2·0)
Sri Lanka	1 430 000 (1 260 000–1 630 000)	5 750 (5 100–6 570)	-0·2 (-4·6–3·9)	160 000 (111 000–213 000)	641·0 (443·0–851·0)	-0·5 (-4·8–3·5)
Thailand	4 890 000 (4 300 000–5 580 000)	5 200 (4 600–5 840)	3·8 (-1·1–10·3)	549 000 (381 000–716 000)	584·0 (405·0–757·0)	4·5 (-1·0–11·3)
Timor-Leste	55 000 (48 400–62 100)	5 380 (4 740–6 060)	-4·7 (-7·8–-0·1)	6 190 (4 360–7 930)	602·0 (425·0–784·0)	-4·3 (-8·1–-0·2)
Viet Nam	6 190 000 (5 420 000–7 150 000)	5 920 (5 220–6 730)	-1·3 (-4·9–1·7)	704 000 (485 000–918 000)	668·0 (460·0–862·0)	-0·9 (-5·0–3·0)

Angola	1 420 000 (1 240 000–1 610 000)	7 480 (6 640–8 330)	-4·2 (-8·7–-0·6)	158 000 (108 000–206 000)	826·0 (568·0–1090·0)	-4·3 (-8·4–-1·0)
Central African Republic	266 000 (233 000–304 000)	7 570 (6 680–8 480)	-1·6 (-5·5–3·4)	29 500 (20 400–38 000)	829·0 (575·0–1070·0)	-1·5 (-5·8–4·2)
Congo	285 000 (252 000–328 000)	7 110 (6 370–7 980)	-1·3 (-5·3–2·5)	31 900 (22 400–41 300)	786·0 (547·0–1010·0)	-1·3 (-5·4–2·8)
Democratic Republic of the Congo	4 350 000 (3 870 000–4 980 000)	7 520 (6 710–8 440)	-3·5 (-7·2–-0·2)	485 000 (340 000–629 000)	828·0 (584·0–1070·0)	-2·8 (-7·3–0·8)
Equatorial Guinea	66 800 (58 700–76 200)	7 270 (6 460–8 170)	-5·5 (-9·2–-2·0)	7 440 (5 140–9 550)	800·0 (555·0–1030·0)	-4·8 (-8·7–-0·4)
Gabon	102 000 (89 400–115 000)	7 050 (6 260–7 920)	-0·4 (-3·7–3·2)	11 300 (7 860–14 800)	778·0 (541·0–1010·0)	-0·7 (-4·4–2·9)
Burundi	567 000 (498 000–647 000)	7 500 (6 740–8 390)	-6·0 (-10·4–-2·2)	63 500 (43 900–83 500)	833·0 (580·0–1080·0)	-6·0 (-10·0–-1·9)
Comoros	43 200 (38 100–48 500)	7 110 (6 280–7 960)	0·3 (-3·7–4·8)	4 860 (3 380–6 320)	795·0 (551·0–1040·0)	0·8 (-3·4–5·8)
Djibouti	63 500 (56 000–72 300)	6 700 (5 930–7 470)	-5·4 (-8·8–-1·2)	7 190 (4 980–9 490)	749·0 (517·0–969·0)	-5·3 (-9·0–-1·1)
Eritrea	304 000 (268 000–343 000)	6 790 (6 090–7 570)	0·6 (-3·7–4·6)	34 200 (24 200–44 100)	752·0 (530·0–978·0)	1·3 (-3·3–5·6)
Ethiopia	5 120 000 (4 480 000–5 840 000)	7 440 (6 650–8 310)	-7·3 (-9·0–-5·3)	575 000 (397 000–746 000)	826·0 (574·0–1060·0)	-6·4 (-8·0–-4·3)
Kenya	2 890 000 (2 540 000–3 290 000)	8 110 (7 230–9 030)	-2·4 (-3·1–-1·7)	324 000 (223 000–419 000)	901·0 (623·0–1170·0)	-2·3 (-3·1–-1·6)
Madagascar	1 350 000 (1 190 000–1 520 000)	7 500 (6 700–8 350)	-6·7 (-10·3–-3·4)	152 000 (105 000–198 000)	835·0 (580·0–1090·0)	-6·3 (-10·1–-2·4)

Malawi	887 000 (778 000–1 010 000)	7 460 (6 710–8 370)	-2·1 (-5·7–1·8)	99 500 (68 100–129 000)	829·0 (577·0–1080·0)	-1·3 (-4·9–2·6)
Mozambique	1 430 000 (1 260 000–1 620 000)	8 020 (7 150–8 960)	-0·6 (-5·0–3·0)	158 000 (112 000–202 000)	877·0 (622·0–1130·0)	-0·6 (-5·2–3·4)
Rwanda	746 000 (657 000–835 000)	8 120 (7 250–8 980)	-2·4 (-5·5–1·5)	83 700 (58 200–111 000)	902·0 (626·0–1190·0)	-2·3 (-5·7–2·5)
Somalia	910 000 (802 000–1 030 000)	7 670 (6 790–8 600)	0·9 (-2·8–3·9)	102 000 (72 800–134 000)	849·0 (603·0–1100·0)	1·0 (-2·9–4·2)
South Sudan	431 000 (376 000–489 000)	7 330 (6 520–8 260)	-0·7 (-4·2–3·2)	47 800 (33 400–61 300)	804·0 (562·0–1030·0)	-0·6 (-4·8–3·3)
Uganda	1 880 000 (1 660 000–2 120 000)	7 730 (6 940–8 630)	-2·9 (-6·4–0·7)	211 000 (147 000–272 000)	859·0 (601·0–1130·0)	-1·8 (-5·4–2·1)
United Republic of Tanzania	2 810 000 (2 490 000–3 190 000)	7 470 (6 640–8 390)	-3·3 (-6·2–-0·5)	315 000 (218 000–412 000)	830·0 (575·0–1080·0)	-2·2 (-5·5–1·0)
Zambia	816 000 (725 000–940 000)	6 970 (6 250–7 900)	9·6 (4·6–14·7)	91 300 (63 800–117 000)	772·0 (541·0–1000·0)	9·6 (4·4–14·7)
Botswana	126 000 (111 000–144 000)	6 470 (5 750–7 290)	-0·4 (-4·5–3·4)	14 000 (9 900–18 200)	710·0 (497·0–906·0)	-1·7 (-5·3–3·1)
Eswatini	47 800 (42 700–53 900)	5 810 (5 240–6 450)	-1·2 (-4·8–2·5)	5 260 (3 720–6 700)	632·0 (448·0–820·0)	-3·2 (-7·9–1·0)
Lesotho	105 000 (93 000–119 000)	6 320 (5 590–7 100)	-2·8 (-5·8–0·9)	11 600 (8 110–15 100)	688·0 (479·0–888·0)	-4·7 (-8·0–-1·4)
Namibia	126 000 (110 000–143 000)	6 760 (5 890–7 600)	0·2 (-4·1–4·4)	14 100 (9 690–18 200)	748·0 (517·0–963·0)	-0·2 (-4·3–4·2)
South Africa	3 360 000 (2 970 000–3 810 000)	6 350 (5 670–7 140)	-7·5 (-8·8–-6·2)	371 000 (257 000–481 000)	697·0 (484·0–907·0)	-8·4 (-9·9–-6·2)
Zimbabwe	758 000 (667 000–866 000)	7 350 (6 530–8 340)	7·2 (3·2–11·2)	84 600 (58 000–110 000)	811·0 (553·0–1070·0)	6·4 (2·1–10·7)

Benin	558 000 (492 000–640 000)	6 950 (6 140–7 880)	-4·2 (-8·0–-0·6)	62 600 (43 400–82 400)	774·0 (539·0–1010·0)	-3·5 (-7·6–0·4)
Burkina Faso	1 020 000 (885 000–1 160 000)	7 020 (6 220–7 870)	-2·9 (-6·3–0·5)	115 000 (79 900–149 000)	782·0 (543·0–1020·0)	-2·1 (-5·9–2·8)
Cabo Verde	33 600 (29 800–38 200)	6 410 (5 710–7 220)	-6·5 (-10·3–-3·8)	3 770 (2 620–5 000)	716·0 (494·0–939·0)	-7·0 (-10·4–-3·9)
Cameroon	1 430 000 (1 240 000–1 620 000)	7 170 (6 330–8 060)	-4·9 (-8·4–-1·0)	160 000 (110 000–212 000)	799·0 (554·0–1050·0)	-4·5 (-8·7–-0·2)
Chad	720 000 (637 000–829 000)	7 620 (6 680–8 680)	-8·2 (-12·8–-2·4)	80 500 (56 000–104 000)	845·0 (588·0–1090·0)	-8·3 (-13·5–-2·6)
Côte d'Ivoire	1 290 000 (1 130 000–1 480 000)	7 140 (6 380–8 040)	-3·4 (-6·7–-0·5)	145 000 (100 000–192 000)	794·0 (551·0–1040·0)	-2·1 (-5·8–1·6)
Gambia	98 500 (87 100–111 000)	6 480 (5 760–7 250)	-2·7 (-6·3–1·6)	11 000 (7 680–14 200)	719·0 (504·0–936·0)	-3·1 (-6·9–1·2)
Ghana	1 520 000 (1 360 000–1 700 000)	6 240 (5 680–6 910)	-5·3 (-8·6–-1·4)	171 000 (120 000–222 000)	698·0 (490·0–915·0)	-4·9 (-8·7–-0·8)
Guinea	587 000 (518 000–664 000)	7 130 (6 300–8 020)	-1·1 (-4·8–2·8)	65 900 (45 300–86 700)	794·0 (547·0–1050·0)	-0·8 (-4·8–3·4)
Guinea-Bissau	84 100 (73 900–95 900)	6 750 (5 990–7 500)	-1·9 (-5·3–1·8)	9 400 (6 630–12 300)	747·0 (520·0–974·0)	-1·9 (-5·9–1·7)
Liberia	231 000 (202 000–264 000)	6 730 (5 930–7 500)	-3·0 (-6·9–2·1)	25 500 (17 700–33 300)	735·0 (515·0–955·0)	-2·9 (-7·1–3·1)
Mali	852 000 (747 000–962 000)	6 290 (5 550–7 060)	3·3 (-1·8–9·3)	95 200 (67 400–121 000)	697·0 (495·0–890·0)	3·5 (-2·9–9·9)
Mauritania	182 000 (160 000–207 000)	6 240 (5 550–7 060)	-0·5 (-5·0–3·6)	20 500 (14 100–26 500)	699·0 (478·0–908·0)	-0·3 (-5·1–4·2)
Niger	923 000 (804 000–1 050 000)	7 070 (6 220–7 980)	3·1 (-1·2–7·8)	104 000 (71 600–135 000)	790·0 (546·0–1030·0)	3·4 (-0·8–8·2)

Nigeria	9 870 000 (8 620 000–11 200 000)	6 970 (6 200–7 800)	-1·8 (-2·5–-0·9)	1 110 000 (764 000–1 430 000)	775·0 (538·0–998·0)	-1·3 (-2·2–-0·2)
Sao Tome and Principe	9 500 (8 330–10 800)	6 010 (5 350–6 740)	-3·1 (-6·2–-0·2)	1 070 (742–1 390)	672·0 (466·0–862·0)	-3·5 (-7·2–-0·1)
Senegal	677 000 (599 000–766 000)	6 270 (5 620–6 990)	-3·9 (-8·0–0·7)	75 700 (53 200–97 800)	697·0 (488·0–905·0)	-3·7 (-8·0–1·2)
Sierra Leone	388 000 (345 000–439 000)	6 760 (5 970–7 590)	-5·7 (-9·6–-1·5)	43 400 (30 300–56 900)	751·0 (525·0–977·0)	-5·5 (-9·2–-1·3)
Togo	392 000 (349 000–449 000)	6 840 (6 050–7 680)	-5·4 (-9·3–-1·4)	44 200 (30 100–57 500)	763·0 (523·0–995·0)	-5·0 (-8·7–-0·5)

Note: region/country numbers do not sum to the global prevalence due to rounding.

Data input sources

Administrative Department of Science, Technology, and Innovation (Colombia), Center for Development Projects, Pontifical Xavierian University, Ministry of Social Protection (Colombia), Specialized Information Systems. Colombia National Health Survey 2007-2008.

Federal Statistical Office (Switzerland), Swiss National Science Foundation, University of Neuchatel. Switzerland Household Panel Survey 1999-2000. Lausanne, Switzerland: Swiss Foundation for Research in Social Sciences.

Australian Bureau of Statistics. Australia National Health Survey 1995. Canberra, Australia: Australian Bureau of Statistics.

Department of Health and Ageing (Australia), World Health Organization (WHO). Australia World Health Survey 2003. Geneva, Switzerland: World Health Organization (WHO), 2005.

World Health Organization (WHO). Bangladesh World Health Survey 2003. Geneva, Switzerland: World Health Organization (WHO), 2005.

World Health Organization (WHO). Bosnia and Herzegovina World Health Survey 2003. Geneva, Switzerland: World Health Organization (WHO), 2005.

Center for Scientific and Technological Information, Oswaldo Cruz Foundation and World Health Organization (WHO). Brazil World Health Survey 2003. Geneva, Switzerland: World Health Organization (WHO), 2005.

World Health Organization (WHO). Denmark World Health Survey 2003. Geneva, Switzerland: World Health Organization (WHO), 2005.

World Health Organization (WHO). Burkina Faso World Health Survey 2002-2003. Geneva, Switzerland: World Health Organization (WHO), 2005.

World Health Organization (WHO). Chad World Health Survey 2003. Geneva, Switzerland: World Health Organization (WHO), 2005.

World Health Organization (WHO). China World Health Survey 2002. Geneva, Switzerland: World Health Organization (WHO), 2005.

World Health Organization (WHO). Comoros World Health Survey 2003. Geneva, Switzerland: World Health Organization (WHO), 2005.

World Health Organization (WHO). Congo World Health Survey 2003. Geneva, Switzerland: World Health Organization (WHO), 2005.

World Health Organization (WHO). Côte d'Ivoire World Health Survey 2003. Geneva, Switzerland: World Health Organization (WHO), 2005.

World Health Organization (WHO). Croatia World Health Survey 2003. Geneva, Switzerland: World Health Organization (WHO), 2005.

World Health Organization (WHO). Czech Republic World Health Survey 2002-2003. Geneva, Switzerland: World Health Organization (WHO), 2005.

World Health Organization (WHO). Dominican Republic World Health Survey 2003. Geneva, Switzerland: World Health Organization (WHO), 2005.

World Health Organization (WHO). Ecuador World Health Survey 2003. Geneva, Switzerland: World Health Organization (WHO), 2005.

World Health Organization (WHO). Estonia World Health Survey 2003. Geneva, Switzerland: World Health Organization (WHO), 2005.

World Health Organization (WHO). Ethiopia World Health Survey 2003. Geneva, Switzerland: World Health Organization (WHO), 2005.

World Health Organization (WHO). Finland World Health Survey 2004. Geneva, Switzerland: World Health Organization (WHO), 2005.

World Health Organization (WHO). France World Health Survey 2003. Geneva, Switzerland: World Health Organization (WHO), 2005.

World Health Organization (WHO). Georgia World Health Survey 2003. Geneva, Switzerland: World Health Organization (WHO), 2005.

World Health Organization (WHO). Germany World Health Survey 2004. Geneva, Switzerland: World Health Organization (WHO), 2005.

World Health Organization (WHO). Ghana World Health Survey 2003. Geneva, Switzerland: World Health Organization (WHO), 2005.

World Health Organization (WHO). Greece World Health Survey 2003. Geneva, Switzerland: World Health Organization (WHO), 2005.

World Health Organization (WHO). Guatemala World Health Survey 2003. Geneva, Switzerland: World Health Organization (WHO), 2005.

World Health Organization (WHO). Hungary World Health Survey 2003. Geneva, Switzerland: World Health Organization (WHO), 2005.

International Institute for Population Sciences (India), World Health Organization (WHO). India World Health Survey 2003. Geneva, Switzerland:

World Health Organization (WHO), 2005.

World Health Organization (WHO). Israel World Health Survey 2003. Geneva, Switzerland: World Health Organization (WHO), 2005.

World Health Organization (WHO). Kazakhstan World Health Survey 2002-2003. Geneva, Switzerland: World Health Organization (WHO), 2005.

World Health Organization (WHO). Kenya World Health Survey 2004. Geneva, Switzerland: World Health Organization (WHO), 2005.

World Health Organization (WHO). Laos World Health Survey 2003.

World Health Organization (WHO). Malawi World Health Survey 2003. Geneva, Switzerland: World Health Organization (WHO), 2005.

World Health Organization (WHO). Malaysia World Health Survey 2003. Geneva, Switzerland: World Health Organization (WHO), 2005.

World Health Organization (WHO). Mali World Health Survey 2003. Geneva, Switzerland: World Health Organization (WHO), 2005.

World Health Organization (WHO). Mauritania World Health Survey 2003. Geneva, Switzerland: World Health Organization (WHO), 2005.

World Health Organization (WHO). Mauritius World Health Survey 2003. Geneva, Switzerland: World Health Organization (WHO), 2005.

World Health Organization (WHO). Mexico World Health Survey 2002-2003. Geneva, Switzerland: World Health Organization (WHO), 2005.

World Health Organization (WHO). Morocco World Health Survey 2003. Geneva, Switzerland: World Health Organization (WHO), 2005.

World Health Organization (WHO). Myanmar World Health Survey 2003. Geneva, Switzerland: World Health Organization (WHO), 2005.

World Health Organization (WHO). Namibia World Health Survey 2003. Geneva, Switzerland: World Health Organization (WHO), 2005.

World Health Organization (WHO). Nepal World Health Survey 2003. Geneva, Switzerland: World Health Organization (WHO), 2005.

World Health Organization (WHO). Norway World Health Survey 2003. Geneva, Switzerland: World Health Organization (WHO), 2005.

World Health Organization (WHO). Pakistan World Health Survey 2003-2004. Geneva, Switzerland: World Health Organization (WHO), 2005.

World Health Organization (WHO). Paraguay World Health Survey 2002-2003. Geneva, Switzerland: World Health Organization (WHO), 2005.

World Health Organization (WHO). Philippines World Health Survey 2003. Geneva, Switzerland: World Health Organization (WHO), 2005.

World Health Organization (WHO). Portugal World Health Survey 2003. Geneva, Switzerland: World Health Organization (WHO), 2006.

World Health Organization (WHO). Russia World Health Survey 2003. Geneva, Switzerland: World Health Organization (WHO), 2005.

World Health Organization (WHO). Senegal World Health Survey 2003. Geneva, Switzerland: World Health Organization (WHO), 2005.

World Health Organization (WHO). Slovakia World Health Survey 2003. Geneva, Switzerland: World Health Organization (WHO), 2005.

World Health Organization (WHO). Slovenia World Health Survey 2003. Geneva, Switzerland: World Health Organization (WHO), 2005.

World Health Organization (WHO). South Africa World Health Survey 2002-2003. Geneva, Switzerland: World Health Organization (WHO), 2005.

World Health Organization (WHO). Spain World Health Survey 2002-2003. Geneva, Switzerland: World Health Organization (WHO), 2005.

World Health Organization (WHO). Sri Lanka World Health Survey 2003. Geneva, Switzerland: World Health Organization (WHO), 2005.

World Health Organization (WHO). Swaziland World Health Survey 2003. Geneva, Switzerland: World Health Organization (WHO), 2005.

World Health Organization (WHO). Sweden World Health Survey 2003. Geneva, Switzerland: World Health Organization (WHO), 2005.

World Health Organization (WHO). Tunisia World Health Survey 2003. Geneva, Switzerland: World Health Organization (WHO), 2005.

World Health Organization (WHO). Turkey World Health Survey 2003. Geneva, Switzerland: World Health Organization (WHO), 2005.

World Health Organization (WHO). United Arab Emirates World Health Survey 2003. Geneva, Switzerland: World Health Organization (WHO), 2005.

World Health Organization (WHO). United Kingdom World Health Survey 2004. Geneva, Switzerland: World Health Organization (WHO), 2005.

World Health Organization (WHO). Uruguay World Health Survey 2002-2003. Geneva, Switzerland: World Health Organization (WHO), 2005.

World Health Organization (WHO). Vietnam World Health Survey 2002-2003. Geneva, Switzerland: World Health Organization (WHO), 2005.

World Health Organization (WHO). Zambia World Health Survey 2003. Geneva, Switzerland: World Health Organization (WHO), 2005.

World Health Organization (WHO). Zimbabwe World Health Survey 2003. Geneva, Switzerland: World Health Organization (WHO), 2005.

World Health Organization (WHO). Ukraine World Health Survey 2002-2003. Geneva, Switzerland: World Health Organization (WHO), 2005.

Federal Environment Agency (Germany), Federal Institute for Drugs and Medical Devices (Germany), Max Planck Institute of Psychiatry, Robert Koch Institute. Germany National Health Interview and Examination Survey 1997-1999. Berlin, Germany: Robert Koch Institute, 2000.

Ministry of Health (Chile), National Institute of Statistics (Chile). Chile National Quality of Life and Health Survey 2006. Santiago, Chile: Ministry of Health (Chile).

Economic and Social Research Institute (ESRI) (Ireland), Health Promotion Unit, Department of Health and Children (Ireland), National University of Ireland, Galway, Royal College of Surgeons in Ireland (RCSI), University College Cork. Ireland Survey of Lifestyle Attitudes and Nutrition 2007. Dublin, Ireland: Health Promotion Unit, Department of Health and Children (Ireland).

National Center for Health Statistics (NCHS), Centers for Disease Control and Prevention (CDC), US Census Bureau. United States National Health Interview Survey 2002. Hyattsville, United States: National Center for Health Statistics (NCHS), Centers for Disease Control and Prevention (CDC).

National Center for Health Statistics (NCHS), Centers for Disease Control and Prevention (CDC), US Census Bureau. United States National Health Interview Survey 2003. Hyattsville, United States: National Center for Health Statistics (NCHS), Centers for Disease Control and Prevention (CDC).

National Center for Health Statistics (NCHS), Centers for Disease Control and Prevention (CDC), US Census Bureau. United States National Health Interview Survey 2006. Hyattsville, United States: National Center for Health Statistics (NCHS), Centers for Disease Control and Prevention (CDC).

National Center for Health Statistics (NCHS), Centers for Disease Control and Prevention (CDC), US Census Bureau. United States National

Health Interview Survey 2007. Hyattsville, United States: National Center for Health Statistics (NCHS), Centers for Disease Control and Prevention (CDC).

National Center for Health Statistics (NCHS), Centers for Disease Control and Prevention (CDC), US Census Bureau. United States National Health Interview Survey 2008. Hyattsville, United States: National Center for Health Statistics (NCHS), Centers for Disease Control and Prevention (CDC).

National Center for Health Statistics (NCHS), Centers for Disease Control and Prevention (CDC), US Census Bureau. United States National Health Interview Survey 2009. Hyattsville, United States: National Center for Health Statistics (NCHS), Centers for Disease Control and Prevention (CDC).

Central Administration of Statistics (Lebanon), League of Arab States, Ministry of Social Affairs (Lebanon), Pan Arab Project for Family Health (PAPFAM). Lebanon Family Health Survey 2004.

National Center for Health Statistics (NCHS), Centers for Disease Control and Prevention (CDC). United States National Health and Nutrition Examination Survey 2003-2004. Hyattsville, United States: National Center for Health Statistics (NCHS), Centers for Disease Control and Prevention (CDC).

National Center for Health Statistics (NCHS), Centers for Disease Control and Prevention (CDC). United States National Health and Nutrition Examination Survey 2009-2010. Hyattsville, United States: National Center for Health Statistics (NCHS), Centers for Disease Control and Prevention (CDC), 2011.

National Center for Health Statistics (NCHS), Centers for Disease Control and Prevention (CDC). United States National Health and Nutrition Examination Survey 2001-2002. Hyattsville, United States: National Center for Health Statistics (NCHS), Centers for Disease Control and Prevention (CDC).

National Center for Health Statistics (NCHS), Centers for Disease Control and Prevention (CDC). United States National Health and Nutrition Examination Survey 1999-2000. Hyattsville, United States: National Center for Health Statistics (NCHS), Centers for Disease Control and Prevention (CDC).

Federal Statistical Office (Switzerland), Swiss National Science Foundation, University of Neuchatel. Switzerland Household Panel Survey 2000-2001. Lausanne, Switzerland: Swiss Foundation for Research in Social Sciences.

Federal Statistical Office (Switzerland), Swiss National Science Foundation, University of Neuchatel. Switzerland Household Panel Survey 2001-

2002. Lausanne, Switzerland: Swiss Foundation for Research in Social Sciences.

Federal Statistical Office (Switzerland), Swiss National Science Foundation, University of Neuchatel. Switzerland Household Panel Survey 2002-2003. Lausanne, Switzerland: Swiss Foundation for Research in Social Sciences.

Federal Statistical Office (Switzerland), Swiss National Science Foundation, University of Neuchatel. Switzerland Household Panel Survey 2003-2004. Lausanne, Switzerland: Swiss Foundation for Research in Social Sciences.

Federal Statistical Office (Switzerland), Swiss National Science Foundation, University of Neuchatel. Switzerland Household Panel Survey 2004-2005. Lausanne, Switzerland: Swiss Foundation for Research in Social Sciences.

Federal Statistical Office (Switzerland), Swiss National Science Foundation, University of Neuchatel. Switzerland Household Panel Survey 2006-2007. Lausanne, Switzerland: Swiss Foundation for Research in Social Sciences.

Federal Statistical Office (Switzerland), Swiss National Science Foundation, University of Neuchatel. Switzerland Household Panel Survey 2007-2008. Lausanne, Switzerland: Swiss Foundation for Research in Social Sciences.

Swiss Foundation for Research in Social Sciences. Switzerland Household Panel Survey 2008-2009. Lausanne, Switzerland: Swiss Foundation for Research in Social Sciences.

Central Statistical Office (Zambia). Zambia Living Conditions Monitoring Survey 2010.

Ministry of Health (China), National Center for Chronic and Noncommunicable Disease Control and Prevention, Chinese Center for Disease Control and Prevention (CCDC), World Health Organization (WHO). China WHO Study on Global AGEing and Adult Health 2007-2010.

International Institute for Population Sciences (India), World Health Organization (WHO). India WHO Study on Global Ageing and Adult Health 2007. Geneva, Switzerland: World Health Organization (WHO), 2007.

Australian Bureau of Statistics. Australia National Health Survey 2001. Canberra, Australia: Australian Bureau of Statistics.

Australian Bureau of Statistics. Australia National Health Survey 2004-2005.

Australian Bureau of Statistics. Australia National Health Survey 2007-2008. Canberra, Australia: Australian Bureau of Statistics.

Australian Bureau of Statistics. Australia Disability, Ageing, and Carers Survey 2003.

Australian Bureau of Statistics. Australia Disability, Ageing, and Carers Survey 2009.

NatCen Social Research and University College London. Department of Epidemiology and Public Health, Health Survey for England, 2011 [computer file]. Colchester, Essex: UK Data Archive [distributor], April 2013. SN: 7260, http://dx.doi.org/10.5255/UKDA-SN-7260-1

European Commission (2012): Eurobarometer 66.2 (Oct-Nov 2006). TNS OPINION & SOCIAL, Brussels [Producer]. GESIS Data Archive, Cologne. ZA4527 Data file Version 1.0.1, doi:10.4232/1.10981

Ministry of Health (Turkey). Turkey Chronic Diseases and Risk Factors Study 2011.

Bergman S, Herrström P, Högström K, Petersson IF, Svensson B, Jacobsson LT. Chronic musculoskeletal pain, prevalence rates, and sociodemographic associations in a Swedish population study. J Rheumatol. 2001; 28(6): 1369-77.

Demyttenaere K, Bruffaerts R, Lee S, Posada-Villa J, Kovess V, Angermeyer MC, Levinson D, de Girolamo G, Nakane H, Mneimneh Z, Lara C, de Graaf R, Scott KM, Gureje O, Stein DJ, Haro JM, Bromet EJ, Kessler RC, Alonso J, Von Korff M. Mental disorders among persons with chronic back or neck pain: results from the World Mental Health Surveys. Pain. 2007; 129(3): 332-42.

Hakala PT, Rimpelä AH, Saarni LA, Salminen JJ. Frequent computer-related activities increase the risk of neck-shoulder and low back pain in adolescents. Eur J Public Health. 2006; 16(5): 536-41.

Chaiamnuay P, Darmawan J, Muirden KD, Assawatanabodee P. Epidemiology of rheumatic disease in rural Thailand: a WHO-ILAR COPCORD study. Community Oriented Programme for the Control of Rheumatic Disease. J Rheumatol. 1998; 25(7): 1382-7.

Davatchi F, Jamshidi A-R, Banihashemi AT, Gholami J, Forouzanfar MH, Akhlaghi M, Barghamdi M, Noorolahzadeh E, Khabazi A-R, Salesi M, Salari A-H, Karimifar M, Essalat-Manesh K, Hajialiloo M, Soroosh M, Farzad F, Moussavi H-R, Samadi F, Ghaznavi K, Asgharifard H, Zangiabadi A-H, Shahram F, Nadji A, Akbarian M, Gharibdoost F. WHO-ILAR COPCORD Study (Stage 1, Urban Study) in Iran. J Rheumatol. 2008; 35(7): 1384.

Manahan L, Caragay R, Muirden KD, Allander E, Valkenburg HA, Wigley RD. Rheumatic pain in a Philippine village. A WHO-ILAR COPCORD Study. Rheumatol Int. 1985; 5(4): 149-53.

Picavet HSJ, Schouten JSAG. Musculoskeletal pain in the Netherlands: prevalences, consequences and risk groups, the DMC(3)-study. Pain. 2003;

102(1-2): 167-78.

Thomas E, Peat G, Harris L, Wilkie R, Croft PR. The prevalence of pain and pain interference in a general population of older adults: cross-sectional findings from the North Staffordshire Osteoarthritis Project (NorStOP). Pain. 2004; 110(1-2): 361-8.

Urwin M, Symmons D, Allison T, Brammah T, Busby H, Roxby M, Simmons A, Williams G. Estimating the burden of musculoskeletal disorders in the community: the comparative prevalence of symptoms at different anatomical sites, and the relation to social deprivation. Ann Rheum Dis. 1998; 57(11): 649-55.

Al-Awadhi AM, Olusi SO, Moussa M, Shehab D, Al-Zaid N, Al-Herz A, Al-Jarallah K. Musculoskeletal pain, disability and health-seeking behavior in adult Kuwaitis using a validated Arabic version of the WHO-ILAR COPCORD Core Questionnaire. Clin Exp Rheumatol. 2004; 22(2): 177-83.

Cardiel MH, Rojas-Serrano J. Community based study to estimate prevalence, burden of illness and help seeking behavior in rheumatic diseases in Mexico City. A COPCORD study. Clin Exp Rheumatol. 2002; 20(5): 617-24.

Haq SA, Darmawan J, Islam MN, Uddin MZ, Das BB, Rahman F, Chowdhury MAJ, Alam MN, Mahmud TAK, Chowdhury MR, Tahir M. Prevalence of rheumatic diseases and associated outcomes in rural and urban communities in Bangladesh: a COPCORD study. J Rheumatol. 2005; 32(2): 348-53.

Farooqi A, Gibson T. Prevalence of the major rheumatic disorders in the adult population of north Pakistan. Br J Rheumatol. 1998; 37(5): 491-5.

Reyes-Llerena GA, Guibert-Toledano M, Penedo-Coello A, Pérez-Rodríguez A, Baez-Dueñas RM, Charnicharo-Vidal R, Cardiel MH. Community-based study to estimate prevalence and burden of illness of rheumatic diseases in Cuba: a COPCORD study. J Clin Rheumatol. 2009; 15(2): 51-5.

Minh Hoa TT, Darmawan J, Chen SL, Van Hung N, Thi Nhi C, Ngoc An T, Damarwan J, Shun Le C. Prevalence of the rheumatic diseases in urban Vietnam: a WHO-ILAR COPCORD study. J Rheumatol. 2003; 30(10): 2252-6.

Salaffi F, De Angelis R, Grassi W, MArche Pain Prevalence, INvestigation Group (MAPPING) study. Prevalence of musculoskeletal conditions in an Italian population sample: results of a regional community-based study. I. The MAPPING study. Clin Exp Rheumatol. 2005; 23(6): 819-28.

Joshi VL, Chopra A. Is there an urban-rural divide? Population surveys of rheumatic musculoskeletal disorders in the Pune region of India using the COPCORD Bhigwan model. J Rheumatol. 2009; 36(3): 614-22.

Veerapen K, Wigley RD, Valkenburg H. Musculoskeletal pain in Malaysia: a COPCORD survey. J Rheumatol. 2007; 34(1): 207-13.

Ghana Health Service, Ministry of Health (Ghana), University of Ghana, World Health Organization (WHO). Ghana WHO Study on Global AGEing and Adult Health 2007-2008.

National Institute of Public Health (Mexico), World Health Organization (WHO). Mexico WHO Study on Global AGEing and Adult Health 2009-2010. Geneva, Switzerland: World Health Organization (WHO), 2011.

Russian Academy of Medical Science, World Health Organization (WHO). Russia WHO Study on Global AGEing and Adult Health 2007-2010.

Department of Health (South Africa), Human Sciences Research Council, World Health Organization (WHO). South Africa WHO Study on Global AGEing and Adult Health 2007-2008. Geneva, Switzerland: World Health Organization (WHO).

Ministry of Health (Singapore). Singapore National Health Surveillance Survey 2007.

Abegunde KA, Owoaje ET. Health problems and associated risk factors in selected urban and rural elderly population groups of South-West Nigeria. Ann Afr Med. 2013; 12(2): 90-7.

Kjaer P, Wedderkopp N, Korsholm L, Leboeuf-Yde C. Prevalence and tracking of back pain from childhood to adolescence. BMC Musculoskelet Disord. 2011; 98.

Andersson HI, Ejlertsson G, Leden I, Rosenberg C. Chronic pain in a geographically defined general population: studies of differences in age, gender, social class, and pain localization. Clin J Pain. 1993; 9(3): 174-82.

Fabunmi AA, Aba SO, Odunaiya NA. Prevalence of low back pain among peasant farmers in a rural community in South West Nigeria. Afr J Med Med Sci. 2005; 34(3): 259-62.

Korovessis P, Repantis T, Zacharatos S, Baikousis A. Low back pain and sciatica prevalence and intensity reported in a Mediterranean country: ordinal logistic regression analysis. Orthopedics. 2012; 35(12): e1775-1784.

Björnsdóttir S, Jónsson S, Valdimarsdóttir U. Functional limitations and physical symptoms of individuals with chronic pain. Scand J Rheumatol. 2013; 42(1): 59-70.

Fujii T, Matsudaira K. Prevalence of low back pain and factors associated with chronic disabling back pain in Japan. Eur Spine J. 2013; 22(2): 432-8.

Kitahara H, Ye Z, Aoyagi K, Ross PD, Abe Y, Honda S, Kanagae M, Mizukami S, Kusano Y, Tomita M, Shindo H, Osaki M. Associations of vertebral deformities and osteoarthritis with back pain among Japanese women: the Hizen-Oshima study. Osteoporos Int. 2013; 24(3): 907-15.

Pieber K, Stein K, Herceg M, Rieder A, Fialka-Moser V, Dorner T. Determinants of satisfaction with individual health in male and female patients with chronic low back pain. J Rehabil Med. 2012; 44(8): 658-63.

Broom AF, Kirby ER, Sibbritt DW, Adams J, Refshauge KM. Back pain amongst mid-age Australian women: A longitudinal analysis of provider use and self-prescribed treatments. Complement Ther Med. 2012; 20(5): 275-82.

Cho NH, Jung YO, Lim SH, Chung C-K, Kim HA. The prevalence and risk factors of low back pain in rural community residents of Korea. Spine. 2012; 37(24): 2001-10.

Rodríguez-Oviedo P, Ruano-Ravina A, Pérez-Ríos M, García FB, Gómez-Fernández D, Fernández-Alonso A, Carreira-Núñez I, García-Pacios P, Turiso J. School children's backpacks, back pain and back pathologies. Arch Dis Child. 2012; 97(8): 730-2.

Turk Z, Vauhnik R, Miceti∱á-Turk D. Prevalence of Nonspecific Low Back Pain in Schoolchildren in North-Eastern Slovenia. Coll Antropol. 2011; 35(4): 1031-5.

O'Sullivan PB, Beales DJ, Smith AJ, Straker LM. Low back pain in 17 year olds has substantial impact and represents an important public health disorder: a cross-sectional study. BMC Public Health. 2012; 12(1): 100.

Ono R, Yamazaki S, Takegami M, Otani K, Sekiguchi M, Onishi Y, Hayashino Y, Kikuchi S, Konno S, Fukuhara S. Gender difference in association between low back pain and metabolic syndrome: locomotive syndrome and health outcome in Aizu cohort study (LOHAS). Spine. 2012; 37(13): 1130-7.

Widanarko B, Legg S, Stevenson M, Devereux J, Eng A, 't Mannetje A, Cheng S, Pearce N. Prevalence and work-related risk factors for reduced activities and absenteeism due to low back symptoms. Appl Ergon. 2012; 43(4): 727-37.

Onofrio AC, da Silva MC, Domingues MR, Rombaldi AJ. Acute low back pain in high school adolescents in Southern Brazil: prevalence and associated factors. Eur Spine J. 2012; 21(7): 1234-40.

Balagué F, Ferrer M, Rajmil L, Pont Acuña A, Pellisé F, Cedraschi C. Assessing the association between low back pain, quality of life, and life events as reported by schoolchildren in a population-based study. Eur J Pediatr. 2012; 171(3): 507-14.

Macfarlane GJ, Beasley M, Jones EA, Prescott GJ, Docking R, Keeley P, McBeth J, Jones GT. The prevalence and management of low back pain across adulthood: Results from a population-based cross-sectional study (the MUSICIAN study). Pain. 2012; 153(1): 27-32.

De Vitta A, Martinez MG, Piza NT, Simeão SF de AP, Ferreira NP. [Prevalence of lower back pain and associated factors in students]. Cad Saude Publica. 2011; 27(8): 1520-8.

Docking RE, Fleming J, Brayne C, Zhao J, Macfarlane GJ, Jones GT, Cambridge City over-75s Cohort Study collaboration. Epidemiology of back pain in older adults: prevalence and risk factors for back pain onset. Rheumatology (Oxford). 2011; 50(9): 1645-53.

Ayanniyi O, Mbada CE, Muolokwu CA. Prevalence and Profile of Back Pain in Nigerian Adolescents. Med Princ Pract. 2011; 20(4): 368-73.

Erne C, Elfering A. Low back pain at school: unique risk deriving from unsatisfactory grade in maths and school-type recommendation. Eur Spine J. 2011; 20(12): 2126-33.

Ferreira GD, Silva MC, Rombaldi AJ, Wrege ED, Siqueira FV, Hallal PC. Prevalence and associated factors of back pain in adults from southern Brazil: a population-based study. Rev Bras Fisioter. 2011; 15(1): 31-6.

Kolb E, Canjuga M, Bauer GF, Läubli T. Course of back pain across 5 years: a retrospective cohort study in the general population of Switzerland. Spine. 2011; 36(4): E268-273.

Yao W, Mai X, Luo C, Ai F, Chen Q. A cross-sectional survey of nonspecific low back pain among 2083 schoolchildren in China. Spine. 2011; 36(22): 1885-90.

Van Gessel H, Gaßmann J, Kröner-Herwig B. Children in Pain: Recurrent Back Pain, Abdominal Pain, and Headache in Children and Adolescents in a Four-Year-Period. J Pediatr. 2011; 158(6): 977-983e2.

Peláez-Ballestas I, Flores-Camacho R, Rodriguez-Amado J, Sanin LH, Valerio JE, Navarro-Zarza E, Flores D, Rivas LL, Casasola-Vargas J, Burgos-Vargas R. Prevalence of back pain in the community. A COPCORD-based study in the Mexican population. J Rheumatol Suppl. 2011; 26-30.

Fernández-de-las-Peñas C, Hernández-Barrera V, Alonso-Blanco C, Palacios-Ceña D, Carrasco-Garrido P, Jiménez-Sánchez S, Jiménez-García R.

Prevalence of neck and low back pain in community-dwelling adults in Spain: a population-based national study. Spine. 2011; 36(3): E213-219.

Carmona L, Ballina J, Gabriel R, Laffon A, EPISER Study Group. The burden of musculoskeletal diseases in the general population of Spain: results from a national survey. Ann Rheum Dis. 2001; 60(11): 1040-5.

Ministry of Health (Chile). Chile National Health Survey 2009-2010.

Bredkjaer SR. Musculoskeletal disease in Denmark. The Danish Health and Morbidity Survey 1986-87. Acta Orthop Scand Suppl. 1991; 62(S241): 10-2.

Guez M, Hildingsson C, Nasic S, Toolanen G. Chronic low back pain in individuals with chronic neck pain of traumatic and non-traumatic origin: a population-based study. Acta Orthop. 2006; 77(1): 132-7.

Roth-Isigkeit A, Thyen U, Raspe HH, Stöven H, Schmucker P. Reports of pain among German children and adolescents: an epidemiological study. Acta Paediatr. 2004; 93(2): 258-63.

Erdine S. Pain Prevalence Among Adults in Turkey. Agri. 2004; 13(2-3): 22-30.

Kohlmann T, Deck R, Raspe H. Prevalence and severity of back pain in Lubeck. Aktuelle Rheumatol. 1995; 20(3): 99-104.

Liira JP, Shannon HS, Chambers LW, Haines TA. Long-term back problems and physical work exposures in the 1990 Ontario Health Survey. Am J Public Health. 1996; 86(3): 382-7.

Darmawan J, Valkenburg HA, Muirden KD, Wigley RD. Epidemiology of rheumatic diseases in rural and urban populations in Indonesia: a World Health Organisation International League Against Rheumatism COPCORD study, stage I, phase 2. Ann Rheum Dis. 1992; 51(4): 525-8.

Gourmelen J, Chastang J-F, Ozguler A, Lanoë J-L, Ravaud J-F, Leclerc A. Frequency of low back pain among men and women aged 30 to 64 years in France. Results of two national surveys. Ann Readapt Med Phys. 2007; 50(8): 633-44.

Malek J, Prikazsk V, Kurziva A, Kozak J, Lengalova E. Prevalence of pain in the Czech Republic ‚Äì A pilot study. Bolest. 2003; 6(2): 113-22.

Palmer KT, Walsh K, Bendall H, Cooper C, Coggon D. Back pain in Britain: comparison of two prevalence surveys at an interval of 10 years. BMJ. 2000; 320(7249): 1577-8.

Biering-Sørensen F. Low back trouble in a general population of 30-, 40-, 50-, and 60-year-old men and women. Study design, representativeness and basic results. Dan Med Bull. 1982; 29(6): 289-99.

Spahn G, Schiele R, Langlotz A, Jung R. Prevalence of functional pain of the back, the hip and the knee in adolescents. Results of a cross-sectional study. Dtsch Med Wochenschr. 2004; 129(43): 2285-90.

Galal SB, Hamad S, Hassan N. Self-reported adolescents' health and gender: an Egyptian study. East Mediterr Health J. 2001; 7(4-5): 625-34.

Miró J, Paredes S, Rull M, Queral R, Miralles R, Nieto R, Huguet A, Baos J. Pain in older adults: a prevalence study in the Mediterranean region of Catalonia. Eur J Pain. 2007; 11(1): 83-92.

Catala E, Reig E, Artés M, Aliaga L, López JS, Segú JL. Prevalence of pain in the Spanish population: telephone survey in 5000 homes. Eur J Pain. 2002; 6(2): 133-40.

Goubert L, Crombez G, De Bourdeaudhuij I. Low back pain, disability and back pain myths in a community sample: prevalence and interrelationships. Eur J Pain. 2004; 8(4): 385-94.

Bingefors K, Isacson D. Epidemiology, co-morbidity, and impact on health-related quality of life of self-reported headache and musculoskeletal pain ‚Äì a gender perspective. Eur J Pain. 2004; 8(5): 435-50.

Hüppe A, Müller K, Raspe H. Is the occurrence of back pain in Germany decreasing? Two regional postal surveys a decade apart. Eur J Public Health. 2007; 17(3): 318-22.

Hartvigsen J, Christensen K, Frederiksen H. Back pain remains a common symptom in old age. a population-based study of 4486 Danish twins aged 70-102. Eur Spine J. 2003; 12(5): 528-34.

Skoffer B, Foldspang A. Physical activity and low-back pain in schoolchildren. Eur Spine J. 2008; 17(3): 373-9.

Neuhauser H, Ellert U, Ziese T. Chronic back pain in the general population in Germany 2002/2003: prevalence and highly affected population groups. Gesundheitswesen. 2005; 67(10): 685-93.

Hakala P, Rimpelä A, Salminen JJ, Virtanen SM, Rimpelä M. Back, neck, and shoulder pain in Finnish adolescents: national cross sectional surveys. BMJ. 2002; 325(7367): 743.

Heistaro S, Vartiainen E, Heliövaara M, Puska P. Trends of back pain in eastern Finland, 1972-1992, in relation to socioeconomic status and behavioral risk factors. Am J Epidemiol. 1998; 148(7): 671-82.

Blay SL, Andreoli SB, Dewey ME, Gastal FL. Co-occurrence of chronic physical pain and psychiatric morbidity in a community sample of older people. Int J Geriatr Psychiatry. 2007; 22(9): 902-8.

Lau EM, Egger P, Coggon D, Cooper C, Valenti L, O'Connell D. Low back pain in Hong Kong: prevalence and characteristics compared with Britain. J Epidemiol Community Health. 1995; 49(5): 492-4.

Chopra A, Saluja M, Patil J, Tandale HS. Pain and disability, perceptions and beliefs of a rural Indian population: A WHO-ILAR COPCORD study. WHO-International League of Associations for Rheumatology. Community Oriented Program for Control of Rheumatic Diseases. J Rheumatol. 2002; 29(3): 614-21.

Croft PR, Rigby AS. Socioeconomic influences on back problems in the community in Britain. J Epidemiol Community Health. 1994; 48(2): 166-70.

Walker BF, Muller R, Grant WD. Low back pain in Australian adults: prevalence and associated disability. J Manipulative Physiol Ther. 2004; 27(4): 238-44.

Mohseni-Bandpei MA, Bagheri-Nesami M, Shayesteh-Azar M. Nonspecific low back pain in 5000 Iranian school-age children. J Pediatr Orthop. 2007; 27(2): 126-9.

Natvig B, Bruusgaard D, Eriksen W. Localized low back pain and low back pain as part of widespread musculoskeletal pain: two different disorders? A cross-sectional population study. J Rehabil Med. 2001; 33(1): 21-5.

Woo J, Ho SC, Lau J, Leung PC. Musculoskeletal complaints and associated consequences in elderly Chinese aged 70 years and over. J Rheumatol. 1994; 21(10): 1927-31.

Balagué F, Nordin M, Skovron ML, Dutoit G, Yee A, Waldburger M. Non-specific low-back pain among schoolchildren: a field survey with analysis of some associated factors. J Spinal Disord. 1994; 7(5): 374-9.

Elliott AM, Smith BH, Penny KI, Smith WC, Chambers WA. The epidemiology of chronic pain in the community. Lancet. 1999; 354(9186): 1248-52.

Leino PI, Berg MA, Puska P. Is back pain increasing? Results from national surveys in Finland during 1978/9-1992. Scand J Rheumatol. 1994; 23(5): 269-76.

Pinto-Meza A, Serrano-Blanco A, Codony M, Reneses B, von Korff M, Haro JM, Alonso J. Prevalence and physical-mental comorbidity of chronic back and neck pain in Spain: results from the ESEMeD Study. Med Clin (Barc). 2006; 127(9): 325-30.

Laslett M, Crothers C, Beattie P, Cregten L, Moses A. The frequency and incidence of low back pain/sciatica in an urban population. N Z Med J. 1991; 104(921): 424-6.

Pikó B. Epidemiology of psychosomatic symptoms and subjective health evaluation among secondary school students. Orv Hetil. 1999; 140(23): 1297-304.

Currie SR, Wang J. Chronic back pain and major depression in the general Canadian population. Pain. 2004; 107(1-2): 54-60.

Grimmer K, Nyland L, Milanese S. Longitudinal investigation of low back pain in Australian adolescents: a five-year study. Physiother Res Int. 2006; 11(3): 161-72.

Picavet HS, Schouten JS. Physical load in daily life and low back problems in the general population-The MORGEN study. Prev Med. 2000; 31(5): 506-12.

Jajiƒá I, Suƒáur A. [Backache in the population]. Reumatizam. 1985; 32(1-2): 5-9.

Mendoza-Sassi R, Béria JU, Fiori N, Bortolotto A. Prevalence of signs and symptoms, associated sociodemographic factors and resulting actions in an urban center in southern Brazil. Rev Panam Salud Publica. 2006; 20(1): 22-8.

Harkness EF, Macfarlane GJ, Silman AJ, McBeth J. Is musculoskeletal pain more common now than 40 years ago?: Two population-based cross-sectional studies. Rheumatology (Oxford). 2005; 44(7): 890-5.

Ballina Garcia FJ, Hernandez Mejía R, Martín Lascuevas P, Fernandez Santana J, Cueto Espinar A. Epidemiology of musculoskeletal complaints and use of health services in Asturias, Spain. Scand J Rheumatol. 1994; 23(3): 137-41.

Reigo T, Timpka T, Tropp H. The epidemiology of back pain in vocational age groups. Scand J Prim Health Care. 1999; 17(1): 17-21.

Kristjánsdóttir G. Prevalence of pain combinations and overall pain: a study of headache, stomach pain and back pain among school-children.

Scand J Soc Med. 1997; 25(1): 58-63.

Shiri R, Solovieva S, Husgafvel-Pursiainen K, Taimela S, Saarikoski LA, Huupponen R, Viikari J, Raitakari OT, Viikari-Juntura E. The association between obesity and the prevalence of low back pain in young adults: the Cardiovascular Risk in Young Finns Study. Am J Epidemiol. 2008; 167(9): 1110-9.

Papageorgiou AC, Croft PR, Ferry S, Jayson MI, Silman AJ. Estimating the prevalence of low back pain in the general population. Evidence from the South Manchester Back Pain Survey. Spine. 1995; 20(17): 1889-94.

Cassidy JD, Carroll LJ, Côté P. The Saskatchewan health and back pain survey. The prevalence of low back pain and related disability in Saskatchewan adults. Spine. 1998; 23(17): 1860-67.

Santos-Eggimann B, Wietlisbach V, Rickenbach M, Paccaud F, Gutzwiller F. One-year prevalence of low back pain in two Swiss regions: estimates from the population participating in the 1992-1993 MONICA project. Spine. 2000; 25(19): 2473-9.

Stranjalis G, Tsamandouraki K, Sakas DE, Alamanos Y. Low back pain in a representative sample of Greek population: analysis according to personal and socioeconomic characteristics. Spine. 2004; 29(12): 1355-61.

Jacob T, Baras M, Zeev A, Epstein L. A longitudinal, community-based study of low back pain outcomes. Spine. 2004; 29(16): 1810-7.

Cecchi F, Debolini P, Lova RM, Macchi C, Bandinelli S, Bartali B, Lauretani F, Benvenuti E, Hicks G, Ferrucci L. Epidemiology of back pain in a representative cohort of Italian persons 65 years of age and older: the InCHIANTI study. Spine. 2006; 31(10): 1149-55.

Oksuz E. Prevalence, risk factors, and preference-based health states of low back pain in a Turkish population. Spine. 2006; 31(25): E968-972.

Schmidt CO, Raspe H, Pfingsten M, Hasenbring M, Basler HD, Eich W, Kohlmann T. Back pain in the German adult population: prevalence, severity, and sociodemographic correlates in a multiregional survey. Spine. 2007; 32(18): 2005-11.

Taimela S, Kujala UM, Salminen JJ, Viljanen T. The prevalence of low back pain among children and adolescents. A nationwide, cohort-based questionnaire survey in Finland. Spine. 1997; 22(10): 1132-6.

Brage S, Laerum E. Spinal disorders in Norway ‚Äì an epidemiological report. Tidsskr Nor Laegeforen. 1999; 119(11): 1619-23.

Omokhodion FO. Low back pain in an urban population in Southwest Nigeria. Trop Doct. 2004; 34(1): 17-20.

Alvarez-Nemegyei J, Pelaez-Ballestas I, Sanin LH, Cardiel MH, Ramirez-Angulo A, Goycochea-Robles M-V. Prevalence of musculoskeletal pain and rheumatic diseases in the southeastern region of Mexico. A COPCORD-based community survey. J Rheumatol. 2011; 86(Suppl): 21-5.

Chaaya M, Slim ZN, Habib RR, Arayssi T, Dana R, Hamdan O, Assi M, Issa Z, Uthman I. High burden of rheumatic diseases in Lebanon: a COPCORD study. Int J Rheum Dis. 2012; 15(2): 136-43.

Silva MCD, Fassa AG, Valle NCJ. [Chronic low back pain in a Southern Brazilian adult population: prevalence and associated factors]. Cad Saude Publica. 2004; 20(2): 377-85.

Svensson HO, Andersson GB, Johansson S, Wilhelmsson C, Vedin A. A retrospective study of low-back pain in 38- to 64-year-old women. Frequency of occurrence and impact on medical services. Spine. 1988; 13(5): 548-52.

Walsh K, Cruddas M, Coggon D. Low back pain in eight areas of Britain. J Epidemiol Community Health. 1992; 46(3): 227-30.

World Health Organization Regional Office for Europe (EURO-WHO). Health Behaviour in School-aged Children: WHO Collaborative Cross-National survey/study (HBSC) 2006.

Institute for Social Research, University of Michigan. Cambodia Elderly Survey 2004. Ann Arbor, United States of America: Institute for Social Research, University of Michigan.

Wright D, Barrow S, Fisher AD, Horsley SD, Jayson MI. Influence of physical, psychological and behavioural factors on consultations for back pain. Br J Rheumatol. 1995; 34(2): 156-61.

Sutton-Tyrrell, Kim, Faith Selzer, MaryFran Sowers, Robert Neer, Lynda Powell, Ellen Gold, Gail Greendale, Gerson Weiss, Karen Matthews, and Sonja McKinlay. Study of Women's Health Across the Nation (SWAN), 1997-1999: Visit 01 Dataset. ICPSR29221-v1. Ann Arbor, MI: Inter-university Consortium for Political and Social Research[distributor], 2014-02-05. http://doi.org/10.3886/ICPSR29221.v1

Sutton-Tyrrell, Kim, Faith Selzer, MaryFran Sowers, Robert Neer, Lynda Powell, Ellen Gold, Gail Greendale, Gerson Weiss, Karen Matthews, and Sonja McKinlay. Study of Women's Health Across the Nation (SWAN), 1999-2001: Visit 03 Dataset. ICPSR29701-v1. Ann Arbor, MI: Inter-university Consortium for Political and Social Research [distributor], 2014-02-12. http://doi.org/10.3886/ICPSR29701.v1

Sutton-Tyrell, Kim, Faith Selzer, MaryFran Sowers, Robert Neer, Lynda Powell, Ellen Gold, Gail Greendale, Gerson Weiss, Karen Matthews, and Sonja McKinlay. Study of Women's Health Across the Nation (SWAN), 2000-2002: Visit 04 Dataset. ICPSR30142-v1. Ann Arbor, MI: Inter-

university Consortium for Political and Social Research[distributor], 2014-02-13. http://doi.org/10.3886/ICPSR30142.v1

Sutton-Tyrell, Kim, Faith Selzer, MaryFran Sowers, Robert Neer, Lynda Powell, Ellen Gold, Gail Greendale, Gerson Weiss, Karen Matthews, and Sonja McKinlay. Study of Women's Health Across the Nation (SWAN), 2001-2003: Visit 05 Dataset. ICPSR30501-v1. Ann Arbor, MI: Inter-university Consortium for Political and Social Research [distributor], 2014-09-02. http://doi.org/10.3886/ICPSR30501.v1

Sutton-Tyrell, Kim, Faith Selzer, MaryFran Sowers, Joel Finkelstein, Lynda Powell, Ellen Gold, Gail Greendale, Gerson Weiss, and Karen Matthews. Study of Women's Health Across the Nation (SWAN), 2002-2004: Visit 06 Dataset. ICPSR31181-v1. Ann Arbor, MI: Inter-university Consortium for Political and Social Research [distributor], 2014-09-24. http://doi.org/10.3886/ICPSR31181.v1

Sutton-Tyrell, Kim, Faith Selzer, MaryFran Sowers, Joel Finkelstein, Lynda Powell, Ellen Gold, Gail Greendale, Gerson Weiss, and Karen Matthews. Study of Women's Health Across the Nation (SWAN), 2003-2005: Visit 07 Dataset. ICPSR31901-v1. Ann Arbor, MI: Inter-university Consortium for Political and Social Research [distributor], 2014-09-30. http://doi.org/10.3886/ICPSR31901.v1

Sutton-Tyrell, Kim, Faith Selzer, MaryFran Sowers, Joel Finkelstein, Lynda Powell, Ellen Gold, Gail Greendale, Gerson Weiss, and Karen Matthews. Study of Women's Health Across the Nation (SWAN), 2004-2006: Visit 08 Dataset. ICPSR32122-v1. Ann Arbor, MI: Inter-university Consortium for Political and Social Research [distributor], 2014-09-30. http://doi.org/10.3886/ICPSR32122.v1

Sutton-Tyrell, Kim, Faith Selzer, MaryFran Sowers, Joel Finkelstein, Lynda Powell, Ellen Gold, Gail Greendale, Gerson Weiss, and Karen Matthews. Study of Women's Health Across the Nation (SWAN), 2005-2007: Visit 09 Dataset. ICPSR32721-v1. Ann Arbor, MI: Inter-university Consortium for Political and Social Research [distributor], 2014-09-30. http://doi.org/10.3886/ICPSR32721.v1

Sutton-Tyrrell, Kim, Faith Selzer, MaryFran Sowers, Joel Finkelstein, Lynda Powell, Ellen Gold, Gail David, Gerson Weiss, and Karen Matthews. Study of Women Across the Nation (SWAN), 2006-2008: Visit 10 Dataset. ICPSR32961-v1. Ann Arbor, MI: Inter-university Consortium for Political and Social Research [distributor], 2014-10-08. http://doi.org/10.3886/ICPSR32961.v1

World Health Organization Regional Office for Europe (EURO-WHO). Health Behaviour in School-aged Children: WHO Collaborative Cross-National survey/study (HBSC) 1998.

World Health Organization Regional Office for Europe (EURO-WHO). Health Behaviour in School-aged Children: WHO Collaborative Cross-National survey/study (HBSC) 1997-1998.

World Health Organization Regional Office for Europe (EURO-WHO). Health Behaviour in School-aged Children: WHO Collaborative Cross-National survey/study (HBSC) 1997.

World Health Organization Regional Office for Europe (EURO-WHO). Health Behaviour in School-aged Children: WHO Collaborative Cross-National survey/study (HBSC) 2001.

World Health Organization Regional Office for Europe (EURO-WHO). Health Behaviour in School-aged Children: WHO Collaborative Cross-National survey/study (HBSC) 2002.

World Health Organization Regional Office for Europe (EURO-WHO). Health Behaviour in School-aged Children: WHO Collaborative Cross-National survey/study (HBSC) 2001-2002.

World Health Organization Regional Office for Europe (EURO-WHO). Health Behaviour in School-aged Children: WHO Collaborative Cross-National survey/study (HBSC) 2005-2006.

World Health Organization Regional Office for Europe (EURO-WHO). Health Behaviour in School-aged Children: WHO Collaborative Cross-National survey/study (HBSC) 2005.

World Health Organization Regional Office for Europe (EURO-WHO). Health Behaviour in School-aged Children: WHO Collaborative Cross-National survey/study (HBSC) 2010.

World Health Organization Regional Office for Europe (EURO-WHO). Health Behaviour in School-aged Children: WHO Collaborative Cross-National survey/study (HBSC) 2009-2010.

World Health Organization Regional Office for Europe (EURO-WHO). Health Behaviour in School-aged Children: WHO Collaborative Cross-National survey/study (HBSC) 2010-2011.

World Health Organization Regional Office for Europe (EURO-WHO). Health Behaviour in School-aged Children: WHO Collaborative Cross-National survey/study (HBSC) 2009.

Stahl MK, El-Metwally AA, Rimpela AH. Time trends in single versus concomitant neck and back pain in Finnish adolescents: results from national cross-sectional surveys from 1991 to 2011. BMC Musculoskelet Disord. 2014; 296.

Aartun E, Hartvigsen J, Wedderkopp N, Hestbaek L. Spinal pain in adolescents: prevalence, incidence, and course: a school-based two-year prospective cohort study in 1,300 Danes aged 11-13. BMC Musculoskelet Disord. 2014; 187.

Mesas AE, Gonzalez AD, Mesas CE, de Andrade SM, Magro IS, del Llano J. The association of chronic neck pain, low back pain, and migraine with absenteeism due to health problems in Spanish workers. Spine. 2014; 39(15): 1243-53.

Yamada K, Matsudaira K, Takeshita K, Oka H, Hara N, Takagi Y. Prevalence of low back pain as the primary pain site and factors associated with low health-related quality of life in a large Japanese population: a pain-associated cross-sectional epidemiological survey. Mod Rheumatol. 2014; 24(2): 343-8.

Ghanei I, Rosengren BE, Hasserius R, Nilsson J, Mellstrom D, Ohlsson C, Ljunggren, Karlsson MK. The prevalence and severity of low back pain and associated symptoms in 3,009 old men. Eur Spine J. 2014; 23(4): 814-20.

Chou YC, Shih CC, Lin JG, Chen TL, Liao CC. Low back pain associated with sociodemographic factors, lifestyle and osteoporosis: a population-based study. J Rehabil Med. 2013; 45(1): 76-80.

Vargas-Prada S, Serra C, Mart¬°nez JM, Ntani G, Delclos GL, Palmer KT, Coggon D, Benavides FG. Psychological and culturally-influenced risk factors for the incidence and persistence of low back pain and associated disability in Spanish workers: findings from the CUPID study. Occup Environ Med. 2013; 70(1): 57-62.

Ono R, Higashi T, Takahashi O, Tokuda Y, Shimbo T, Endo H, Hinohara S, Fukui T, Fukuhara S. Sex differences in the change in health-related quality of life associated with low back pain. Qual Life Res. 2012; 21(10): 1705-11.

Plouvier S, Gourmelen J, Chastang JF, Lanoe JL, Leclerc A. Low back pain around retirement age and physical occupational exposure during working life. BMC Public Health. 2011; 268.

Woo J, Leung J, Lau E. Prevalence and correlates of musculoskeletal pain in Chinese elderly and the impact on 4-year physical function and quality of life. Public Health. 2009; 123(8): 549-56.

Masiero S, Carraro E, Celia A, Sarto D, Ermani M. Prevalence of nonspecific low back pain in schoolchildren aged between 13 and 15 years. Acta Paediatr. 2008; 97(2): 212-6.

Cecchi F, Mannoni A, Molino-Lova R, Ceppatelli S, Benvenuti E, Bandinelli S, Lauretani F, Macchi C, Ferrucci L. Epidemiology of hip and knee pain in a community based sample of Italian persons aged 65 and older. Osteoarthr Cartil. 2008; 16(9): 1039-46.

Pellise,Äö F, Balague,Äö F, Rajmil L, Cedraschi C, Aguirre M, Fontecha CG, Pasarin M, Ferrer M. Prevalence of low back pain and its effect on health-related quality of life in adolescents. Arch Pediatr Adolesc Med. 2009; 163(1): 65-71.

Hartvigsen J, Christensen K. Pain in the back and neck are with us until the end: a nationwide interview-based survey of Danish 100-year-olds. Spine. 2008; 33(8): 909-13.

Liao ZT, Pan YF, Huang JL, Huang F, Chi WJ, Zhang KX, Lin ZM, Wu YQ, He WZ, Wu J, Xie XJ, Huang JX, Wei QJ, Li TW, Wu Z, Yu BY, Gu JR. An epidemiological survey of low back pain and axial spondyloarthritis in a Chinese Han population. Scand J Rheumatol. 2009; 38(6): 455-9.

Horvath G, Koroknai G, Acs B, Than P, Ill‚Äös T. Prevalence of low back pain and lumbar spine degenerative disorders. Questionnaire survey and clinical-radiological analysis of a representative Hungarian population. Int Orthop. 2010; 34(8): 1245-9.

Leijon O, Mulder M. Prevalence of low back pain and concurrent psychological distress over a 16-year period. Occup Environ Med. 2009; 66(2): 137-9.

Bjorck-van Dijken C, Fjellman-Wiklund A, Hildingsson C. Low back pain, lifestyle factors and physical activity: a population based-study. J Rehabil Med. 2008; 40(10): 864-9.

Trevelyan FC, Legg SJ. The prevalence and characteristics of back pain among school children in New Zealand. Ergonomics. 2010; 53(12): 1455-60.

Jimenez-Sanchez S, Fernandez-de-Las-Penas C, Carrasco-Garrido P, Hernandez-Barrera V, Alonso-Blanco C, Palacios-Cena D, Jimenez-Garcia R. Prevalence of chronic head, neck and low back pain and associated factors in women residing in the Autonomous Region of Madrid (Spain). Gac Sanit. 2012; 26(6): 534-40.

Gao HL, Lin SQ, Wei Y, Chen Y, Wu ZL. The effect of age and menopausal status on musculoskeletal symptoms in Chinese women aged 35-64 years. Climacteric. 2013; 16(6): 639-45.

Pedisic Z, Pranic S, Jurakic D. Relationship of back and neck pain with quality of life in the Croatian general population. J Manipulative Physiol Ther. 2013; 36(5): 267-75.

Lemeunier N, Leboeuf-Yde C, Kjaer P, Gagey O. Stability of low back pain reporting over 8 years in a general population aged 40/41 years at base-line: data from three consecutive cross-sectional surveys. BMC Musculoskelet Disord. 2013; 270.

van Oostrom SH, Monique Verschuren WM, de Vet HC, Picavet HS. Ten year course of low back pain in an adult population-based cohort--the

Doetinchem cohort study. Eur J Pain. 2011; 15(9): 993-8.

El-Sayed AM, Hadley C, Tessema F, Tegegn A, Cowan JA, Galea S. Back and neck pain and psychopathology in rural sub-Saharan Africa: evidence from the Gilgel Gibe Growth and Development Study, Ethiopia. Spine. 2010; 35(6): 684-9.

Skillgate E, Magnusson C, Lundberg M, Hallqvist J. The age- and sex-specific occurrence of bothersome neck pain in the general population--results from the Stockholm public health cohort. BMC Musculoskelet Disord. 2012; 185.

Minghelli B, Oliveira R, Nunes C. Association of obesity with chronic disease and musculoskeletal factors. Rev Assoc Med Bras (1992). 2015; 61(4): 347,Äì54.

Frilander H, Solovieva S, Mutanen P, Pihlajamaki H, Heliovaara M, Viikari-Juntura E. Role of overweight and obesity in low back disorders among men: a longitudinal study with a life course approach. BMJ Open. 2015; 5(8): e007805.

Adegoke BO, Odole AC, Adeyinka AA. Adolescent low back pain among secondary school students in Ibadan, Nigeria. Afr Health Sci. 2015; 15(2): 429-37.

Koyanagi A, Stickley A, Garin N, Miret M, Ayuso-Mateos JL, Leonardi M, Koskinen S, Galas A, Haro JM. The association between obesity and back pain in nine countries: a cross-sectional study. BMC Public Health. 2015; 15: 123.

Ono R, Yamazaki S, Takegami M, Suzukamo Y, Konno S, Kikuchi S, Fukuhara S. Patient-reported disability in the general Japanese population was associated with medical care visits for low back pain, regardless of pain intensity. J Orthop Sci. 2015; 20(4): 742-9.

Capkin E, Karkucak M, Cakirbay H, Topbas M, Karaca A, Kose MM, Gokmen F. The prevalence and risk factors of low back pain in the eastern Black Sea region of Turkey. J Back Musculoskelet Rehabil. 2015; 28(4): 783-7.

Teraguchi M, Yoshimura N, Hashizume H, Muraki S, Yamada H, Oka H, Minamide A, Nakagawa H, Ishimoto Y, Nagata K, Kagotani R, Tanaka S, Kawaguchi H, Nakamura K, Akune T, Yoshida M. The association of combination of disc degeneration, end plate signal change, and Schmorl node with low back pain in a large population study: the Wakayama Spine Study. Spine J. 2015; 15(4): 622-8.

Meziat Filho N, Coutinho ES, Azevedo e Silva G. Association between home posture habits and low back pain in high school adolescents. Eur Spine J. 2015; 24(3): 425-33.

Granados Y, Cedeno L, Rosillo C, Berbin S, Azocar M, Molina ME, Lara O, Sanchez G, Pelaez-Ballestas I. Prevalence of musculoskeletal

disorders and rheumatic diseases in an urban community in Monagas State, Venezuela: a COPCORD study. Clin Rheumatol. 2015; 34(5): 871-7.

Leclerc A, Chastang JF, Taiba R, Pascal P, Cyr D, Plouvier S, Descatha A. Musculoskeletal pain at various anatomical sites and socioeconomic position: Results of a national survey. Rev Epidemiol Sante Publique. 2016; 64(5): nan.

Dolphens M, Vansteelandt S, Cagnie B, Vleeming A, Nijs J, Vanderstraeten G, Danneels L. Multivariable modeling of factors associated with spinal pain in young adolescence. Eur Spine J. 2016; 25(9.0): 2809-21.

Noormohammadpour P, Mansournia MA, Koohpayehzadeh J, Asgari F, Rostami M, Rafei A, Kordi R. Prevalence of chronic neck pain, low back pain and knee pain and their related factors in community-dwelling adults in Iran: A population-based national study. Clin J Pain. 2016; 33(2): 181-7.

Ogwumike OO, Adeniyi AF, Orogbemi OO. Musculoskeletal pain among postmenopausal women in Nigeria: Association with overall and central obesity. Hong Kong Physiother J. 2016; 34: 41,Äì6.

Indian Council of Medical Research (ICMR), Ministry of Health and Family Welfare (India), Vardhman Mahavir Medical College and Safdarjung Hospital (India). India Survey on Musculoskeletal Conditions 2007-2010.

Statistics Norway. Norway Survey of Living Conditions on Health 2015.

Kolahi S, Khabbazi A, Malek Mahdavi A, Ghasembaglou A, Ghasembaglou A, Aminisani N, Somi MH, Heidari F. Prevalence of musculoskeletal disorders in Azar cohort population in Northwest of Iran. Rheumatol Int. 2017; 37(4): 495-502.

Depintor JD, Bracher ES, Cabral DM, Eluf-Neto J. Prevalence of chronic spinal pain and identification of associated factors in a sample of the population of Sao Paulo, Brazil: cross-sectional study. Sao Paulo Med J. 2016; 134(5): 375-384.

Billis E, Koutsojannis C, Matzaroglou C, Gliatis J, Fousekis K, Gioftsos G, Papandreou M, McCarthy C, Oldham JA, Tsepis E. Association of low back pain on physical, sociodemographic and lifestyle factors across a general population sample within Greece. J Back Musculoskelet Rehabil. 2017; 30(2): 279-290.

Noll M, Candotti CT, Rosa BN, Loss JF. Back pain prevalence and associated factors in children and adolescents: an epidemiological population study. Rev Saude Publica. 2016; 50.

Kamada M, Abe T, Kitayuguchi J, Imamura F, Lee IM, Kadowaki M, Sawada SS, Miyachi M, Matsui Y, Uchio Y. Dose-response relationship

between sports activity and musculoskeletal pain in adolescents. Pain. 2016; 157(6): 1339-45.

Bikbov MM, Fayzrakhmanov RR, Kazakbaeva GM, Zainullin RM, Salavatova VF, Gilmanshin TR, Arslangareeva II, Nikitin NA, Mukhamadieva SR, Yakupova DF, Khikmatullin RI, Aminev SK, Nuriev IF, Zaynetdinov AF, Uzianbaeva YV, Jonas JB.¬†Prevalence and Associated Factors of Low Backpain, Thoracic Spine Pain and Neck Pain in Russians: The Ural Eye and Medical Study. [Unpublished].

Guevara-Pacheco SV, Feican-Alvarado A, Delgado-Pauta J, Lliguisaca-Segarra A, Pelaez-Ballestas I. Prevalence of Disability in Patients With Musculoskeletal Pain and Rheumatic Diseases in a Population From Cuenca, Ecuador. J Clin Rheumatol. 2017; 23(6): 324,Äì9.

Guevara-Pacheco S, Feicán-Alvarado A, Sanín LH, Vintimilla-Ugalde J, Vintimilla-Moscoso F, Delgado-Pauta J, Lliguisaca-Segarra A, Dután-Erráez H, Guevara-Mosquera D, Ochoa-Robles V, Cardiel MH, Peláez-Ballestas I. Prevalence of musculoskeletal disorders and rheumatic diseases in Cuenca, Ecuador: a WHO-ILAR COPCORD study. Rheumatol Int. 2016; 36(9): 1195-1204.

Ministry of Health and Welfare (Taiwan). Taiwan National Health Insurance Claims Data 2016.

Marmot, M., Oldfield, Z., Clemens, S., Blake, M., Phelps, A., Nazroo, J., Steptoe, A., Rogers, N., Banks, J., Oskala, A. (2017). English Longitudinal Study of Ageing: Waves 0-7, 1998-2015. [data collection]. 27th Edition. UK Data Service. SN: 5050, http://doi.org/10.5255/UKDA-SN-5050-14

Central Statistical Agency (Ethiopia). Ethiopia Welfare Monitoring Survey 2015-2016.

D.G. Hoy, T. Raikoti, E. Smith, A. Tuzakana, T. Gill, K. Matikarai, J. Tako, A. Jorari, F. Blyth, A. Pitaboe, R. Buchbinder, I. Kalauma, P. Brooks, C. Lepers, A. Woolf, A. Briggs, and L. March. Use of The Global Alliance for Musculoskeletal Health survey module for estimating the population prevalence of musculoskeletal pain: findings from the Solomon Islands [Unpublished]. BMC Musculoskelet Disord. 2018; 19(292).

Akbar F, AlBesharah M, Al-Baghli J, Bulbul F, Mohammad D, Qadoura B, Al-Taiar A. Prevalence of low Back pain among adolescents in relation to the weight of school bags. BMC Musculoskelet Disord. 2019; 20(1): 37.

Badley EM, Millstone DB, Perruccio AV. Back Pain and Co-occurring Conditions: Findings From a Nationally Representative Sample. Spine. 2018; 43(16): E935-E941.

Ben Ayed H, Yaich S, Trigui M, Ben Hmida M, Ben Jemaa M, Ammar A, Jedidi J, Karray R, Feki H, Mejdoub Y, Kassis M, Damak J. Prevalence, Risk Factors and Outcomes of Neck, Shoulders and Low-Back Pain in Secondary-School Children. J Res Health Sci. 2019; 19(1): e00440.

Gonçalves TR, Mediano MFF, Sichieri R, Cunha DB. Is Health-related Quality of Life Decreased in Adolescents With Back Pain?. Spine. 2018; 43(14): E822-E829.

Horii C, Asai Y, Iidaka T, Muraki S, Oka H, Tsutsui S, Hashizume H, Yamada H, Yoshida M, Kawaguchi H, Nakamura K, Akune T, Tanaka S, Yoshimura N. Differences in prevalence and associated factors between mild and severe vertebral fractures in Japanese men and women: the third survey of the ROAD study. J Bone Miner Metab. 2019; 37(5): 844-853.

Laslett LL, Menz HB, Otahal P, Pan F, Cicuttini FM, Jones G. Factors associated with prevalent and incident foot pain: data from the Tasmanian Older Adult Cohort Study. Maturitas. 2018; 118: 38-43.

Muntaner-Mas A, Palou P, Ortega FB, Vidal-Conti J. Sports participation and low back pain in schoolchildren. J Back Musculoskelet Rehabil. 2018; 31(5): 811-819.

Noormohammadpour P, Borghei A, Mirzaei S, Mansournia MA, Ghayour-Najafabadi M, Kordi M, Kordi R. The Risk Factors of Low Back Pain in Female High School Students. Spine. 2019; 44(6): E357-E365.

Oka GA, Ranade AS, Kulkarni AA. Back pain and school bag weight - a study on Indian children and review of literature. J Pediatr Orthop B. 2019; 28(4): 397-404.

Scarabottolo CC, Pinto RZ, Oliveira CB, Zanuto EF, Cardoso JR, Christofaro DGD. Back and neck pain prevalence and their association with physical inactivity domains in adolescents. Eur Spine J. 2017; 26(9): 2274-2280.

Schauer B, Grabe HJ, Ittermann T, Lerch MM, Weiss FU, Mönnikes H, Völzke H, Enck P, Schwille-Kiuntke J. Irritable bowel syndrome, mental health, and quality of life: Data from a population-based survey in Germany (SHIP-Trend-0). Neurogastroenterol Motil. 2019; 31(3): e13511.

Sundell CG, Bergström E, Larsén K. Low back pain and associated disability in Swedish adolescents. Scand J Med Sci Sports. 2019; 29(3): 393-399.

Ho KKN, Simic M, Cvancarova Småstuen M, de Barros Pinheiro M, Ferreira PH, Bakke Johnsen M, Heuch I, Grotle M, Zwart JA, Nilsen KB. The association between insomnia, c-reactive protein, and chronic low back pain: cross-sectional analysis of the HUNT study, Norway. Scand J Pain. 2019; 19(4): 765-77.

Keeratisiroj O, Siritaratiwat W. Prevalence of self-reported musculoskeletal pain symptoms among school-age adolescents: age and sex differences. Scand J Pain. 2018; 18(2): 273-80.

Stockil L, Thompson J, Briffa K, Smith A, Beales D, Straker L, O'Sullivan P, Jacques A. Urogenital symptoms: prevalence, bother, associations and impact in 22year-old women of the Raine Study. Int Urogynecol J. 2018; 29(12): 1807-1815.

Wang XD, Ma L, Wang DH, Yan JT. Relationships among the lumbar lordosis index, sacral horizontal angle, and chronic low back pain in the elderly aged 60-69 years: A cross-sectional study. J Back Musculoskelet Rehabil. 2020; 33(1): 29-33.

Bedene A, Lijfering WM, Niesters M, van Velzen M, Rosendaal FR, Bouvy ML, Dahan A, van Dorp ELA. Opioid Prescription Patterns and Risk Factors Associated With Opioid Use in the Netherlands. JAMA Netw Open. 2019; 2(8): e1910223.

El-Metwally A, Shaikh Q, Aldiab A, Al-Zahrani J, Al-Ghamdi S, Alrasheed AA, Househ M, Da'ar OB, Nooruddin S, Razzak HA, Aldossari KK. The prevalence of chronic pain and its associated factors among Saudi Al-Kharj population; a cross sectional study. BMC Musculoskelet Disord. 2019; 20(1): 177.

Endo T, Abe T, Akai K, Kijima T, Takeda M, Yamasaki M, Isomura M, Nabika T, Yano S. Height loss but not body composition is related to low back pain in community-dwelling elderlies: Shimane CoHRE study. BMC Musculoskelet Disord. 2019; 20(1): 207.

Fink HA, Litwack-Harrison S, Ensrud KE, Shen J, Schousboe JT, Cawthon PM, Cauley JA, Lane NE, Taylor BC, Barrett-Connor E, Kado DM, Cummings SR, Marshall LM, Osteoporotic Fractures in Men (MrOS) Study Group. Association of Incident, Clinically Undiagnosed Radiographic Vertebral Fractures With Follow-Up Back Pain Symptoms in Older Men: the Osteoporotic Fractures in Men (MrOS) Study. J Bone Miner Res. 2017; 32(11): 2263-2268.

Galozzi P, Maghini I, Bakdounes L, Ferlito E, Lazzari V, Ermani M, Chia M, Gatti D, Masiero S, Punzi L. Prevalence of low back pain and its effect on health-related quality of life in 409 scholar adolescents from the Veneto region. Reumatismo. 2019; 71(3): 132-140.

Iizuka Y, Iizuka H, Mieda T, Tsunoda D, Sasaki T, Tajika T, Yamamoto A, Takagishi K. Prevalence of Chronic Nonspecific Low Back Pain and Its Associated Factors among Middle-Aged and Elderly People: An Analysis Based on Data from a Musculoskeletal Examination in Japan. Asian Spine J. 2017; 11(6): 989-97.

Ikeda T, Sugiyama K, Aida J, Tsuboya T, Watabiki N, Kondo K, Osaka K. Socioeconomic inequalities in low back pain among older people: the JAGES cross-sectional study. Int J Equity Health. 2019; 18(1): 15.

Joergensen AC, Hestbaek L, Andersen PK, Nybo Andersen AM. Epidemiology of spinal pain in children: a study within the Danish National Birth Cohort. Eur J Pediatr. 2019; 178(5): 695-706.

Kikuchi R, Hirano T, Watanabe K, Sano A, Sato T, Ito T, Endo N, Tanabe N. Gender differences in the prevalence of low back pain associated with sports activities in children and adolescents: a six-year annual survey of a birth cohort in Niigata City, Japan. BMC Musculoskelet Disord. 2019; 20(1): 327.

Kędra A, Kolwicz-Gańko A, Sitarski D, Kędra P, Czaprowski D. Prevalence of back pain and the knowledge of preventive measures in a cohort of 11619 Polish school-age children and youth-an epidemiological study. Medicine (Baltimore). 2019; 98(22): e15729.

Paranjape S, Ingole V. Prevalence of Back Pain in Secondary School Students in an Urban Population: Cross-sectional Study. Cureus. 2018; 10(7): e2983.

Puth MT, Klaschik M, Schmid M, Weckbecker K, Münster E. Prevalence and comorbidity of osteoporosis- a cross-sectional analysis on 10,660 adults aged 50years and older in Germany. BMC Musculoskelet Disord. 2018; 19(1): 144.

Sasaki T, Yoshimura N, Hashizume H, Yamada H, Oka H, Matsudaira K, Iwahashi H, Shinto K, Ishimoto Y, Nagata K, Teraguchi M, Kagotani R, Muraki S, Akune T, Tanaka S, Kawaguchi H, Nakamura K, Minamide A, Nakagawa Y, Yoshida M. MRI-defined paraspinal muscle morphology in Japanese population: The Wakayama Spine Study. PLoS One. 2017; 12(11): e0187765.

Saraiva BTC, Pinto RZ, Oliveira CB, Zanuto EF, Scarabottolo CC, Delfino LD, Suetake VYB, Gil FCS, Christofaro DGD. Continuity of physical activity practice from childhood to adolescence is associated with lower neck pain in both sexes and lower back pain in girls. J Back Musculoskelet Rehabil. 2020; 33(2): 269-275.

Bento TPF, Genebra CVDS, Maciel NM, Cornelio GP, Simeão SFAP, Vitta A. Low back pain and some associated factors: is there any difference between genders?. Braz J Phys Ther. 2020; 24(1): 79-87.

Heuch I, Heuch I, Hagen K, Sørgjerd EP, Åsvold BO, Zwart JA. Is chronic low back pain a risk factor for diabetes? The Nord-Trøndelag Health Study. BMJ Open Diabetes Res Care. 2018; 6(1): e000569.

Schwertner DS, Oliveira RANS, Koerich MHAL, Motta AF, Pimenta AL, Gioda FR. Prevalence of low back pain in young Brazilians and associated factors: Sex, physical activity, sedentary behavior, sleep and body mass index. J Back Musculoskelet Rehabil. 2020; 33(2): 233-244.
